# Berberine-microbiota interplay: orchestrating gut health through modulation of the gut microbiota and metabolic transformation into bioactive metabolites

**DOI:** 10.3389/fphar.2023.1281090

**Published:** 2023-12-07

**Authors:** Tessa Dehau, Marc Cherlet, Siska Croubels, Michiel Van De Vliet, Evy Goossens, Filip Van Immerseel

**Affiliations:** ^1^ Livestock Gut Health Team (LiGHT) Ghent, Department of Pathobiology, Pharmacology and Zoological Medicine, Faculty Of Veterinary Medicine, Merelbeke, Belgium; ^2^ Laboratory of Pharmacology and Toxicology, Department of Pathobiology, Pharmacology and Zoological Medicine, Faculty of Veterinary Medicine, Ghent University, Merelbeke, Belgium; ^3^ Laboratory of Microbiology, Department of Biochemistry and Microbiology, Faculty of Sciences, Ghent University, Ghent, Belgium

**Keywords:** berberine, gut microbiota, berberrubine, demethylation, thalifendine, blautia

## Abstract

Berberine is an isoquinoline alkaloid found in plants. It presents a wide range of pharmacological activities, including anti-inflammatory and antioxidant properties, despite a low oral bioavailability. Growing evidence suggests that the gut microbiota is the target of berberine, and that the microbiota metabolizes berberine to active metabolites, although little evidence exists in the specific species involved in its therapeutic effects. This study was performed to detail the bidirectional interactions of berberine with the broiler chicken gut microbiota, including the regulation of gut microbiota composition and metabolism by berberine and metabolization of berberine by the gut microbiota, and how they contribute to berberine-mediated effects on gut health. As previous evidence showed that high concentrations of berberine may induce dysbiosis, low (0.1 g/kg feed), middle (0.5 g/kg feed) and high (1 g/kg feed) doses were here investigated. Low and middle doses of in-feed berberine stimulated potent beneficial bacteria from the Lachnospiraceae family in the large intestine of chickens, while middle and high doses tended to increase villus length in the small intestine. Plasma levels of the berberine-derived metabolites berberrubine, thalifendine and demethyleneberberine were positively correlated with the villus length of chickens. Berberrubine and thalifendine were the main metabolites of berberine in the caecum, and they were produced *in vitro* by the caecal microbiota, confirming their microbial origin. We show that members of the genus *Blautia* could demethylate berberine into mainly thalifendine, and that this reaction may stimulate the production of short-chain fatty acids (SCFAs) acetate and butyrate, via acetogenesis and cross-feeding respectively. We hypothesize that acetogens such as *Blautia* spp. are key bacteria in the metabolization of berberine, and that berberrubine, thalifendine and SCFAs play a significant role in the biological effect of berberine.

## Introduction

Alkaloids are one of the most important classes of plant bioactives. Among these, the class of isoquinoline alkaloids exhibits important biological activities, alongside very limited side effects, which make them attracting candidates for drug development. Berberine is a well-known representative of this class and displays multiple pharmacological properties in humans and animals, including anti-inflammatory, antioxidant, antihyperglycemic and antihyperlipidemic activities (Habtemariam, 2016; Jin et al., 2016; Zou et al., 2017). However, despite a wide spectrum of systemic pharmacological effects, its oral bioavailability is extremely low and can therefore hardly explain these effects. As after oral administration, most of the berberine remains in the intestinal lumen, it is widely suggested that its mode of action relies on the modulation of the gut microbiota composition and metabolism (Zhang et al., 2020a). The gut microbiota has the capability to synthesize a range of metabolites that are essential for maintaining normal body functions, such as short-chain fatty acids (SCFAs), trimethylamine, tryptophan, secondary bile acids and branched chain amino acids. Berberine has been shown to change the intestinal metabolite profile by regulating the composition and functionality of the microbiota (Zhang et al., 2012; Zhang et al., 2020c; Li et al., 2021; Shu et al., 2021; Yan et al., 2022) (for review Cheng et al., 2022). In addition, after oral administration, berberine is metabolized, mainly in the intestinal tract and liver, into berberine-derived metabolites, which are thought to contribute to berberine’s action as well as influence berberine’s bioavailability *in vivo* (Wang et al., 2017b).

**FIGURE 1:**
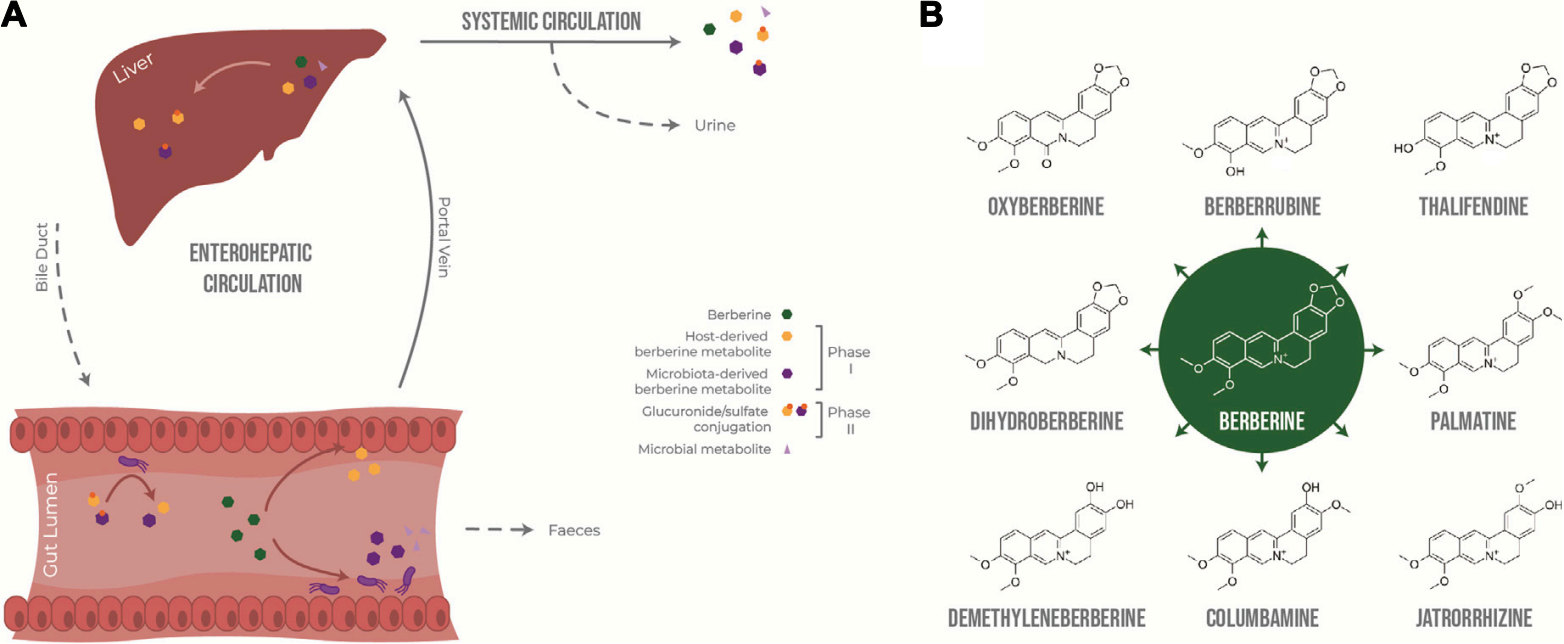
Metabolism of berberine after oral administration. **(A)** Transformation of berberine by the host and the gut microbiota. **(B)** Main phase I metabolites of berberine *in vivo*.

In the intestine, berberine goes through phase I metabolism, including reduction, demethylation and oxidation, mediated by enzymes expressed by enterocytes and gut bacteria, including cytochrome P450 isoenzymes (CYP450). Because berberine has low lipophilicity and is a substrate of the efflux transporter P-glycoprotein (Zhang et al., 2019), only a very small fraction of the initial dose is absorbed through the gut epithelium and transported to the liver via the portal vein, together with phase I metabolites. The liver is a site of phase I and phase II metabolism, where phase I metabolites are conjugated to glucuronide or sulfate molecules (Tan et al., 2013). Following exposure to the liver’s rich collection of metabolic enzymes, berberine and its derived metabolites are either excreted via the biliary duct or enter systemic circulation to distribute into tissues or to be excreted through the kidneys into the urine. Phase II metabolites excreted in the bile can re-enter the gut as they can be deconjugated by bacterial glucuronidases and sulfatases, resulting in the original phase I metabolites, thus resulting in entero-hepatic recycling (Figure 1A).

Berberrubine, thalifendine, demethyleneberberine, jatrorrhizine, columbamine and palmatine have been described as the main phase I metabolites of berberine after oral administration in rats (Liu et al., 2010; Ma et al., 2013; Tan et al., 2013; Wang et al., 2017b; Feng et al., 2021) and humans (Spinozzi et al., 2014) (Figure 1B). Thalifendine and demethyleneberberine were the most permeable metabolites across Caco-2 monolayers *in vitro* (Zhang et al., 2019). Demethyleneberberine glucuronide, berberrubine glucuronide or thalifendine glucuronide were the most abundant circulating metabolites in plasma after oral administration of berberine in rats or beagle dogs, highlighting that demethylation and demethylenation and subsequent glucuronidation are important metabolic pathways of berberine (Liu et al., 2009; Liu et al., 2010; Feng et al., 2018; Feng et al., 2021). Berberine metabolites are pharmacologically active, and because of their greater absorption or systemic presence, they might be partly responsible for the biological effects of berberine *in vivo* (Qiang et al., 2016; Chen et al., 2017; Yang et al., 2017; Yu et al., 2018; Zhang et al., 2020b; Sun et al., 2021; Yang et al., 2022; Zhao et al., 2022). Yet, which metabolites are of greater importance for berberine’s action as well as the metabolic origin of these metabolites remain unclear.

Intestinal metabolism seems to play a major role in the generation of bioactive metabolites derived from berberine. (Liu et al., 2010) reported that the concentration of berberine in rat plasma was higher after intraportal administration than after intraduodenal administration, suggesting that the first-pass elimination of berberine occurs predominantly in the small intestine rather than in the liver. Whether the biotransformation of berberine is mainly performed by the host epithelial cells or the gut microbiota is not yet clear. Although berberrubine and thalifendine are often cited as the most abundant metabolites in feces (Ma et al., 2013; Alolga et al., 2016; Feng et al., 2021), until now, only two fecal metabolites, dihydroberberine and oxyberberine, have been described as gut microbiota-derived metabolites. No specific bacterial species able to produce these metabolites from berberine have been conclusively identified (Feng et al., 2015; Li et al., 2020) (Figure 1B). Dihydroberberine results from the reduction of berberine by bacterial nitroreductases, which is then absorbed into intestinal tissue, and is subsequently oxidised back to berberine to enter the blood stream (Feng et al., 2015). Higher levels of nitroreductase activity in the gut microbiota increased levels of intestinal dihydroberberine and increased berberine oral bioavailability in rats and humans, which was associated with berberine-mediated improvement of lipid metabolism (Wang et al., 2017c). Oxyberberine results from oxidation by the gut microbiota, and both metabolites have shown anti-inflammatory and hypoglycemic effects in animal models, where the effect of oxyberberine was superior to that of berberine (Turner et al., 2008; de Maesschalck et al., 2015; Li et al., 2019; Li et al., 2020; Dou et al., 2021). The amount of these metabolites was reduced in pseudo germ-free animals, confirming the role of the gut microbiota in their formation (Feng et al., 2015; Li et al., 2020).

Studying berberine-derived metabolites and gut bacterial species that produce these is of great significance in the exploration of the pharmacological mechanisms of berberine, however, little information is present at this day. This work aimed therefore to bring further evidence on berberine-gut microbiota bidirectional interactions in broiler chickens. First, we studied the *in vivo* effect of different doses of in-feed berberine on the intestinal microbiota composition and intestinal health, as well as the disposition of berberine metabolites in plasma and intestines. Secondly, we investigated the mechanism of formation of these metabolites in the intestine, by studying the contribution of the host and the gut microbiota in berberine intestinal metabolism, combining *in vitro* and *in vivo* studies.

## Material and methods

### Chemicals and reagents

Berberine chloride and palmatine chloride (both purity ≥98%) were obtained from Merck (Overijse, Belgium). Demethyleneberberine (purity >98%), columbamine (purity >98%) and dihydroberberine (purity >95%), were purchased at AbMole BioScience (Brussels, Belgium). Jatrorrhizine chloride (purity >95%), berberrubine chloride (purity >95%) and oxyberberine (purity >95%) were obtained from Toronto Research Chemicals (North York, Canada). Berberine hydrochloride-d6 (Toronto Research Chemicals) and D-tetrahydropalmatine (Sigma-Aldrich) were used as internal standards. Stock solutions of berberine, palmatine, and berberine-d6 were prepared at 1 mg/mL in methanol. Stock solutions of all other compounds were prepared at 100 μg/mL in a mixture of DMSO/methanol (1/9, v/v). Stock solutions were stored at ≤ −20°C. Working solutions were prepared by dilution of the stock solutions in Milli-Q grade water. For stability reasons, for dihydroberberine only, working solutions were prepared in Milli-Q water containing 17 mg/mL L-ascorbic acid. Working solutions were stored at 2–8°C. Solvents involved in stock solution preparation and/or sample extraction, methanol and acetonitrile, were both of HPLC grade (Fisher Scientific, Filter Service, Eupen, Belgium). Acetonitrile as the organic mobile phase component was of ULC/MS grade (Biosolve, Valkenswaard, the Netherlands). Formic acid, used in sample extraction as well as in mobile phase preparation, was also of ULC/MS grade (Biosolve). Dimethylsulfoxide (DMSO) (purity ≥99.5%) used in preparation of stock solutions, and L-ascorbic acid (purity ≥98%) used in preparation of working solutions and in sample preparation as well, were both from Sigma Aldrich. Water of Milli-Q grade used for preparation of the aqueous mobile phase component, and other solutions as well, was produced in-house by a water purifying system Milli-Q-SP (Sigma-Aldrich). For *in vitro* work, a 1 mg/mL berberine stock solution in deionized water was used, except otherwise specified.

### Animals, experimental design and dietary treatment

The study was undertaken following the guidelines of the ethics committee of the Faculty of Veterinary Medicine and Bioscience Engineering, Ghent University, in accordance with the EU Directive 2010/63/EU. One-day-old Ross 308 male broilers were obtained from a local hatchery and housed in pens on wood shavings. They were allotted into 4 groups, with three replicate pens per group each housing 8 chickens. The animals were not vaccinated. Water and commercial starter feed (day 1–12, FARM 1 Mash, Country’s Best, Versele Laga, Deinze, Belgium) or grower (day 13–21, FARM 2 mash, Country’s Best, Versele Laga, Deinze, Belgium) were provided *ad libitum*. The control group received the standard non-supplemented diets, whereas the chickens in other groups were fed the same feed supplemented with 0.1, 0.5 or 1 g berberine/kg feed throughout the whole trial period. At day 21, 4 birds per pen were weighted then euthanized by sodium pentobarbital injection for sampling. Individual jejunal, ileal, caecal and colonic contents were stored at −20°C for microbiota composition analysis (16S rRNA gene sequencing). Additionally, ileal and caecal content samples were collected and stored at −20°C for berberine and berberine-derived metabolite quantification. Duodenal tissue samples were collected and fixed in 4% phosphate buffered formaldehyde for histological analysis. Blood samples were collected in heparin tubes and centrifuged at 524 *g* for 10 min to recover the plasma fraction, which was further mixed with 17 mg/mL L-ascorbic acid for dihydroberberine stabilization, and stored at −20°C for berberine and berberine-derived metabolite quantification.

### DNA extraction from intestinal content

DNA was extracted from intestinal content using the CTAB method as previously described with minor modifications (Aguirre et al., 2019). In brief, 200 mg of jejunal or ileal content or 100 mg of caecal or colonic content was suspended in 500 µL CTAB buffer (hexade-cyltrimethylammonium bromide >98% (Sigma Aldrich) 5% (w/v), 0.35 M NaCl, 120 nM K2HPO4) and 500 µL phenol–chloroform-isoamyl alcohol (25:24:1). The mixture was homogenized by grinding (2×) with 500 mg unwashed glass beads (Sigma-Aldrich) in a bead beater (2 min, 30 Hz for the ileal content, 1.5 min, 22.5 Hz for the other segments; TissueLyser; Qiagen, Hilden, Germany) with a 30 s interval between shakings. Samples were centrifuged for 10 min at 6,010 g and 300 μL of the supernatant was transferred to a new tube. A second extraction from the remaining content was performed by adding 250 µL CTAB buffer and homogenizing and centrifuging the sample as described above. An equal volume (600 μL) of chloroform-isoamyl alcohol (24:1) was added to the supernatant collected in order to remove the phenol from the samples. The mixture was further centrifuged at 16,000 *g* for 10 s. 500 μL of the aqueous phase were transferred to a new tube. Nucleic acids were precipitated with 2 volumes of PEG-6000 solution (polyethylene glycol 30% w/v; 1.6 M NaCl) for 2 h at room temperature. Samples were centrifuged (13,000 g, 20 min) and washed with 1 mL ice-cold ethanol (70% v/v). The pellet obtained was further centrifuged (13,000 g, 20 min), dried and resuspended in 100 μL de-ionized water (LiChrosolv Water, Merck, Darmstadt, Germany). The quality and the concentration of the DNA was examined spectrophotometrically (NanoDrop, Thermo Scientific, Waltham, MA, United States).

### 16S rRNA gene sequencing for microbiota composition analysis

To characterize the taxonomic groups in the ileal and caecal microbiota of the chickens, the V3-V4 hypervariable region of 16s rRNA gene was amplified using the gene-specific primers S-D-Bact-0341-b-S-17 (5′-TCGTCG GCA GCG TCA GAT GTG TAT AAG AGA CAG CCTACGGGNGGC WGC AG-3′) and S-D-Bact-0785-a-A-21 (5′-GTC TCG TGG GCT CGG AGA TGT GTA TAA GAGACA GGA CTACHVGGG TAT CTA ATC C-3′) (Klindworth et al., 2013). Each 25 μL PCR reaction contained 2.5 μL of DNA (∼2 ng/μL), 0.2 μM of each of the primers and 12.5 μL 2 × KAPA HiFi HotStart ReadyMix (Kapa Biosystems, Wilmington, MA, United States). The program of PCR was set as follows: initial denaturation at 95°C for 3 min, followed by 25 cycles of 95°C for 30 s, 55°C for 30 s, 72°C for 30 s and a final extension at 72°C for 5 min. The PCR products were purified using CleanNGS beads (CleanNA, Waddinxveen, Netherlands). The DNA quantity and quality were analyzed spectrophotometrically (NanoDrop) and by agarose gel electrophoresis. A second PCR step was used to attach dual indices and Illumina sequencing adapters (i5 and i7 primers) to the 16S V3-V4 fragment in a 50 μL reaction volume containing 5 μL of purified PCR product, 25 µL of 2× KAPA HiFi HotStart ReadyMix and 0.5 μM of primers. The PCR conditions were the same as the first PCR with the number of cycles reduced to 8. The final PCR products were purified using the same method as above and the concentration was determined using the Quantus double-stranded DNA assay (Promega, Madison, WI, United States). The final bar-coded libraries were combined to an equimolar 5 nM pool and sequenced using Illumina MiSeq v3 technology (2 × 300 bp, paired-end) at Macrogen (Gasan-dong, World Meridian I, Seoul, Korea).

Demultiplexing of the amplicon dataset and deletion of the barcodes was done by the sequencing provider. Quality of the raw sequence data was checked with the FastQC quality-control tool (Babraham Bioinformatics, Cambridge, United Kingdom) followed by initial quality filtering using Trimmomatic v0.38 by cutting reads with an average quality per base below 15 using a 4-base sliding window and discarding reads with a minimum length of 200 bp (Bolger et al., 2014). The paired-end sequences were assembled, and primers were removed using PANDAseq (Masella et al., 2012), with a quality threshold of 0.9 and length cut-off values for the merged sequences between 390 and 430 bp. Chimeric sequences were removed using UCHIME (Edgar et al., 2011). Open-reference operational taxonomic unit (OTU) picking was performed at 97% sequence similarity using USEARCH (v6.1) and converted to an OTU table (Edgar, 2010). OTU taxonomy was assigned against the Silva database (v128, clustered at 97% identity) (Quast et al., 2013) using the PyNast algorithm with QIIME (v1.9.1) default parameters (Caporaso et al., 2010). OTUs with a total abundance below 0.01% of the total sequences were discarded (Bokulich et al., 2013), resulting in an average of approximately 43296.511 reads per sample. Samples with insufficient sequencing depth (<5,000 reads) were excluded from the dataset. 11 to 12 samples were left for the analysis per concentration and per intestinal segment.

### Metabolic function prediction of the microbial communities

To gain more insight into the effect of the different doses of berberine on the possible functional pathways of the microbial communities, the functional composition was predicted using PICRUSt (Phylogenetic Investigation of Communities by Reconstruction of Unobserved States) (Langille et al., 2013). PICRUSt uses precomputed ancestral state reconstructions based on the Greengenes database. Therefore, OTU picking was reperformed as described above with following modifications: closed-reference OTU picking was used, and OTU taxonomy was assigned against the Greengenes database (v13.5) (DeSantis et al., 2006) after which the OTU counts were normalized by their expected 16S copy number using QIIME (Kembel et al., 2012; Angly et al., 2014). Metagenome predictions were performed against the KEGG database (Kyoto Encyclopedia of Genes and Genomes (Kanehisa and Goto, 2000)). The resulting KEGG orthologs (KOs) were further summarized into functional modules based on the Gut-specific Metabolic Modules (GMM) database using GoMixer (Raes Lab). The contribution of bacterial OTUs to KOs related to methyltransferase activity in the Wood-Ljundahl pathway (K00198, K14138, K00197, K00194) was computed with the script “metagenome_contributions.py”.

### Berberine metabolism in gut microbiota cultures

In a first set-up, content from the jejunum, ileum, caecum and colon content from two 3-week-old broiler chickens were sampled and pooled, in anaerobic conditions. 1:100 diluted jejunal content and 1:1,000 diluted ileal, caecal and colonic content were incubated in M2GSC medium (Barcenilla et al., 2000), adjusted to pH 6.3, 6.9, 6.0 and 7.5 respectively, containing 10 μg/mL berberine, in a 24-well plate, for 48 h. This concentration has been confirmed not to affect the growth of strict or facultative anaerobes (Dehau et al., 2023). 200 μL of cultures were collected at 0 h and 48 h post-incubation, then centrifuged at 13,000 *g* for 10 min 180 μL of supernatants were mixed with 20 µL of L-ascorbic acid 170 mg/mL solution in water for dihydroberberine stabilization and stored at −20°C until metabolite analysis. All experiments were performed in triplicate at 37°C–40°C under anaerobic conditions.

In a second set-up, caecal content from a 4-week-old broiler chicken was sampled, in anaerobic conditions. Different dilutions of caecal content (10^−3^, 10^−4^, 10^−5^, 10^−6^, 10^−7^, 10^−8^) were incubated in M2GSC medium adjusted to pH 6, either supplemented with 10 μg/mL berberine (BBR) or not supplemented (Control), in a 24-well plate, for 48 h. Cultures were collected and processed as described before. Pellets were stored at −20°C until RNA extraction. All experiments were performed in triplicate at 37°C–40°C under anaerobic conditions to mimic the physiological conditions of the broiler caecum. Metabolite production was expressed as a log_2_ fold change, calculated as the log_2_ of the ratio of the metabolite response at t = 48 h over the metabolite response at t = 0 h.

### RNA extraction and cDNA synthesis

The AllPrep PowerFecal DNA/RNA Kit (Qiagen, Hilden, Germany) was used for simultaneous RNA and DNA extraction from the pellets of the gut microbiota cultures. Extraction was performed according to the recommendations of the manufacturer, including the additional steps to increase yield. RNA extracts were subjected to DNase treatment with the Turbo DNA-free Kit for removal of residual DNA (Invitrogen, Vilnius, Lithuania). The RNA and DNA quantity and quality were analysed spectrophotometrically (NanoDrop). The RNA was subsequently converted to cDNA using the iScript cDNA Synthesis Kit (Biorad, Hercules, United States). To characterize the taxonomic composition in the total (DNA) and active (cDNA) community, the V3-V4 hypervariable region of 16s rRNA gene was amplified and sequenced as described before.

### Berberine metabolism in gut microbiota cultures enriched with single bacteria

Six strains were tested for their ability to convert berberine into berberine-demethylated metabolites. Selection was based on Maaslin2 associations, showing that OTUs belonging to the genus *Blautia* and *Anaerostipes* were correlated with berberrubine or thalifendine, and availability in-house, including *B. coccoides* (DSM 935)*, Blautia hansenii* (DSM 20583)*, Blautia hydrogenotrophica* (DSM 10507), *Blautia luti* (DSM 14534) and *Anaerostipes butyraticus* (35–7 (Eeckhaut et al., 2011))*. Eubacterium limosum* (LMG 28910) was also included as it previously showed to carry O-demethylation (Possemiers et al., 2008; Rich et al., 2022), a reaction involved in the transformation of berberine to berberrubine or thalifendine. Strains were sub-cultured overnight in M2GSC medium pH 6. The next day, caecal content from a 3-week-old broiler chicken was sampled in anaerobic conditions and 1:1,000 diluted in M2GSC medium pH 6 containing 10 μg/mL berberine. Caecal mixtures were enriched with 1:100 diluted overnight cultures of single bacteria, or with M2GSC (control), and incubated in a 24-well plate, for 48 h. Preliminary work showed that the growth of these bacteria was not significantly affected by the used concentration of berberine (Supplementary Figure S1). In addition, as caecal microbiota supplemented with *Blautia coccoides* showed to efficiently metabolize berberine, the metabolic profile of *B. coccoides* alone was further assessed: 1:250 diluted overnight cultures of *B. coccoides* were incubated in M2GSC medium pH 6 containing 10 µg berberine/mL for 48 h. Samples were collected and processed as described before. Each bacterial strain was tested in triplicate at 37°C–40°C under anaerobic conditions.

### Short-chain fatty acid quantification

#### In caecal microbiota cultures supplemented with berberine or berberrubine

As berberine and its demethylated metabolites correlated with SCFAs *in vivo*, their ability to directly influence SCFA metabolism was assessed *in vitro*. Only berberine and berberrubine could be tested as thalifendine was not commercially available. 5 mM solutions of berberine or berberrubine were prepared in 1:10 DMSO:methanol and stored at −20°C until use. Caecal content from a 4-week-old broiler was sampled in anaerobic conditions. 1:100 diluted caecal content was incubated with M2GSC medium adjusted to pH 6, either supplemented with berberine (25, 2.5 µM or 9.3, 0.9 μg/mL), berberrubine (2.5, 0.25 µM or 0.9, 0.09 μg/mL) or 1:10 DMSO/Methanol (1:2,000 diluted, 1:20,000 diluted, controls), in 15-mL Eppendorf tubes, for 24 h. Cultures were centrifuged at 3,724 *g* for 5 min 1 mL 1:2 diluted supernatant in Milli-Q water was used for SCFA analysis.

#### In caecal contents from chickens fed a control or a berberine-supplemented diet

250 mg caecal content derived from the *in vivo* trial were weighed and diluted 1:20 for SCFA analysis.

Acetate, propionate and butyrate were quantified using the GC-FID method described by (De Weirdt et al., 2010). This method involves the extraction of SCFA from samples with diethyl ether after the addition of 2-methyl hexanoic acid as an internal standard.

### Cell viability assay

Caco-2 cells (human intestinal epithelial cells) were cultured in MEM (Gibco, Paisley, United Kingdom) supplemented with 10% Fetal Bovine Serum (FBS), 1% L-glutamine (Gibco), 1% MEM NEAA (Gibco) and 1% penicillin/streptomycin (P/S) (Gibco). T84 cells (human intestinal epithelial cells) were cultured in 1:1 DMEM/F12 (Gibco) supplemented with 10% FBS, 1% L-glutamine and 1% P/S (37°C, 5% CO_2_). The neutral red uptake assay was employed to evaluate the potential cytotoxicity of berberine (Repetto et al., 2008). Briefly, Caco-2 and T84 were seeded at 2 × 10^5^ cells/well and 5 × 10^4^ cells/well respectively in 96-well plates (Greiner Bio-one, Frickenhausen, Germany) and cultivated for 24 h in antibiotic-free medium (treatment medium) for attachment. Medium was removed and cells were treated with a 2-fold dilution of berberine in treatment medium, ranging from 100 μg/mL to 0.78 μg/mL, for 48 h. A 10-fold dilution of sodium dodecyl sulfate (Sigma-Aldrich), ranging from 10 mg/mL to 10^−6^ mg/mL was used a positive control. Cells were washed with 200 µL pre-warmed HBSS(+) (Gibco) and then treated with 150 µL of Neutral Red medium, consisting of 1:80 Neutral Red solution 0.33% (Sigma-Aldrich)/treatment medium for 3 h. Cells were washed in 200 µL HBSS(+) and treated with 100 µL of Neutral Red Desorb solution, consisting of 1% glacial acetic acid (Merck, Darmstadt, Germany), 50% ethanol (95%, Chem-Lab, Zedelgem, Belgium) and 49% deionized water. Plates were shaken in darkness for 20 min and absorbance was measured at 540 nm to evaluate Neutral Red extraction. All measurements were performed in 5 replicates.

### Berberine metabolism in intestinal cell cultures

Caco-2 and T84 cells were seeded at 2 × 10^5^ cells/well in 96-well plates and cultivated for 24 h in their respective medium, as described before. Cells were treated with 200 µL of 0.1 μg/mL berberine in antibiotic-free medium and incubated for 48 h. Berberine medium only was incubated in the same conditions as the cells and used as control for metabolite baseline levels. 100 μL of cell cultures or control medium were collected and centrifuged at 13,000 *g* for 10 min 80 μL of the supernatant was mixed with 20 µL 170 mg/mL L-ascorbic acid solution in water for dihydroberberine stabilization and stored at −20°C until metabolite analysis. All experiments were performed in triplicate at 37°C, 5% CO_2_. Metabolite production was expressed as a log_2_ fold change, calculated as the log_2_ of the ratio of the metabolite response at t = 48 h in the cell cultures over the metabolite response at t = 48 h in the control medium.

### Quantification of berberine and berberine-derived metabolites

#### UPLC-ESI-MS/MS analysis

The UPLC instrument consisted of an Acquity H-Class Quaternary Solvent Manager and an Acquity FTN Sample Manager (Waters, Berchem, Belgium). For chromatographic separation, an Acquity UPLC^®^ HSS T3 column (1.8 µm, 100 mm × 2.1 mm i.d.) was used, protected by a precolumn of the same type (VanGuardTM, 5 mm × 2.1 mm i.d.), both from Waters. A gradient elution was performed with a mobile phase of 0.1% (v/v) formic acid in water (A) and acetonitrile (B), at a flow rate of 0.3 mL/min, i.e. 0 min, 82.5% A/17.5% B; 0–6.5 min, to 65.0% A/35% B; 6.5–6.6 min, to 10% A/90% B; 6.6–10.9 min, 10% A/90% B, 10.9–11.0 min, to 82.5% A/17.5% B; 11–15 min, 82.5% A/17.5% B, with the flow rate kept constant at 0.3 mL/min. The column temperature was maintained at 25°C. The autosampler temperature was set at 10°C. The UPLC effluent was sent from 1.2 min to 9.5 min by use of a divert valve to a Xevo TQ-S^®^ triple quadrupole mass spectrometer from Water, equipped with an ESI ion source operating in the positive ionization mode from. The UPLC-MS/MS analysis was run under control of MassLynx software (v4.1), which was used for subsequent data processing as well. For the injection of 1/5 diluted intestinal content samples (see section Preparation of intestinal content samples (ileum and caecum)), the divert valve was used in addition from 7.1 min to 8.4 min to deviate the high amount of berberine and associated IS berberine-d6 eluting in that time window from the UPLC column directly to the waste, rather than to the MS detector to avoid oversaturation of the instrument, influencing subsequent sample extract injections at well. Operating conditions for the ESI source used in the positive ionization mode were optimized by direct infusion of all individual components in combination with the mobile phase at 50% A/50% B delivered at a flow rate of 0.3 mL/min. The following tune parameters were used for detection of all components: capillary voltage, 3 kV, cone voltage, 10 V, source offset, 50 V, source temperature, 150°C, desolvation temperature, 500°C, desolvation gas flow, 800 L/h, cone gas flow, 150 L/h, collision gas flow, 0.2 mL/min, ion energy 1 and 2, 0.5, LM 1 and LM 2 resolution, 3, HM 1 and HM 2 resolution, 15. Components were detected in MS/MS mode using component specific MRM (Multiple Reaction Monitoring) transitions (Supplementary Table S1). Since a commercial standard of thalifendine was not available, MS/MS detection parameters and calibration of berberrubine were used for the detection and quantification of thalifendine, which is a reasonable approach, since both compounds are isomers. No commercial reference standards were available for phase II metabolites, therefore “calculated” MRM transitions were defined as follows: [M + -Gluc] > M+ for glucuronide forms and [M + -SO_3_] > M+ for sulfate forms, where the loss of glucuronic acid or sulfate corresponded to a mass shift of 176 Da or 80 Da respectively. Phase II metabolites were quantified relatively using the berberine calibration curve (Wang et al., 2017a; Kwon et al., 2020). Since both demethyleneberberine and demethylated jatrorrhizine and columbamine have the same molecular mass (m/z at 324) and each compound has 2 OH-groups available for glucuronidation or sulfation, in total 6 possible glucuronide or 6 sulfate metabolites were included in the UPLC-MS/MS analysis. For practical reasons, the name was mentioned as demethyleneberberine-glucuronide_01/_02/_03/_04/_05/_06 or demethyleneberberine-sulfate_01/_02/_03/_04/_05/_06. Stock solution of berberine was preliminary examined by UPLC-MS/MS and impurities were identified as jatrorrhizine, palmatine (<0.5%), columbamine, berberrubine (<0.2%), demethyleneberberine (<0.1%) and oxyberberine (<0.001%).

#### Preparation of plasma samples

A 137.5 µL plasma sample containing 17 mg/mL L-ascorbic acid was used for sample extraction. The ascorbic acid solution was added to prevent oxidation of dihydroberberine to berberine during sample storage (Feng et al., 2015). The plasma sample was transferred to an OstroTM 96 well-plate (25 mg) (Waters). Subsequently, 50 µL Milli-Q water and 25 µL of an internal standard solution containing 100 ng/mL of both berberine-d6 and tetrahydropalmatine were added. A 640 µL volume of 1% (v/v) formic acid solution in acetonitrile (1/3 ratio) was added, followed by gentle mixing. Vacuum was applied on the Ostro™ plate for 5 min and the eluate was collected in a 2 mL square 96-well collector plate. The sample was transferred in a glass tube and evaporated at 40°C under a gentle stream of nitrogen in a Pierce (Rockford, United States) Reacti-Therm III™ Heating Module and Reacti-Vap™ III Module. The dried sample extract was reconstituted in 500 µL Milli-Q water, followed by injection of a 5 µL sample aliquot onto the UPLC-MS/MS apparatus. Quantification was based on the ratio of the analyte peak area compared to the peak area of the internal standard berberine-d6.

#### Preparation of calibrators and quality controls–plasma

Calibration standards and quality control samples were prepared as follows: 137.5 µL of blank plasma stabilized with 17 mg/mL L-ascorbic acid were supplemented with 25 µL mixed working solutions of berberine and metabolites (except dihydroberberine) at different levels, 25 µL of dihydroberberine working solution, stabilized with 17 mg/mL L-ascorbic acid, at different levels and 25 µL of an internal standard solution containing 100 ng/mL of both berberine-d6 and tetrahydropalmatine. The calibration curve was in the 0.1–100 ng/mL range, including 0.1, 0.5, 1, 2.5, 5, 10, 25, 25, 50 and 100 ng/mL levels.

#### Preparation of intestinal content samples (ileum and caecum)

250 mg of ileal or caecal content was weighed in a 15 mL polypropylene centrifuge tube and 250 µL of ascorbic acid 170 mg/mL solution was added for stabilization of dihydroberberine, followed by brief vortexing. Subsequently, 25 µL of an internal standard solution containing 250 μg/mL of berberine-d6 and 2.5 μg/mL tetrahydropalmatine were added, followed by brief vortexing. A 2.5 mL volume of extraction solvent was then added, consisting of a 1% (v/v) formic acid solution in methanol. The sample was put on a rotary shaker set at 80 rpm for 20 min, followed by centrifugation (3,720 g, 10 min, 4°C). The supernatant was then diluted, by a factor 5 for measurement of low abundance components, and by a factor 2,000 for measurement of high abundance components. The 1/5 diluted sample extract was prepared directly in an autosampler vial as follows: 200 µL sample extract were mixed with 100 µL ascorbic acid 170 mg/mL solution and 700 µL Milli-Q water. For the 1/2,000 diluted sample extract, an intermediate 1/100 sample dilution was prepared by combining 500 µL sample extract and 49.5 mL of Milli-Q water. 50 μL was then transferred in an autosampler vial followed by the addition of 950 µL of Milli-Q water. For both dilutions, a 5 µL sample aliquot was injected onto the UPLC-MS/MS apparatus. Quantification was based on the ratio of the analyte peak area compared to the peak area of the internal standard tetrahydropalmatine for 1/5 diluted sample extracts, and on the ratio of the analyte peak area compared to the peak area of the internal standard berberine-d6 for 1/2,000 diluted sample extracts.

#### Preparation of calibrators and quality controls–intestinal content

Calibration standards and quality control samples were prepared as follows: 250 mg of blank ileal or caecal content were supplemented with 250 µL of ascorbic acid 170 mg/mL solution, 25 µL mixed solution of berberine and metabolites (with the exception of dihydroberberine) at different levels, 25 µL of dihydroberberine solution, stabilized with 17 mg/mL ascorbic acid, at different levels and 25 µL of an internal standard solution containing 0.625 μg/mL of berberine-d6 and 2.5 μg/mL tetrahydropalmatine. The calibration curve was in the 62.5–5,000 ng/g range, and included 62.5, 125, 250, 500, 1,000, 2,500, and 5,000 ng/g levels.

#### Method validation

The UPLC-MS/MS analysis method for the quantification of berberine and berberine-derived metabolites both in plasma and ileal/caecal intestinal content was validated to the guidelines of the European Medicines Agency, i.e., of the Committee for Veterinary Medicinal Products (EMEA/CVMP/VICH/463202/2009) and of the Committee for Medicinal Products for Human Use (EMEA/CHMP/EWP/192217/20), and in accordance with the EU Directive 2002/657/EC. The following parameters were evaluated: linearity, within-day and between-day precision and accuracy, carry-over, specificity and analyte stability in processed sample extract. The results of the method validation can be found in Supplementary Method S1. Limits of quantification (LOQ) were 0.1 ng/mL for plasma and 62.5 ng/g for intestinal content for all compounds.

#### Preparation of gut microbiota/cell culture samples

The sample preparation procedure used for the analysis of berberine and associated metabolites in cultures with intestinal microbiota or epithelial cells was like the one used for plasma samples. 75 μL of supernatant stabilized with 17 mg/mL L-ascorbic acid was used for extraction. The analysis of berberine and berberine-derived metabolites in these samples was semiquantitative, i.e., analyte responses (peak area analyte/peak area internal standard) were compared between the different experimental conditions. For that reason, no full method validation was performed with these matrices.

### Intestinal morphology

24 h formalin-fixed duodenal intestinal tissue segments were embedded in paraffin wax and sectioned at 5 μm, followed by haematoxylin and eosin staining. Villus length and crypt depth were assessed using a PC-based image analysis system (Leica Application Suite V4, LAS V4.; Leica, Diegem Belgium). Measurements were performed on at least 9 randomly selected villi/crypt apparently not exhibiting mechanical damage, when present, after which the average per animal was calculated.

### CD3 immunohistochemistry

Slides for immunohistochemical staining for CD3^+^ T-cells were automatically deparaffinized (Shandon Varistain-Gemini) before antigen retrieval with a pressure cooker in citrate buffer (10 mM, pH 6). Endogenous peroxidase activity was blocked by treating the slides with peroxidase blocking reagent (S2023, Dako, Glostrup, Denmark) for 5 min. The presence of T-cells (CD3-positive cell abundance) in duodenal tissue from 21-day old chickens was evaluated using polyclonal primary antibodies against CD3 (A0452, Dako, 1:100 dilution, 30 min at room temperature), followed by incubation with a secondary labelled polymer-HRP anti-rabbit antibody (Envision + System-HRP (DAB) (K4011), 30 min at room temperature). Slides were evaluated using the computer-based image analysis program, LAS V4.1. The CD3^+^ area percentage in the duodenal tissue was quantified using four representative fields of view per intestinal section (10x objective).

### Statistical analysis

Statistical analyses of the *in vivo* chicken’s phenotypes and 16S data were performed using R (v3.6.1). In all analyses, the factor berberine concentration was set as a covariate and the factor pen was set as a random factor or a covariate in the model, as appropriate. Metabolite and histological data were analysed using a linear mixed model, using the lmer function from the lme4 package (v1.1-26). Alpha diversity was measured based on the observed OTUs (or observed KOs for the functional data), Chao1 and Shannon diversity index using the phyloseq pipeline (v1.30.0) (McMurdie and Holmes, 2013). Differences in alpha diversity were assessed using a linear mixed model like before. Beta diversity was calculated using Bray–Curtis and unweighted Unifrac distances. The significant differences in the community composition between the groups were determined through a permutational multivariate analysis of variance using distance matrices (PERMANOVA), using the adonis function of the vegan package (v2.5-7). In case a significant effect of the berberine supplementation was observed, the pairwise comparison between the group levels was performed using the function pairwise.perm.manova from the RVAideMemoire package (v0.9-81-2, R v4.1.2) and the Bonferroni corrected *p*-values were reported. To detect differentially abundant taxa, LinDA (v0.1.0) was applied on the centered log-ratio transformed community composition data. For all tests, an adjusted *p*-value (q-value) ≤ 0.05 was considered significant. The associations of microbial abundances at OTU level with berberine metabolites measured in the intestinal content were analyzed using the multivariate analysis by linear models (MaAsLin2) R package. MaAsLin2 analysis was performed on caecal samples, only from animals supplemented with 0.1 or 0.5 g berberine/kg feed. Significant associations were plotted for confirmation using Spearman correlation with the ggscatter() function of the ggpubr R package (v0.4.0).

Statistical analyses of the *in vitro* 16S data were also performed using R (v3.6.1). A new categorical variable was created, combining treatment (Control, BBR) and DNA type (DNA, cDNA). To detect differentially abundant active taxa between the BBRcDNA and the ControlcDNA group, DESeq2 (v1.26.0) was applied with the ControlcDNA group set as reference level. The likelihood ratio test and the dilution variable as reduced model were used as arguments in the DESeq() function. Significant differences were obtained using a Wald test followed by a Benjamini–Hochberg multiple hypothesis correction. For all tests, an adjusted *p*-value (q-value) ≤ 0.05 was considered significant. The association between abundances of active OTUs and thalifendine or berberrubine measured in the caecal microbiota cultures were analyzed using MaAsLin2 like before. MaAsLin2 analysis was performed on the BBRcDNA group, for dilution 3 to 6 where the metabolic activity was the highest, while controlling for berberine measured concentration (fixed effect) and dilution (random effect) covariates.

Neutral Red Uptake assay results were fitted with a Hill’s model using the GraphPad Prism software (version 8.4.3, San Diego CA, California). Biologically relevant metabolite production in bacterial or cell cultures *in vitro* was identified as an absolute fold change >4 as compared to the control.

## Results

### Medium and high concentrations of in-feed berberine tend to improve villus length in the duodenum

No significant difference in bodyweight was observed between groups (Table 1). Villus height, crypt depth and villus to crypt ratios were measured in the duodenum of chickens fed either a control diet or a diet supplemented with a low (0.1 g/kg feed), medium (0.5 g/kg feed) or high level (1 g/kg feed) of berberine for 21-day, to evaluate the dose effect of in-feed berberine on intestinal health. None of the different concentrations of in-feed berberine impacted the crypt depth nor the crypt-to-villus ratio in the duodenum. The medium and high concentrations tended to increase the length of villi in the duodenum (0.5: *p* = 0.091, 1: *p* = 0.080) (Table 1).

**TABLE 1:** Effect of different concentrations of berberine in diet (0, 0.1, 0.5 or 1 g/kg feed) on bodyweight, intestinal morphology and T-cell abundance in the duodenum of chickens 21 days post-hatch (LMM). Data represent the mean ± standard deviation (n = 12). Analysis is based on at least 9 measurements per section for villus height and crypt depth analysis and 4 microscopic fields of view per section for CD3 measurements.

	Berberine concentration in the feed (g/kg feed)					*p*-value		
	0	0.1	0.5	1		0.1 vs. 0	0.5 vs. 0	1 vs. 0
Bodyweight (g)	613 ± 75	655 ± 76	703 ± 115	671 ± 55		0.397	0.170	0.652
Villus length (µm)	1,691 ± 129	1,678 ± 101	1,815 ± 148	1,820 ± 150		0.844	0.091	0.080
Crypt depth (µm)	116 ± 17	114 ± 21	104 ± 15	103 ± 17		0.870	0.382	0.397
Villus to crypt ratio	14.97 ± 2.00	15.30 ± 2.50	17.98 ± 2.53	18.31 ± 3.21		0.864	0.138	0.119
CD3^+^ area percentage	7.45 ± 1.99	8.39 ± 1.05	8.89 ± 2.09	7.11 ± 2.18		0.442	0.258	0.780

The amount of CD3^+^ T-cells was determined in the duodenal tissue as a marker for intestinal inflammation, but no effect was observed for any of the berberine concentrations in-feed as compared to the control (Table 1).

### Lower concentrations of berberine modulate the gut microbiota

**FIGURE 2:**
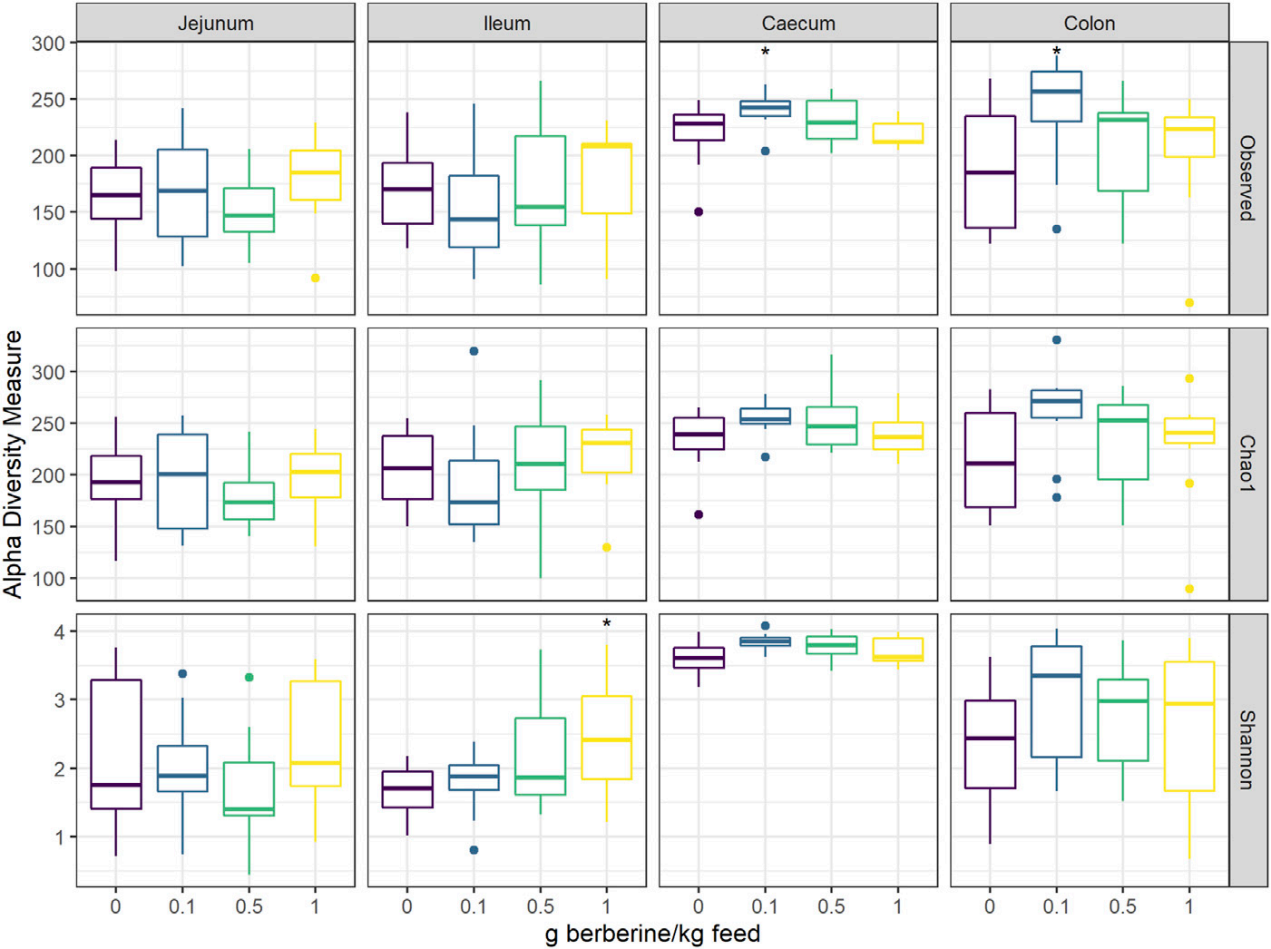
Alpha diversity of the microbial community in different intestinal segments, from chickens fed a diet supplemented with 0, 0.1, 0.5 or 1 g berberine/kg feed for 21 days (n = 12). Observed: observed OTUs, Chao1: estimated species richness and Shannon: estimated species diversity. *: *p* ≤ 0.05.1 g berberine/kg feed significantly increased the Shannon index in the ileum (*p* = 0.045). 0.5 g berberine/kg feed tended to increase the bacterial richness in the caecum (Observed number of OTUs, *p* = 0.087). 0.1 g berberine/kg feed significantly increased the richness in the caecum (Observed number of OTUs, *p* = 0.041) and the colon (Observed number of OTUs, *p* = 0.026), and tended to increase the estimated richness in the caecum (Chao1 estimator, *p* = 0.087).

To investigate the effect of different concentrations of berberine on the microbial composition, the microbiota from the jejunum, ileum, caecum, and colon of broilers from all groups was analysed by 16S rRNA gene sequencing. Alpha diversity, including the observed number of OTUs, the estimated OTUs richness (Chao1) and the estimated community diversity (Shannon index) was measured to detect the richness and diversity of microbial communities within the samples of each group (Figure 2). None of the different doses of berberine impacted the alpha-diversity in the jejunum. In the ileum, the highest concentration of berberine significantly increased the estimated microbial diversity (Shannon index, *p* = 0.045). The middle concentration of berberine only tended to increase the bacterial richness in the caecum (Observed number of OTUs, *p* = 0.087). The lowest concentration of berberine significantly increased the observed number of OTUs in the caecum (*p* = 0.041) and in the colon (*p* = 0.026) compared to the control group.

Bray–Curtis and unweighted UniFrac dissimilarities were used to investigate the effect of the supplementation with increasing doses of berberine in the diet on the community structure of the jejunal, ileal, caecal or colonic microbiota. Bray–Curtis accounts for relative abundances of taxa while unweighted UniFrac uses a presence/absence metric, which gives information about rare species, that are possibly omitted by abundance-based methods, and incorporates phylogenetic information, which is thought to improve microbial diversity estimation. Berberine had a major effect on the overall microbial community composition in all intestinal segments, except in the jejunum when sample dissimilarity was measured by the Bray Curtis index (Supplementary Table S2). The pairwise PERMANOVA analysis revealed that in the jejunum, only the lowest dose of berberine tended to alter the overall microbial community composition (Supplementary Table S3). The ileal and colonic microbial communities were similarly affected by the lowest and highest dose of berberine: both had an effect in the UniFrac distance while only the highest dose induced changes in the microbiota structure in the Bray-Curtis distance. In the caecum, the three levels of berberine supplementation significantly shifted the overall microbial community structure (Supplementary Table S3).

As the different doses of in-feed berberine resulted in a significant shift in the microbial community structure, we further focused on the taxonomic composition of the microbiota, and how this was differentially affected across berberine doses. Berberine supplementation had few effects on the taxonomic composition at phylum level. In the jejunum, the supplementation with a low (*p* < 0.001) or high dose (*p* = 0.021) of berberine in the diet resulted in a reduction of the *Actinobacteria*. In the ileum, the lowest concentration of in-feed berberine induced an increase of the *Tenericutes*. No significant changes were found in the caecum or colon.

At family level, a low dose of berberine increased the relative abundance of *Bacillaceae*, Burkholderiaceae, Peptostreptococcaceae, Pseudomonadaceae, Ruminococcaceae and an uncultured family of the phylum *Tenericutes* in the jejunum (Supplementary Table S4). At genus level, this dose decreased *Gordonibacter*, *Curtobacterium* and *Butyricicoccus* in the jejunum, while it tended to increase Lachnospiraceae members *ASF356* and *CHKCI001* in the caecum and significantly increased *GCA-900066575*, Lachnospiraceae *UCG-010* (family Lachnospiraceae), Ruminococcaceae *UCG-014* (family Ruminococcaceae) in the colon (Supplementary Table S5).

The medium dose of berberine also stimulated genera of the Lachnospiraceae family in the caecum, including *ASF356*, *CHKCI001*, Family*_*Lachnospiraceae and *Fusicatenibacter*.

The highest dose of berberine decreases the relative abundance of the family Peptostreptococcaceae in the ileum, caecum and colon, a shift that was mostly due to an uncultured bacterium from this family (Supplementary Table S4, S5), that seems to identify as *Romboutsia timonensis* (>98% ID BLAST). In addition, it increased the Lachnospiraceae and Ruminococcaceae in the ileum. At genus level, the highest dose decreased the relative abundance of several low abundant bacteria (<0.1% relative abundance) in the ileum, including *Ralstonia* (Burkholderiaceae), *Cronobacter*, *Serratia* (Enterobacteriaceae), *Lachnoclostridium* 5, *Tyzzerella* (Lachnospiraceae), *Curtobacterium* (Microbacteriaceae), *Glutamicibacter* (Micrococcaceae) and *Faecalibacterium* (Ruminococcaceae) (Supplementary Table S5).

**FIGURE 3:**
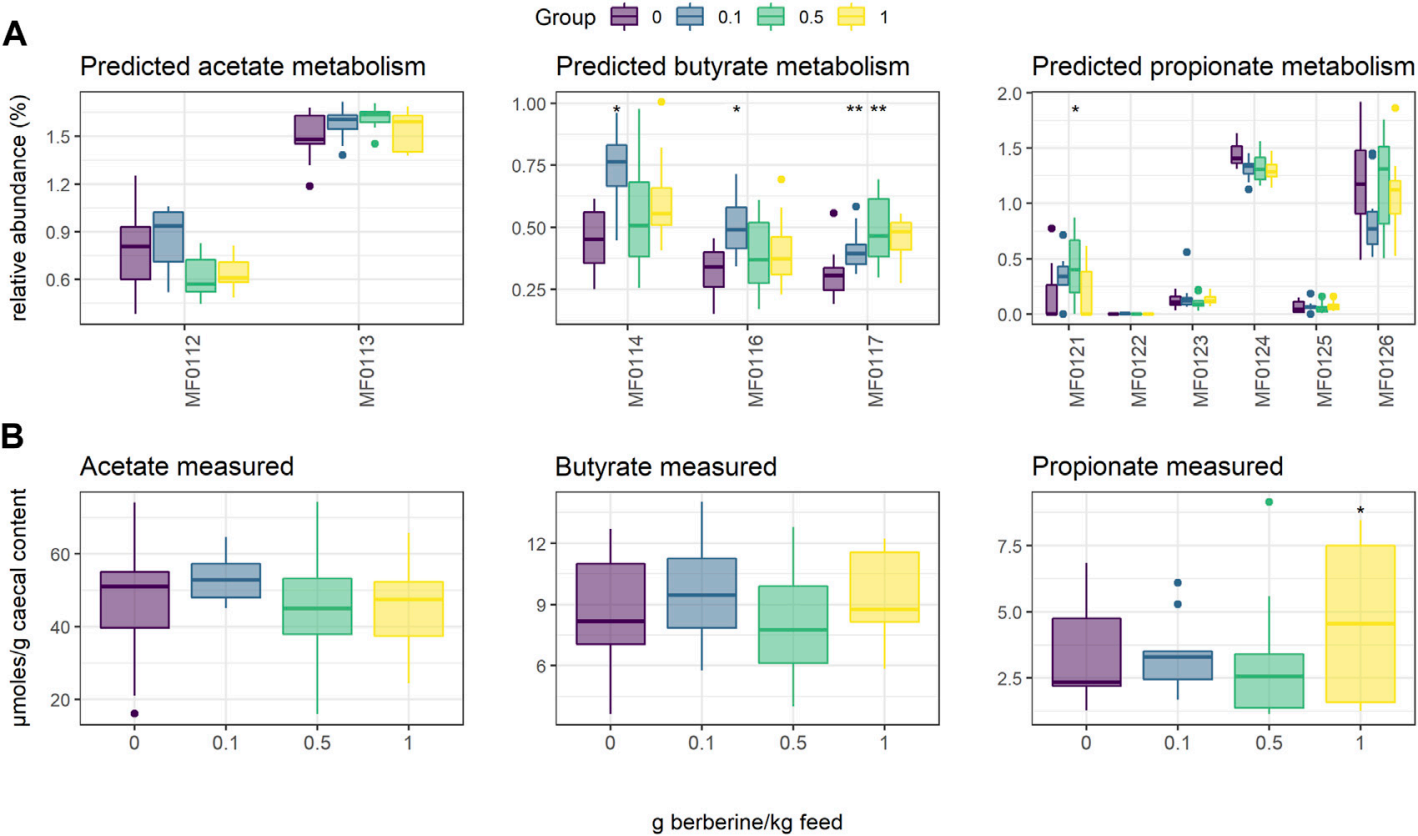
Short-Chain Fatty Acid (SCFA) metabolism in the caecum of chickens fed a diet supplemented with 0, 0.1, 0.5 or 1 g berberine/kg feed for 21 days post-hatch. **(A)** Relative abundance of predicted Gut-specific Metabolic Modules associated with acetate, butyrate or propionate metabolism. **(B)** Acetate, butyrate or propionate quantified via GC-FID. MF0112: acetate to acetyl-CoA; MF0113: acetyl-CoA to acetate; MF0114: acetyl-CoA to crotonyl-CoA, MF0116: butyrate production via transferase; MF0117: butyrate production via kinase; MF0121: propionate production (acrylate pathway); MF0122: propionate production (succinate pathway); MF0123: propionate production (propanediol pathway); MF0124: fucose degradation; MF0125: propionate production via kinase; MF0126: propionate production via transferase. *: *p* ≤ 0.05 vs. control; **: *p* < 0.01 vs. control.

Short-chain fatty acids (SCFAs), including acetate, butyrate and propionate, are produced by the gut microbiota. To evaluate whether the different concentrations of berberine influenced SCFA metabolism, we used PICRUST to predict *in silico* molecular functions, represented by KEGG orthologs (KOs), from 16S rRNA gene profiles. Assigned KOs were further aggregated in Gut-specific Metabolic Modules (GMMs), and GMMs related to acetate, butyrate or propionate metabolism in the caecum were investigated. The low-dose supplementation of berberine significantly increased the relative abundance of all predicted butyrate pathways (MF0114: *p* = 0.019; MF0116: *p* = 0.016; MF0117: *p* = 0.003) and tended to decrease production of propionate from fucose (MF0124: *p* = 0.056) (Figure 3). The middle-dose also increased butyrate production via kinase (MF0117: *p* = 0.008), as well as propionate production via the acrylate pathway (MF0121: *p* = 0.036), and tended to increase acetate metabolism (MF0113: *p* = 0.078). The high dose tended to increase butyrate production via kinase (MF0117: *p* = 0.065). SCFAs were also quantified in the caecum of animals but did not significantly reflect the functional predictions. Only propionate was significantly increased by the high dose of berberine (*p* = 0.045).

### Phase II berberine metabolites dominate in plasma, and berberine metabolism is more present in the caecum than in the ileum

While berberine can influence the composition of the gut microbiota, the latter can also transform berberine. The contradiction between the definite biological actions of berberine and its very low plasma concentration promoted the hypothesis that the metabolites of berberine may also contribute to its bioactivities. Metabolites were therefore quantified to help understanding the pharmacological activity of berberine *in vivo*. Berberrubine, thalifendine, demethyleneberberine, jatrorrhizine, columbamine and palmatine are main phase I metabolites of berberine *in vivo*, detected in feces and plasma samples after oral administration of berberine. Dihydroberberine and oxyberberine are generally described as gut microbiota-mediated and dihydroberberine has only been detected in intestinal content. A newly developed UPLC-MS/MS method was successfully applied for the quantification of berberine and its eight phase I metabolites, as well as their associated glucuronide and sulfate forms from phase II metabolism, in plasma and intestinal content (ileum, caecum) from chickens continuously fed with different doses of berberine for 21 days post-hatch (Supplementary Method S1). As demethyleneberberine presents different glucuronidation and sulfation sites in its structure, multiple conjugated forms were detected for this metabolite.

**FIGURE 4:**
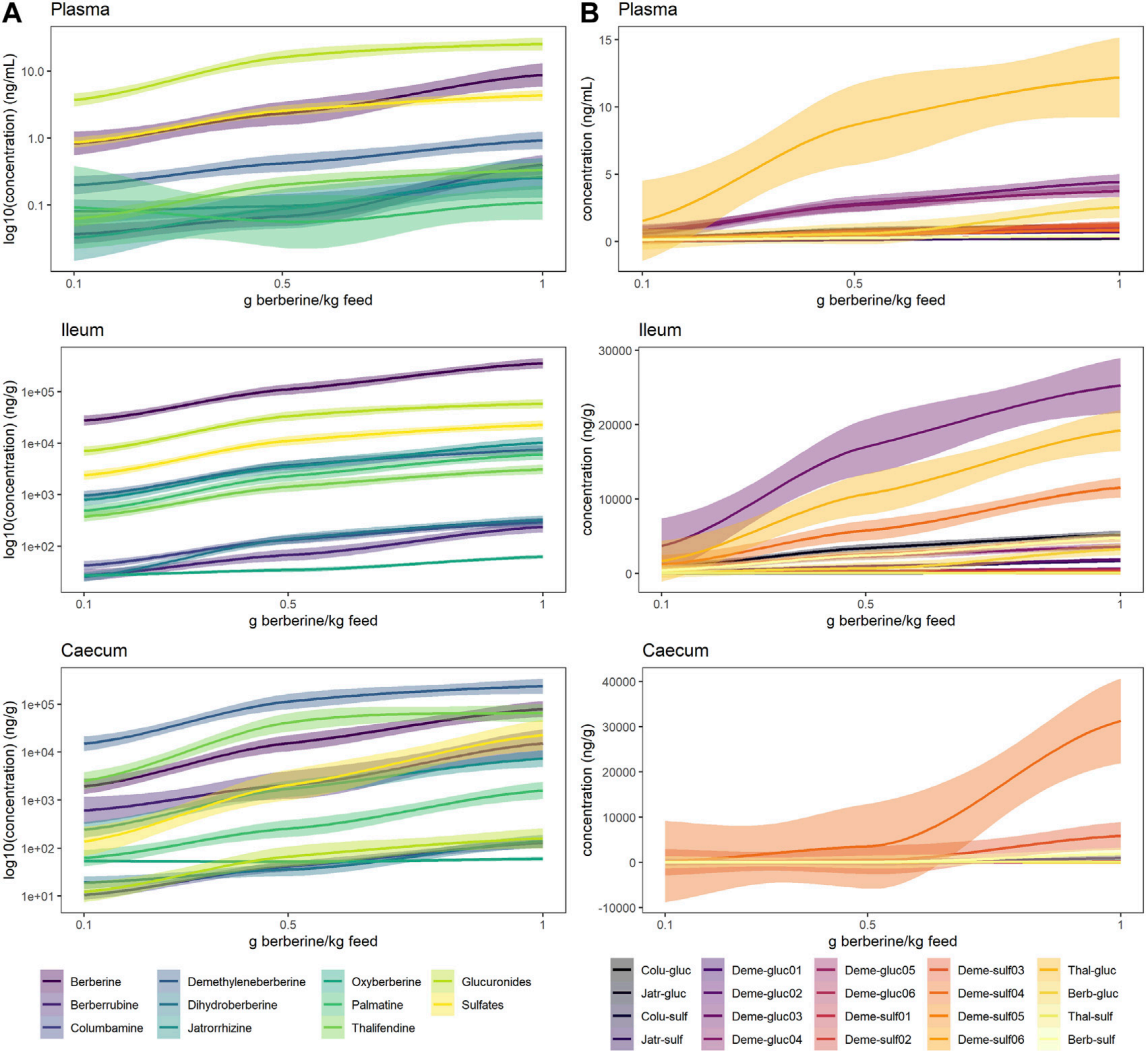
Berberine and berberine-derived metabolites in plasma (top), ileum (middle) or caecum (bottom) from chickens fed a berberine-supplemented diet (0.1, 0.5 or 1 g/kg feed) for 21 days post-hatch (n = 12). **(A)** Log_10_ concentration profiles of berberine, phase I and cumulated phase II berberine metabolites. **(B)** Concentration profiles of phase II glucuronide and sulfate berberine metabolites. The shade area represents the 95% confidence interval. Total glucuronides and sulfates represent cumulated phase II berberine metabolites and include berberrubine-glucuronide/-sulfate, columbamine-glucuronide/-sulfate, jatrorrhizine-glucuronide/-sulfate, demethyleneberberine-glucuronide/-sulfate01/02/03/04/05/06, thalifendine-glucuronide/-sulfate. Oxyberberine, columbamine, demethyleneberberine_sulfate01/02/05/06 were not detected in the plasma. Colu-gluc/-sulf: columbamine-glucuronide/-sulfate; Jatr-gluc/-sulf: jatrorrhizine-glucuronide/-sulfate; Deme-gluc/-sulf: demethyleneberberine-glucuronide/-sulfate; Thal-gluc: thalifendine-glucuronide; Berb-gluc/-sulf: berberrubine-glucuronide/-sulfate.

In plasma, metabolite concentrations globally increased with the dose of berberine in the feed (Figure 4, Supplementary Figure S2, S3). The concentration of phase II metabolites exceeded the one of phase I metabolites but was in the same range as the one of berberine (Figure 4A). Thalifendine-glucuronide and demethyleneberberine-glucuronides (_03, _04) were the most abundant metabolites in plasma (Figure 4B). When considering only phase I metabolites, demethyleneberberine was the most abundant in plasma. Dihydroberberine was effectively detected in plasma, in contradiction with previous results. Dihydroberberine is a very unstable metabolite, that oxidizes back to berberine up to 66% within 24 h (Supplementary Figure S4). The addition of ascorbic acid (vitamin C), an antioxidant, allowed to stabilize this metabolite during the extraction process and to further detect it in plasma. Oxyberberine was not detected. Columbamine was below LOQ, therefore not significantly present in plasma in berberine-supplemented groups. Berberrubine, jatrorrhizine and palmatine could also not be quantified in plasma for the lowest berberine supplementation level. Palmatine was also below LOQ in plasma for the middle berberine supplementation level.

In the intestinal content, all metabolites were significantly quantified. In the ileum, berberine was largely predominant compared to its phase I or phase II metabolites (Figure 4A). Phase II metabolites demethyleneberberine-glucuronide_03 and thalifendine-glucuronide were the most abundant metabolites in the ileum (Figure 4B), like in the plasma, probably originating from liver metabolism and enterohepatic circulation, where metabolites produced by the liver enter the small intestinal lumen via the bile duct. Jatrorrhizine, demethyleneberberine and palmatine showed the highest concentration among the phase I metabolites detected in the ileum.

In the caecum, demethyleneberberine concentration exceeded the one of berberine (Figure 4A). Thalifendine, berberrubine and jatrorrhizine were the next most abundant phase I metabolites. Sulfate conjugates of demethyleneberberine (_03, _04) were the most abundant phase II metabolites (Figure 4B).

**FIGURE 5:**
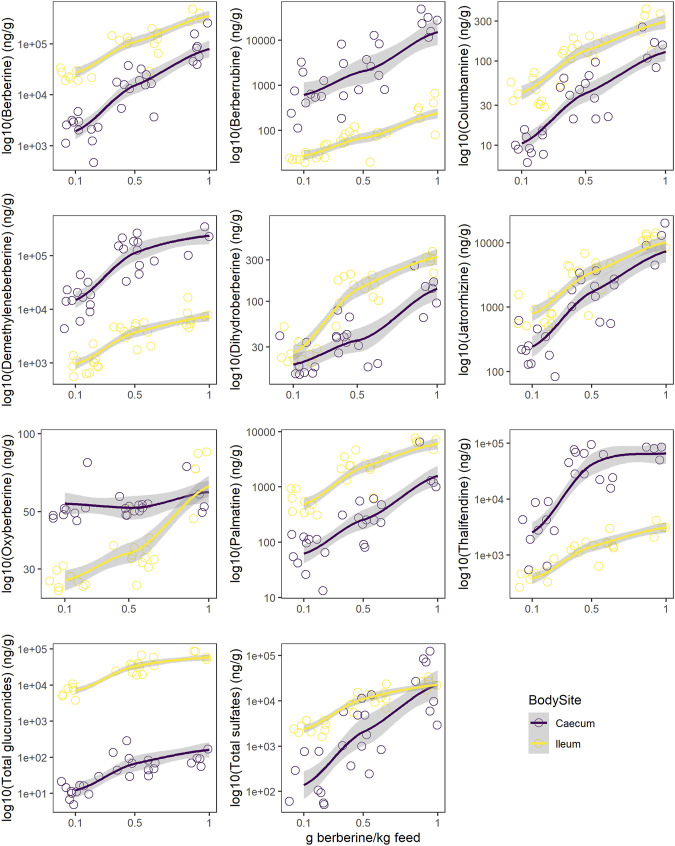
Comparison of log_10_ concentration profiles of berberine and berberine-derived metabolites between ileum (yellow) and caecum (purple) from chickens fed a berberine-supplemented diet (0.1, 0.5 or 1 g/kg feed) for 21 days post-hatch (n = 12). Total glucuronides and sulfates represent cumulated phase II berberine metabolites and include berberrubine-glucuronide/-sulfate, columbamine-glucuronide/-sulfate, jatrorrhizine-glucuronide/-sulfate, demethyleneberberine-glucuronide/-sulfate_01/_02/_03/_04/_05/_05/_06, thalifendine-glucuronide/-sulfate. Supplementary Figure S2 reports concentrations profiles in plasma.

Berberine concentration in the caecum was lower as compared to the ileum (Figure 5, Supplementary Table S6). All glucuronide conjugates were much lower in the caecum as compared to the ileum (Supplementary Figure S5, Supplementary Table S6), probably resulting from a more extensive β-glucuronidase and sulfatase activity in the caecum (Supplementary Figure S6). The same was globally observed for sulfate conjugates, except for demethyleneberberine-sulfate_04 and _06. Demethyleneberberine-sulfate_04 tended to increase in the caecum for the highest berberine supplementation level, while the concentration of demethyleneberberine_sulfate_06 increased when 0.1 and 0.5 g berberine was supplemented per kilogram feed. Demethyleneberberine, berberrubine and thalifendine were more concentrated in the caecum than the ileum (Figure 5, Supplementary Table S6). Spearman correlation analysis showed that the concentration of these metabolites in the caecum are highly correlated with the concentration of their corresponding glucuronide or sulfate conjugates in the ileum (*p* < 0.001), meaning that demethyleneberberine, berberrubine and thalifendine partly originated from the deconjugation of their corresponding glucuronide or sulfate forms by β-glucuronidases and sulfatases. The concentration of dihydroberberine, columbamine, jatrorrhizine and palmatine globally decreased in the caecum in comparison with the ileum (Figure 5, Supplementary Table S6). The demethylation of palmatine to jatrorrhizine and columbamine, further demethylated into demethyleneberberine, might also explain the increase of demethyleneberberine in the caecum at the expense of these other metabolites (He et al., 2017). Thalifendine-sulfate, columbamine-sulfate, demethyleneberberine-sulfates_01, _02, _05, _06 were sulfate conjugates measured in the intestinal content that were not detected in the plasma.

### Phase I and phase II berberine metabolites in plasma are associated with improved gut health

**FIGURE 6:**
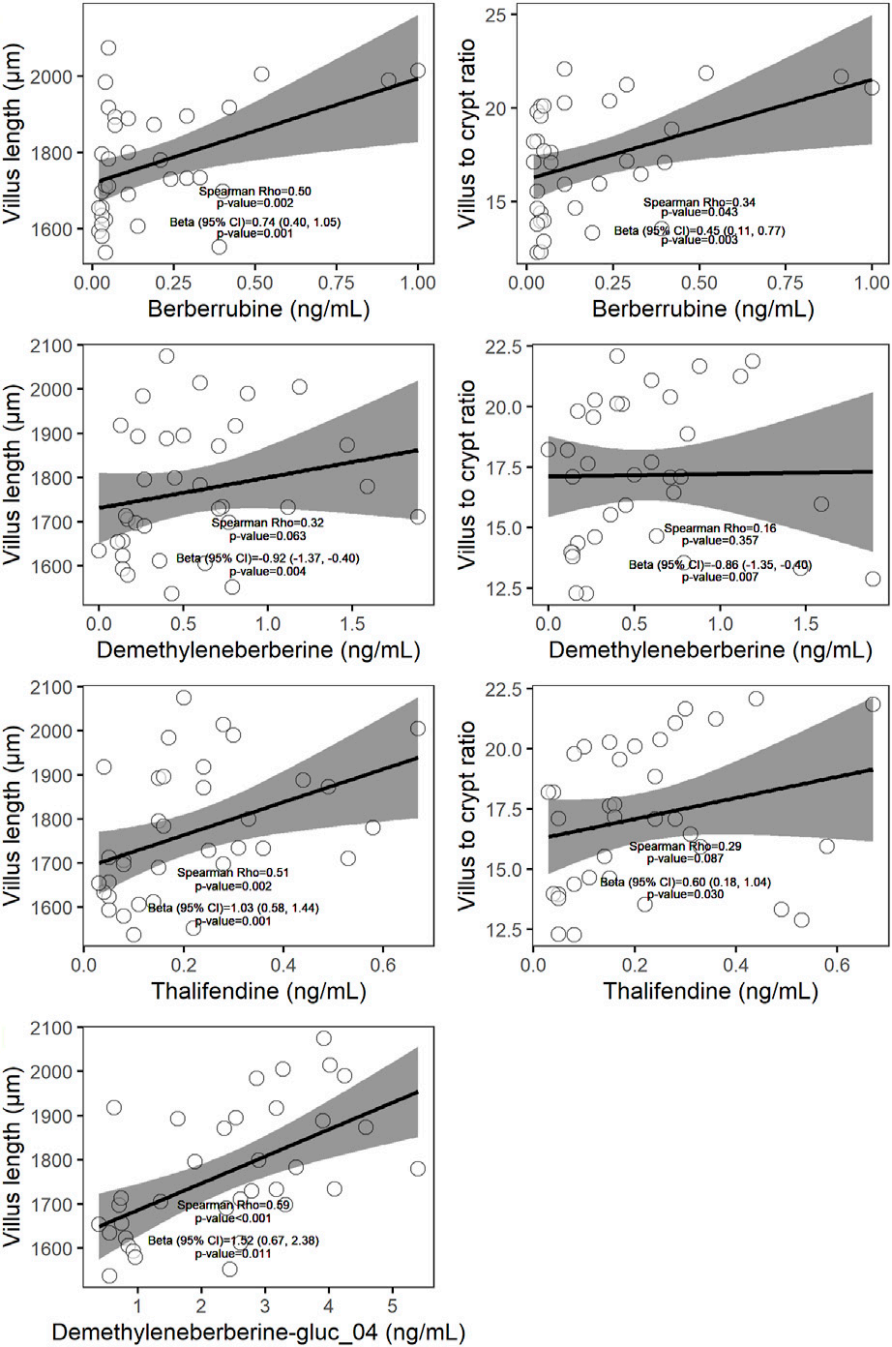
Scatter plots of plasma metabolites (x-axis) and histological parameter (y-axis) measured in chickens fed a berberine-supplemented diet (N = 36). On each scatter plot the Spearman correlation coefficient and the corresponding *p*-value are shown. The β coefficient (standardized regression coefficient), 95% confidence interval (CI), and *p*-value shown are derived from the linear mixed-effects models (LMM) predicting each of the histological parameter value adjusted for plasma metabolites, dose of berberine in the feed and pen. Only relationships showing significance (*p* < 0.05) using LMM were plotted.

Berberine-derived metabolites can interact with the enterocytes, via absorption of intestinal-derived metabolites by the gut mucosa or via distribution of circulating metabolites originating from the intestine or liver into the gut tissue. To investigate whether berberine metabolites could contribute to the beneficial morphological changes of the gut barrier observed after dietary berberine administration, a linear mixed-effects model (LMM) including berberine and either its phase I metabolites, phase II glucuronide forms or phase II sulfate forms in plasma was performed for each morphological parameter of the duodenum. Berberrubine, thalifendine and demethyleneberberine in plasma were shown to be positively associated with the villus length and the villus to crypt ratio in the duodenum (Figure 6). One conjugated form of demethyleneberberine, demethyleneberberine-glucuronide_04 was also positively associated with the villus length.

### The large intestinal microbiota is a major player of berberine metabolism

**FIGURE 7:**
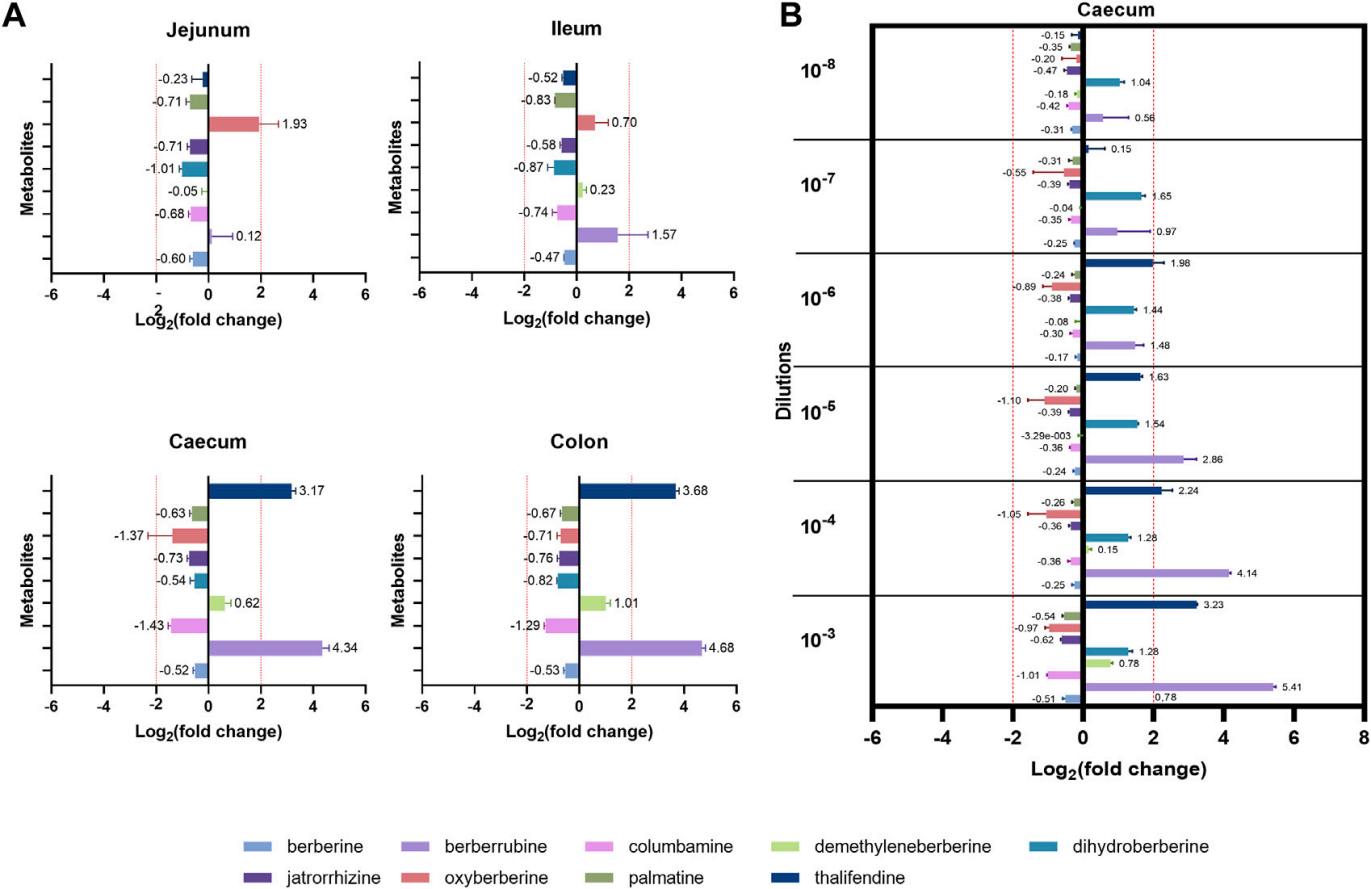
Metabolite production by the gut microbiota *in vitro*. **(A)** Gut microbiota from different intestinal segments of the chicken. Luminal content isolated from the jejunum, ileum, caecum or colon of two 3-week-old broiler chickens was diluted (1:100 for the jejunum, 1:1,000 for the other segments) in M2GSC medium pH 6 containing 10 µg berberine/mL and cultured for 48 h in anaerobic conditions. **(B)** Different dilutions of the chicken caecal microbiota. Caecal content of a 4-week-old broiler chicken was 10-fold serial diluted from dilution 10^−3^ to 10^−8^, inoculated in M2GSC medium pH 6 containing 10 µg berberine/mL, and cultured for 48 h in anaerobic conditions. Metabolite levels were assessed using a semi-quantitative UPLC-MS/MS method at the beginning and at the end of the experiment, and metabolite production was expressed as log_2_ fold change, calculated as the log_2_ of the ratio of the metabolite response at t = 48 h over the metabolite response at t = 0 h. Data are shown as the mean ± standard deviation (n = 3). An absolute fold change >4 (|log_2_(fold change)| > 2) was considered as biologically relevant.

While berberine metabolites showed to be associated with intestinal health parameters, their origin is not yet fully clear. We showed that the caecum is an important site of berberine metabolism, where the concentration of phase I metabolites is greater or in the same range than the one of the parent molecule, berberine. While those metabolites seem to originate partly from the deconjugation of their glucurono- and sulfo-conjugates by bacterial glucuronidases and sulfatases, the role of intestinal bacteria in their primary production was further investigated. Berberine-derived metabolites were evaluated in cultures of jejunal, ileal, caecal or colonic microbiota containing a non-inhibitory concentration of berberine, to identify the intestinal compartment-specific metabolism of berberine (Figure 7A). The caecum and the colon were the most metabolically active intestinal segments in terms of berberine transformation and production of the demethylated metabolites thalifendine and berberrubine, as well as demethyleneberberine to a lesser extent. Oxyberberine was slightly produced in the small intestine.

To confirm the role of the microbiota in berberine metabolism, increasing dilutions of the caecal content from a 4-week-old broiler chicken were used as inocula in the berberine-containing medium (Figure 7B). Thalifendine and berberrubine were again the main metabolites generated in the caecum, and their production decreased when the intestinal bacterial load decreased. No relevant metabolite production was noticed from the dilution 10^−7^. A slight, consistent production of dihydroberberine was also observed across the different dilutions, which was not observed in the previous experimental set-up. As previously, a slight production of demethyleneberberine was observed in the lowest dilution. Similar signals of jatrorrhizine and palmatine were observed in cultures with berberine and in the control berberine medium, confirming that they are impurities and that they are not produced by the microbiota (Supplementary Figure S7).

### Intestinal epithelial cells produce different metabolites than the microbiota and utilize microbiota-derived metabolites

**FIGURE 8:**
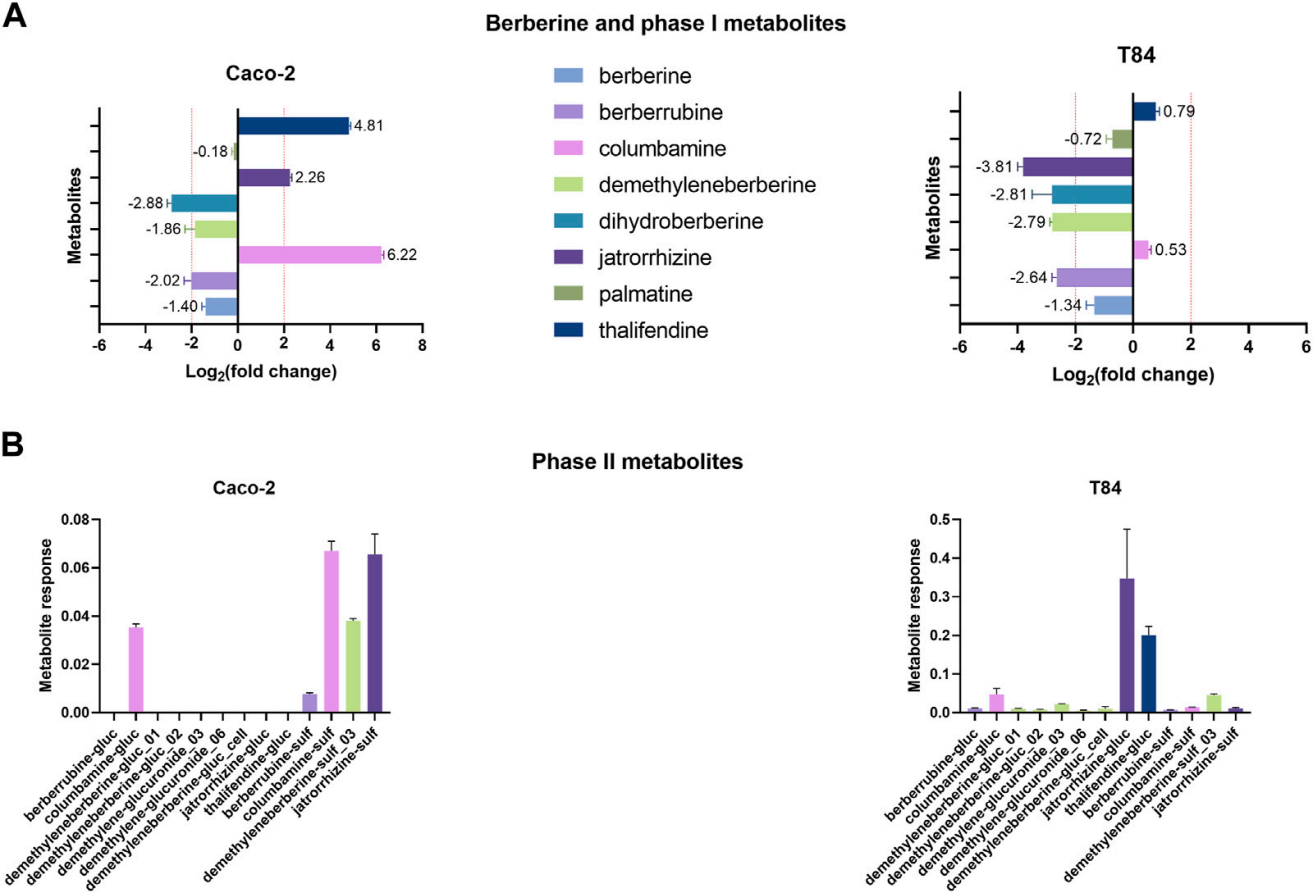
Metabolite production by intestinal epithelial cells *in vitro*. **(A)** Phase I metabolites. **(B)** Phase II metabolites. 24-h differentiated cells were incubated with 0.1 μg/mL berberine and phase I and phase II metabolite response was evaluated after 48 h using a semi-quantitative UPLC-MS/MS method, in the cell cultures and in the berberine control medium (well without cells). Metabolite production was expressed a log_2_ fold change, calculated as the log2 of the ratio of the metabolite response in the cell culture at t = 48 h over the metabolite response in the berberine control medium at t = 48 h. Data are shown as the mean ± standard deviation (n = 3). An absolute fold change >4 (|log_2_(fold change)| > 2) was considered as biologically relevant. Phase II metabolite production is expressed as the raw response, as there is a strict absence of signal in the control medium. Demethyleneberberine-gluc_cell was not detected in any previous *in vivo* samples.

Two human intestinal epithelial cells lines were used to investigate the contribution of gut epithelial cells to berberine metabolism. Caco-2 cells were used as a model for small intestinal enterocytes whereas T84 cells resemble colonocytes (Devriese et al., 2017). According to the results of the cytotoxicity tests, the concentration of 0.1 µg berberine/mL medium, as used in further experiments, was considered safe for the cells (Supplementary Figue S8). Incubation of Caco-2 cells with berberine led to the production of columbamine, thalifendine and jatrorrhizine (Figure 8A). Signals of dihydroberberine and berberrubine were simultaneously reduced, meaning that the cells either absorbed or converted those metabolites, present as impurities in the starting berberine solution. Caco-2 cells produce very few conjugated metabolites, mainly sulfate forms (Figure 8B). On the contrary, in T84 cell cultures, no phase I metabolites were generated but the levels of jatrorrhizine, dihydroberberine, demethyleneberberine and berberrubine decreased (Figure 8A). This was associated mainly with a production of jatrorrhizine, thalifendine and columbamine glucuronides and demethyleneberberine-sulfate_03 (Figure 8B). Oxyberberine was not detected in any of the cell cultures. Berberrubine, and possibly dihydroberberine, were therefore microbiota-derived metabolites that can be further transformed by intestinal cells, suggesting a metabolic crosstalk. Columbamine was not produced in the microbiota cultures, making it a host-derived metabolite that might further interact with the gut microbiota or be directly converted to its conjugated form. Thalifendine was produced both by the gut microbiota and by the enterocytes.

### Berberine metabolites are associated with specific microbial taxa in the caecum

**FIGURE 9:**
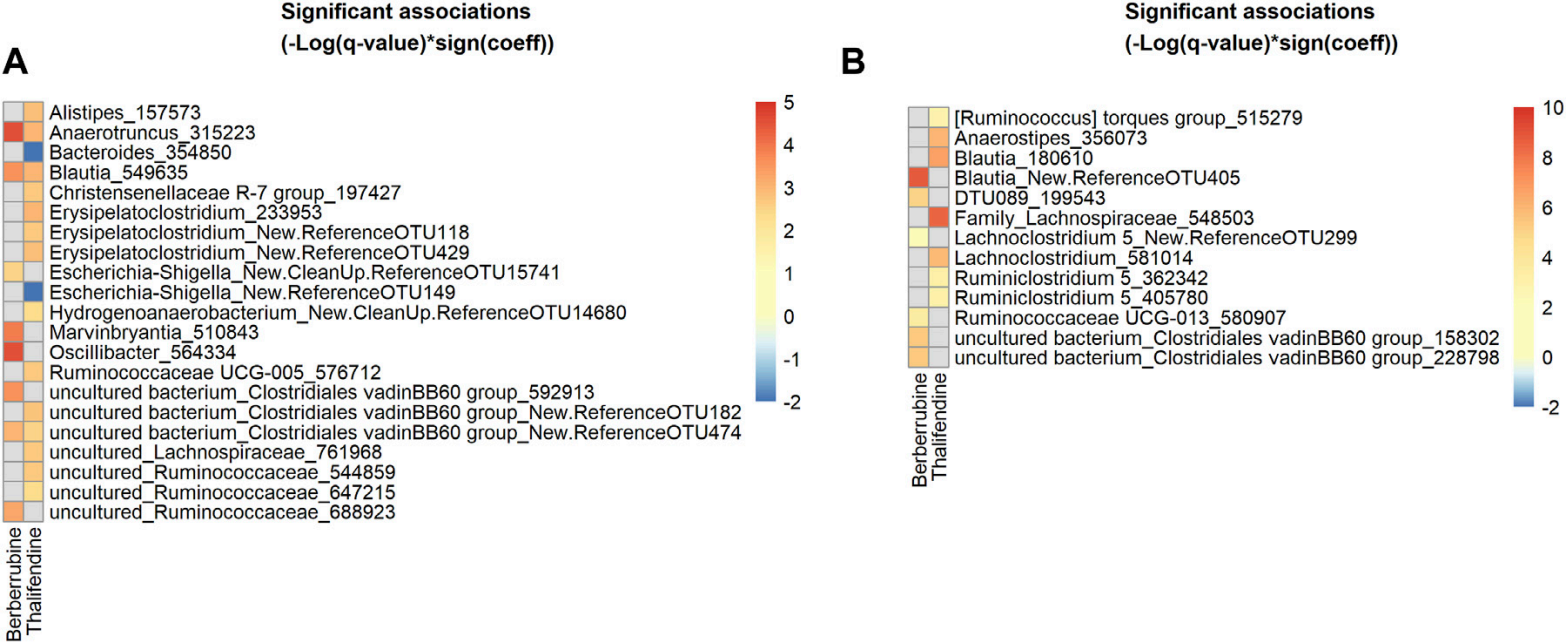
Heatmap of bacterial OTUs showing significant association with berberrubine or thalifendine *in vitro* and *in vivo*. **(A)** Active OTUs in caecal microbiota cultures (N = 12) **(B)** OTUs in the caecum of chickens fed 0.1 or 0.5 g berberine/kg feed for 21 days post-hatch (N = 24). Significant associations (q-value <0.1) were identified using MaAsLin2 and are plotted as(–Log(q-value)*sign(coeff.)). Grey squares: no significant association. A BLAST search could identify the species level of some OTUs showing the most significant associations (q < 0.05) for **(A)**: 315223, *Anaerotruncus colihominis* (100%); 549635, *Blautia glucerasea* (99.75%) and **(B)**: New.ReferenceOTU405, *Murimonas intestini*/*Blautia faecicola* (97.03/97.01%); 548503, [*Ruminococcus*] *lactaris*/*Fusicatenibacter saccharivorans* (97.26/97.01%); 180610, *Blautia stercosis* (97.01%); 356073, *Anaerostipes butyraticus* (100%); 581014, *Enterocloster clostridioformis*/*boltae*/*Lachnoclostridium pacaense*/*Kineothrix alysoides*/*Lacrimispora amygdalina* (97.51%), 199543, *Anaeromassilibacillus senegalensis* (98.26%); 580907, *Monoglobulus pectinilyticus* (97.76%).

We showed that berberrubine and thalifendine were microbiota-derived metabolites of berberine. In order to identify taxa associated with their production, we mapped the active bacterial community present in the *in vitro* caecal cultures, via 16S rRNA gene amplicon sequencing of the corresponding cDNA. Associations between thalifendine or berberrubine levels and microbial abundances of active OTUs were analysed using multivariate association with linear models (MaAsLin2) (Figure 9A). Data generated from the 10^−7^- and 10^−8^- diluted caecal inocula were excluded from the analysis as no significant metabolite production was observed in those dilutions. OTUs sequences that showed a highly significant association with a metabolite were submitted to BLAST and alignment results for which the percent identity was superior to 97% were reported when available. Highly significant positive associations (q < 0.05) were found between berberrubine and *Anaerotruncus colihominis* (100% ID), *Oscillibacter* and an uncultured bacterium from the family Ruminococcaceae, *Marvinbryantia* and *Blautia glucerasea* (99.75% ID) from the family Lachnospiraceae, as well as two uncultured bacteria from the family *Clostridiales vadin BB60 group*. More particularly, *Anaerotruncus* and *Oscillibacter* are OTUs that were overrepresented in the active community (Supplementary Table S7).

To verify *in vitro* results, the same analysis was performed on 16S rRNA gene sequencing and berberrubine or thalifendine data from the caecal samples of chickens supplemented with berberine (Figure 9B). The group fed the highest concentration of berberine was excluded from the analysis as it showed to have some antibacterial effects and therefore might lead to bias. Highly significant positive associations (q < 0.05) were found between berberrubine and OTUs belonging to the genera *Blautia* (*Murimonas intestini*/*Blautia faecicola* 97.03/97.01% ID), uncultured bacterium from the *Clostridiales vadin BB60 group*, *DTU089* (*Anaeromassilibacillus senegalensis* 98.26% ID) and *Ruminoccocaceae UCG-013* (*Monoglobulus pectinilyticus* 97.76% ID). Thalifendine was positively correlated (q < 0.05) with *Blautia stercosis* (97.01% ID), *Anaerostipes butyricaticus* (100% ID), as well as OTUs of the genera Family_Lachnospiraceae ([*Ruminococcus*] *lactaris*/*Fusicatenibacter saccharivorans* 97.26/97.01% ID), *Lachnoclostridium* and *Ruminiclostridium 5*. OTUs belonging to the genera *Blautia*, *C. vadin BB60 group* and undefined genera from the family Lachnospiraceae were associated with the presence of berberine metabolites both *in vitro* and *in vivo* (Supplementary Table S8).

### Acetogenic bacteria convert berberine to its demethylated metabolites

**FIGURE 10:**
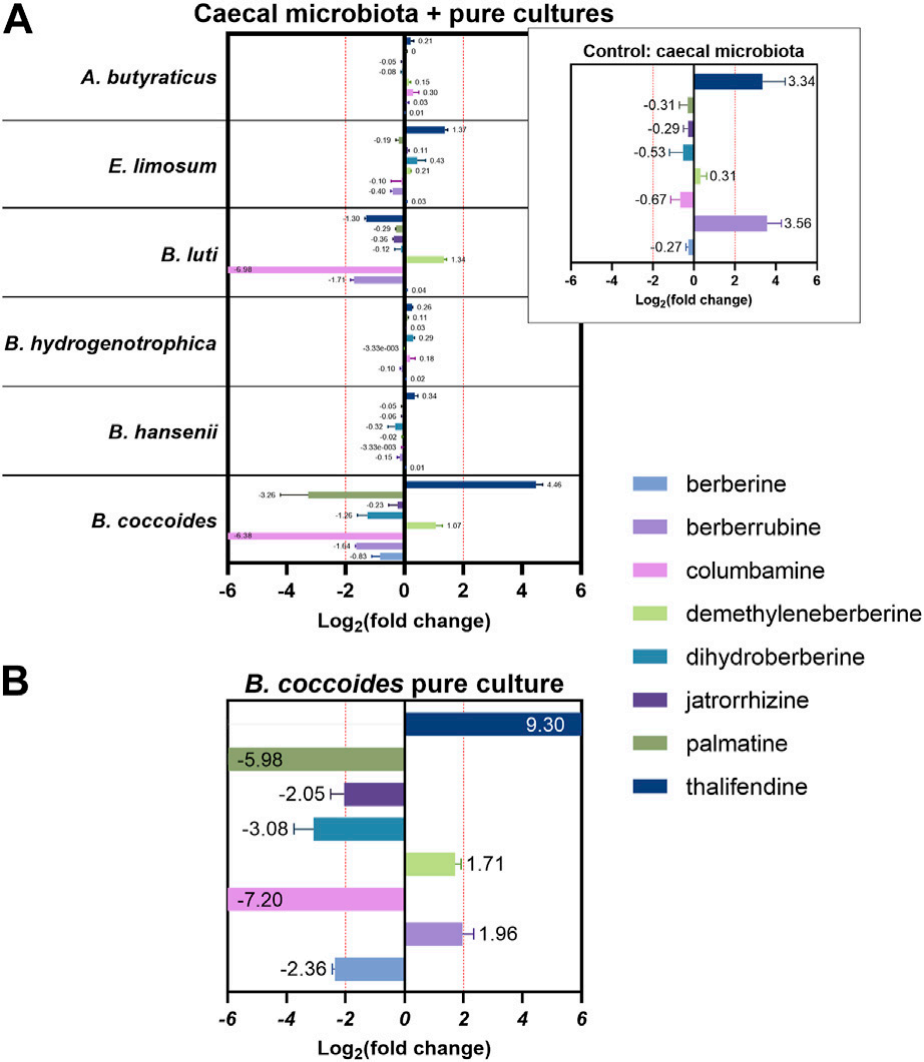
Metabolite production *in vitro* **(A)** in caecal microbiota cultures supplemented with pure cultures of bacteria **(B)** in *Blautia coccoides* pure culture. For mixed cultures, 1:100 diluted overnight cultures of bacteria were added to M2GSC medium pH 6 inoculated with 1:1,000 diluted caecal content, containing 10 µg berberine/mL and incubated 48 h. For *Blautia coccoides* pure culture, 1:250 diluted overnight culture was added to M2GSC medium pH 6 containing 10 µg berberine/mL and incubated 48 h. Metabolite levels were assessed using a semi-quantitative UPLC-MS/MS method, and metabolite production was expressed as log_2_ fold change, calculated as the log_2_ of the ratio of the metabolite response at t = 48 h in the cultures supplemented with bacteria over the metabolite response at t = 48 h in the control (berberine-medium + microbiota or berberine-medium). Data are shown as the mean ± standard deviation (n = 3). An absolute fold change >4 (|log_2_(fold change)| > 2) was considered as biologically relevant.

*In silico*, several OTUs of the genus *Blautia* were associated with levels of berberine demethylated metabolites, berberrubine and thalifendine. *Blautia* spp. are acetogenic bacteria, which use one-carbon substrate to produce acetate via the Wood-Ljungdahl pathway. One-carbon compounds can include H_2_ + CO_2_, formate, and methyl groups from aryl methyl ethers (Ragsdale and Pierce, 2008). Due to its structure, berberine could be a methyl donor in this pathway. Therefore, we investigated whether acetogenic bacteria could carry out the demethylation of berberine *in vitro*. Four different *Blautia* species (*B*. *coccoides*, *B*. *hansenii*, *B*. *hydrogenotrophica* and *B*. *luti*), as well as another characterized acetogen, *E. limosum*, and the non-acetogenic bacterium previously associated with thalifendine, *A. butyraticus*, were tested for their metabolic capacities (Figure 10A). Medium containing berberine was inoculated with caecal microbiota to mimic the gut environment and further enriched with pure cultures of the bacteria above. The concentration of berberine was preliminary tested not to affect the growth of tested bacteria (Supplementary Figure S1). Reproducing our previous results, caecal microbiota alone generated high levels of berberrubine and thalifendine (Figure 10A). The addition of *B. coccoides* significantly increased the signal of thalifendine. A slight production of thalifendine was also observed with *E. limosum*. *B. coccoides* and *B. luti* also generated slight levels of demethyleneberberine, probably coming from the demethylation of palmatine/columbamine, while berberrubine signal decreased. Other *Blautia* species, *B. hydrogenotrophica* and *B. hansenii*, as well as the butyrate-producer *A. butyraticus*, did not show metabolization of berberine. In pure cultures, *B. coccoides* increased the signal of thalifendine and demethyleneberberine, but also of berberrubine (Figure 10B).

**FIGURE 11:**
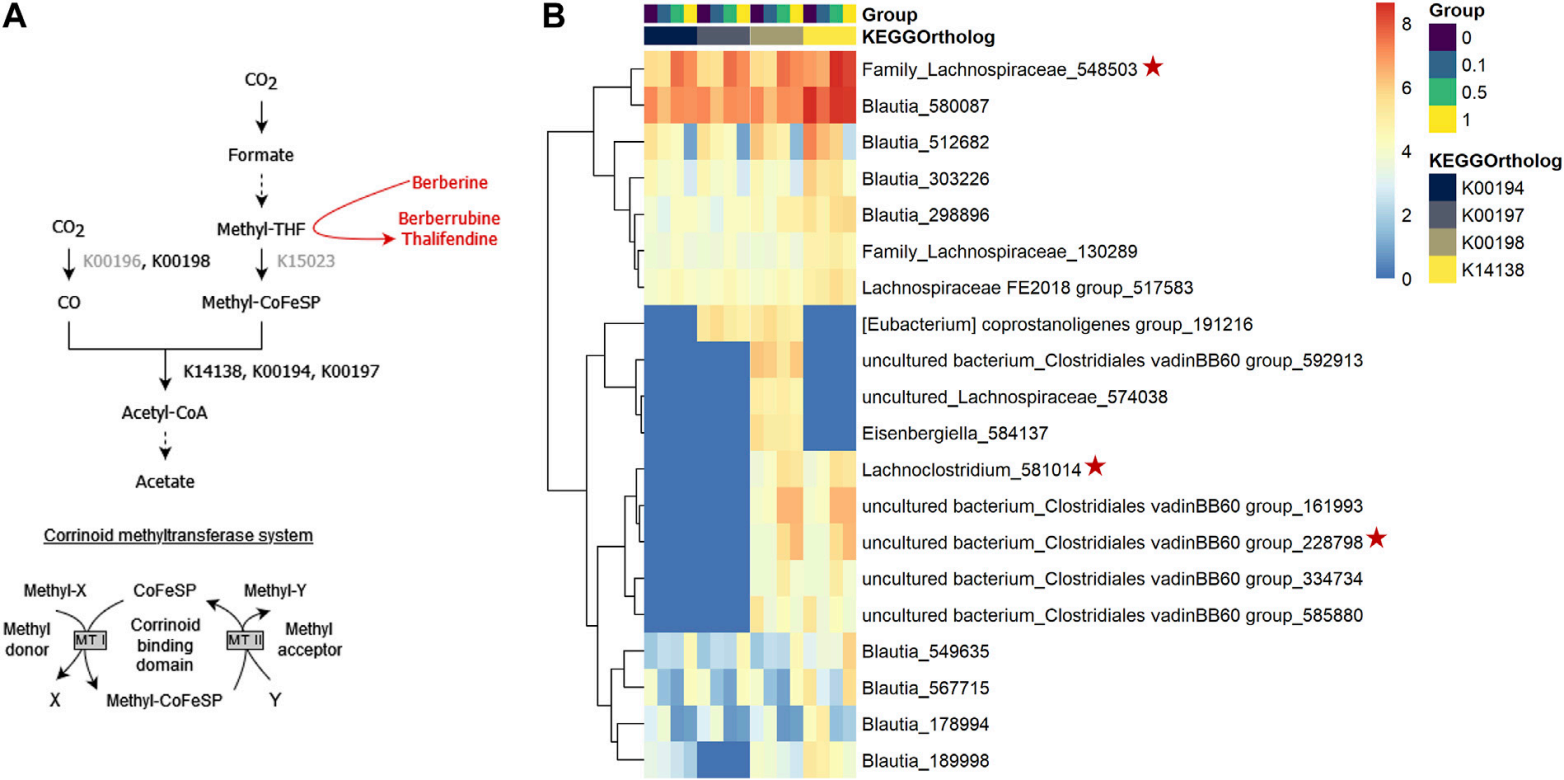
Proposed demethylation mechanism of berberine by acetogenic bacteria. **(A)** Wood-Ljungdahl pathway. The methyl group resulting from the demethylation of berberine into berberrubine/thalifendine (in red) might be shuttled into the Wood-Ljundahl pathway of acetogenic bacteria via a new corrinoid methyltransferase system. KEGG ortholog (KO) identifiers are assigned to the steps involving corrinoid-dependant methyltransferases. KOs in grey presented 0 counts in our metagenome predictions dataset. THF: tetrahydrofolate, CoFeSP: corrinoid iron-sulfur protein, MT: methyltransferase. Adapted from (Plichta et al., 2016). **(B)** OTUs contributing to predicted corrinoid-dependent methyltransferase activity in the caecum of chickens fed a control (0) or berberine-supplemented diet (0.1, 0.5 or 1 g berberine/kg feed) for 21 days. Metagenome contributions on the OTU level are sorted per KO and per group. The log_2_ of the gene counts per OTU are shown on a blue–red scale. Only OTUs contributing to log_2_ > 5 are shown. K00198: anaerobic carbon-monoxide dehydrogenase catalytic subunit, K14138: acetyl-coA synthase, K00194/K00197: acetyl-CoA decarbonylase/synthase. Red stars indicate OTUs that correlated with berberrubine or thalifendine with Maaslin2 (Figure 9B).

When acetogens grow on H_2_ + CO_2_, CO_2_ is reduced to formate and further to methyl-tetrahydrofolate (methyl-THF), which serves as the precursor of the methyl group of acetyl-CoA, which will lead to acetate, and involves a corrinoid-dependent methyltransferase system (Figure 11A). Acetogenic bacteria can grow on a variety of methyl donors by coupling different corrinoid-dependent methyltransferase systems to the Wood-Ljungdahl pathway (Figure 11A) (Matthews et al., 2008). While a system involved in transferring the methyl group from an aryl methyl ether to methyl-THF has already been reported, such system has not been described yet for berberine. We investigated the correlation between berberrubine or thalifendine and the remaining methyltransferases of the pathway based on *in vivo* data (Figure 11A). Only thalifendine showed positive associations with predicted methyltransferase activity (LMM *p*-values: K14138 *p* = 0.055; K00194 *p* = 0.0391; K00197 *p* = 0.059). OTUs belonging to the genus *Blautia* were important contributors to these methyltransferase-related KOs, as well as three OTUs previously correlated with either berberrubine or thalifendine levels, belonging to an undefined genus of the family Lachnospiraceae, the genus *Lachnosclostridium* and an undefined genus of the *Clostridiales vadinBB60 group* (Figure 11B).

### Transformation of berberine to its demethylated metabolites stimulates SCFA production

Owing to the ability of acetogens to convert berberine into thalifendine and to a lesser extent berberrubine, probably via the acetogenesis pathway, we sought to determine whether this conversion could drive acetate and more generally SCFA production in the caecum. The association between berberine, berberrubine or thalifendine concentration and the production of SCFAs was assessed *in vivo* with a correlation analysis (LMM) (Table 2). Berberine and thalifendine were significantly positively associated with acetate production. Berberrubine was significantly positively associated with butyrate and propionate in the caecum and tended to correlate with acetate (*p* = 0.0947).

**TABLE 2:** Correlation between berberine, berberrubine or thalifendine and SCFAs in the caecum of chickens fed 0.1 or 0.5 g berberine/kg feed for 21 days post-hatch (LMM, N = 24).

Metabolite	LMM	
	β coefficient (95% CI)	*p*-value
Acetate		
**Berberine**	0.89 (0.16, 1.62)	**0.0201**
**Berberrubine**	0.45 (−0.08, 0.99)	0.0947
**Thalifendine**	0.88 (0.17, 1.60)	**0.0189**
Butyrate		
**Berberine**	0.52 (−0.06, 1.15)	0.1492
**Berberrubine**	0.53 (0.12, 0.95)	**0.0273**
**Thalifendine**	0.38 (−0.24, 1.06)	0.3002
Propionate		
**Berberine**	−0.13 (−1.05, 0.79)	0.7691
**Berberrubine**	0.72 (0.22, 1.22)	**0.0075**
**Thalifendine**	−0.57 (−1.42, 0.29)	0.1800

Regression coefficients (β coefficients) and 95% confidence intervals (95% CI) were estimated from the standardized LMM.

Significant *p*-values are shown in bold.

**FIGURE 12:**
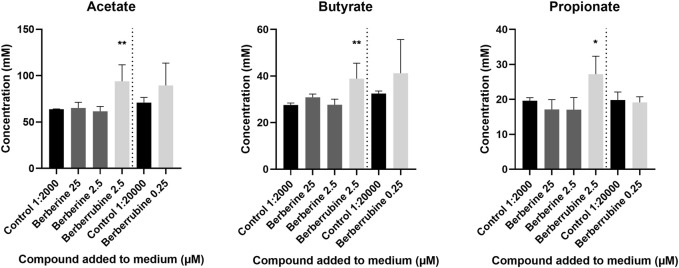
Short-Chain Fatty Acid (SCFA) production in caecal microbiota cultures supplemented with berberrubine (2.5, 0.25 µM) or berberine (25, 2.5 µM). Caecal microbiota was sampled from a 4-week-old broiler chicken and diluted 1:100 in M2GSC medium. 5 mM compound solutions were prepared in 1:10 DMSO:methanol and 1:2,000 (2.5 µM) or 1:20,000 (0.25 µM) diluted in M2GSC-microbiota medium. 1:10 DMSO:methanol 1:2,000 or 1:20,000 diluted in M2GSC-microbiota medium were used as controls for SCFA production. Data are expressed as the mean ± standard deviation (n = 3). *: *p* ≤ 0.05; **: *p* ≤ 0.01.

The ability of berberine demethylated metabolites to directly stimulate SCFA production was evaluated *in vitro* in cultures derived from caecal microbiota, after addition of a high (2.5 µM) or low (0.25 µM) concentration, and was compared to the one of berberine (25, 2.5 µM) (Figure 12). Only berberrubine could be tested as thalifendine was not commercially available. Berberrubine at the higher concentration increased the concentrations of acetate, butyrate and propionate, while berberine did not significantly affect SCFA production.

## Discussion

Low and medium concentrations of in-feed berberine stimulate beneficial microbiota. Low concentration (0.1 g/kg feed) of berberine increases the gut microbiota richness in the caecum and the colon. A high bacterial richness presumably reflects ecosystem stability and resilience and therefore is generally thought to characterise a healthy gut (Lozupone et al., 2012). In addition, multiple members of the Lachnospiraceae and Ruminococcaceae were increased in abundance in the gut of chicken receiving low or medium concentrations of in-feed berberine, including Lachnospiraceae members *GCA-900066575* and Lachnospiraceae *UCG-010*, and Ruminococcaceae *UCG-014* from the family *Ruminococcacaeae* in the colon. The medium concentration of berberine also enriched Lachnospiraceae genera *ASF356*, *CHKCI001* and *Fusicatenibacter* in the caecum. Ruminococcaceae *UCG-014* and *CHKCI001* were depicted as beneficial bacteria in birds, due to their positive association with butyrate production or animal performance respectively (Liao et al., 2020). *ASF356* was also previously described as a butyrate producer (Biggs et al., 2017). Butyrate is a SCFA that serves as a source of energy for intestinal epithelial cells and has beneficial health effects for the host (Griffiths et al., 2000; Guilloteau et al., 2010). One characterized species of the genus *Fusicatenibacter*, *Fusicabacter saccharivorans*, has been classified within the *Clostridium cluster XIVa*, a bacterial group that plays a major role in butyrate metabolism in the human intestine (Takada et al., 2013). This bacterium was notably decreased in ulcerative colitis human patients and its administration improved murine colitis (Takeshita et al., 2016). In conclusion, 0.1 and 0.5 g berberine/kg feed could stimulate bacteria with potential beneficial effects for the host. This effect was not observed for the highest concentration of berberine in feed, which particularly reduce bacteria of the Peptostreptococcaceae family, confirming previous results (Dehau et al., 2023).

Berberrubine, thalifendine, demethyleneberberine and its glucuronide form are associated with good intestinal health. These four berberine-derived metabolites positively correlated with villus length and villus-to-crypt ratios in the duodenum. Increased villus length translates as increased surface for nutrient absorption. Anti-inflammatory effects of berberrubine or demethyleneberberine were previously reported in DSS-induced mice colitis models. More particularly, direct administration of berberrubine or demethyleneberberine reduced the production of pro-inflammatory cytokines in the colon, and berberrubine also increased the expression of proteins playing a role in intestinal integrity (Chen et al., 2017; Yu et al., 2018). In addition, demethyleneberberine depicted anti-inflammatory and antioxidant effects in liver disease mice models, which might be due do its catechol group, a structural feature that has been associated with antioxidant activity (Zhang et al., 2015; Qiang et al., 2016; Zhang et al., 2020b). Both berberrubine and thalifendine preserve the methylenedioxy group after biotransformation of berberine and most biological activities of berberine have been attributed to that functional group. In addition, the modifications of berberine at C9 and C10 positions, as observed in these three metabolites, might lead to better anti-inflammatory effects (Singh et al., 2021). Demethyleneberberine glucuronide exhibited a comparable systemic exposure as compared to berberine, suggesting that it may be an important contributor to the biological effects of berberine *in vivo*, although individual effects of this metabolite have not been studied. Bioactivity has been observed for another phase II metabolite of berberine, berberrubine-glucuronide, which exhibited similar glucose-lowering effects compared to berberrubine and berberine (Yang et al., 2017). In addition, we showed that berberrubine stimulated butyrate fermentation in the caecum; this bacterial metabolite supplies energy to gut epithelial cells and has anti-inflammatory effects and is therefore essential for intestinal integrity. While there is no previous evidence of the ability of berberine metabolites to directly modulate SCFA metabolism, one study showed that the administration of berberrubine in mice increased the relative abundance of the butyrate-producer *Roseburia* (Yang et al., 2022). To sum up, the anti-inflammatory and antioxidant potentials of berberine metabolites might explain the optimal gut morphology observed after berberine supplementation.

Berberrubine is mainly derived from the transformation of berberine by the gut microbiota. Berberrubine and thalifendine were the main metabolites produced *in vitro* by caecal and colonic bacteria. This was confirmed *in vivo*, where thalifendine and berberrubine were among the most abundant phase I metabolites in the caecum, together with demethyleneberberine. Berberrubine and demethyleneberberine were not produced by intestinal cells *in vitro*, but reduced in the presence of cells, suggesting a possible utilization of these metabolites by the cells. So far, the contribution of the host in the metabolism of berberine has been studied more extensively than the contribution of the microbiota. Several hepatic CYP450 of rat or human origin were identified to be responsible for the conversion of berberine into demethyleneberberine (CYP1A2, CYP2B, CYP2D6, CYP3A1, CYP3A2 and CYP3A4) or thalifendine (CYP1A2, CYP2D6 and CYP3A4), while it was not consistent in the case of berberrubine (Liu et al., 2009; Li et al., 2011). Human hepatocytes converted berberine into jatrorrhizine, columbamine, demethyleneberberine, and to a lower degree, berberrubine, as well as associated phase II metabolites (Liu et al., 2022). It is unclear whether similar reactions occur in gut epithelial cells. In our study, demethyleneberberine was not produced by intestinal cells *in vitro*. *In vivo*, high levels of demethyleneberberine in the caecum were explained by the deconjugation of high levels of demethyleneberberine glucuronides. After oral administration of berberine in rats, few amounts of demethyleneberberine were quantified in feces (1.49%), while higher amounts of demethyleneberberine glucuronide or sulfate forms were found in the bile compared to its unconjugated form. This suggests that demethyleneberberine might not be primary produced in the gut, but rather comes from enterohepatic circulation where demethyleneberberine-glucuronide excreted from the liver via the bile is transformed to demethyleneberberine by bacterial glucuronidases. In rat intestinal microsomes, the formation of thalifendine from berberine was much greater than that of berberrubine, suggesting that thalifendine is a major metabolite in rat intestinal microsomes (Kwon et al., 2020). After oral administration of berberine in rats, excretion of berberrubine in bile (2.6%) and urine (1.2%) was much lower than in feces (18.6%), indicating that most of the berberrubine excreted in feces may be derived from the unabsorbed berberine via demethylation enzymes, present either in eukaryotic cells or in the gut microbiota (Feng et al., 2021).

Few studies investigated the role of the gut microbiota in the production of berberine metabolites. Alolga et al. (2016) showed that Chinese volunteers displayed a lower concentration of berberine in plasma and higher concentration of metabolites, including berberrubine, thalifendine, demethyleneberberine and jatrorrhizine, in feces, compared to African volunteers. These significant differences were due to variations in the gut microbial metabolism and more particularly in the abundance of four genera, i.e., *Prevotella*, *Bacteroides*, *Faecalibacterium* and *Megamonas*. The latter negatively correlate with berberine concentration in plasma, however no correlations with berberine metabolites were shown. In another study, berberine was shown to be transformed to berberrubine and thalifendine by the CYP51 enzyme, also known as sterol 14-α demethylase, a CYP450 involved in the biosynthesis of membrane sterols in all biological kingdoms from bacteria to animals. The addition of a specific inhibitor of CYP51 significantly decreased the formation of berberrubine and thalifendine *in vitro* and *in vivo*, and this was more pronounced in the gut microbiota than in the liver, with berberrubine being more affected than thalifendine. Single facultative anaerobic bacteria including *Enterococcus cloacae*, *Enterococcus faecium*, *Enterococcus faecalis* and *Staphylococcus epidermidis* expressed CYP51 and were able to generate thalifendine and, more moderately, berberrubine, from berberine, however their contribution *in vivo* was not studied (Zhang et al., 2021). CYP51 was also involved in the transformation of palmatine, another isoquinoline alkaloid and metabolite of berberine, to its demethylated products columbamine, jatrorrhizine and demethyleneberberine by the intestinal microbiota *in vitro* (He et al., 2017). Although this brings further evidence about the metabolic transformation mechanism of berberine by the gut microbiota, CYP51 is poorly characterized within bacteria. Summarized, berberrubine thus seems to mainly originate from gut microbiota metabolism and might represent a crosstalk between berberine, the gut microbiota and the host. Thalifendine and demethyleneberberine derive from both host and gut microbiota metabolism, where hepatic metabolism seems of main importance for the generation of demethyleneberberine.

Potent beneficial bacteria including acetogens demethylate berberine in the gut. Berberrubine and thalifendine were produced in intestinal segments characterized by a low level of oxygen and the presence of large bacterial communities of obligate anaerobes, including the caecum and the colon. *In vitro* and *in vivo* correlations between these berberine metabolites and bacterial abundance in the caecum revealed that berberrubine and thalifendine were mainly associated with members of the families Lachnospiraceae and Ruminococcaceae, major representatives of the bacterial community in the large intestine, including numerous SCFA-producers from *Clostridium* clusters XIVa and IV respectively. In our previous work, we showed that a high dose of in-feed berberine generated a dysbiosis in the chicken gut microbiota and decreased the relative abundance of members of the Lachnospiraceae and Ruminococcaceae family in the caecum (Dehau et al., 2023). In addition, two studies investigating the excretion of berberine metabolites in rats (thalifendine not included) showed that the total recovery of berberrubine and berberine in feces after oral administration of berberine at 48.2 mg/kg or 200 mg/kg were 18.6% and 8.4%, or 1.14% and 19.05% respectively (Ma et al., 2013; Feng et al., 2021). A higher dose of berberine likely led to a less extensive metabolism of the parent molecule and a lower content of the microbiota-derived metabolite berberrubine, that could be due to a decrease of berberrubine-producing bacteria in the gut.

The identification of specific gut bacteria that can mediate the conversion of berberine to its demethylated metabolites berberrubine and thalifendine revealed a significant role of *Blautia* spp. *in vivo*. *In vitro*, *B. coccoides* efficiently converted berberine into thalifendine, as well as did *E. limosum* to a lesser extent. *Blautia producta*, a phylogenetically close relative of *B. coccoides*, and *E. limosum* are known to demethylate dietary methyl ethers (Possemiers et al., 2008; Burapan et al., 2017; Rich et al., 2022). Both bacteria are acetogens which use corrinoid-dependent methyltransferase systems to funnel both the methyl group of this substrate and CO_2_ into the production of acetate through the Wood-Ljundahl pathway. As thalifendine positively correlated with predicted methyltransferase genes of this pathway, we suspected that *B. coccoides* may use berberine to produce acetate. Also, both berberine and thalifendine were positively associated with acetate levels *in vivo*. Previous studies have identified *Blautia* as an important genus associated with improvements of glucose and lipid homeostasis following berberine treatment in animals and humans (Li et al., 2016; Tong et al., 2018; Wu et al., 2020). *Blautia* spp. was further identified as the key bacterium mediating the anti-hypercholesterolemic action of berberine *in vivo* (Wu et al., 2020; Yang et al., 2022). Moreover, *Blautia* spp. were repetitively associated with the improvement of metabolic disorders, independently of a berberine intervention, owing to their abilities to reduce inflammatory markers and provide substrates such as acetate to butyrate-producing bacteria therefore improving the gut environment (Ozato et al., 2019; Benítez-Páez et al., 2020; Hosomi et al., 2022). Berberine, via its demethylation, might therefore stimulate acetate metabolism in *Blautia spp,* which leads to beneficial effects in the host, either direct notably on glucose and lipid homeostasis, or indirect through cross-feeding mechanisms stimulating butyrate production (Hernández et al., 2019). In addition, berberrubine modulated glucose and lipid metabolism *in vivo* (Yang et al., 2022). One could therefore suggest that the *Blautia*-mediated effects of berberine lie partly with the conversion of berberine into its demethylated metabolites, via direct bioactivity of the metabolites and/or via indirect effect of the SCFAs acetate and butyrate resulting from the acetogenesis and cross-feeding mechanisms. In our study, the butyrate-producer *Anaerostipes* was associated with thalifendine levels *in vivo*, and it was previously reported to benefit from *Blautia*-derived acetate for enhanced-butyrate formation (Bui et al., 2019). Acetogens might thus be involved in the demethylation of berberine into mainly thalifendine and might mediate the biological effects of berberine. Further research is needed to identify which enzymes are specifically involved in this transformation, as metabolic capacities across different species of the genus *Blautia* and acetogens in general vary, as well as might extend to bacteria not-commonly described as acetogens. For example, unidentified members of the *Clostridiales vadin BB60 group* were specifically associated with berberrubine. While this genus is largely uncharacterized in its metabolic role in the microbiome, we showed that multiple OTUs in it carried a methyltransferase activity potential, normally observed in acetogens. Moreover, methyltransferases can be regioselective and different methyltransferases might thus be involved in the formation of berberrubine and thalifendine respectively (Grimm et al., 2020). The identification of specific species in the gut microbiota interacting with berberine and their associated metabolomic profile appears essential to solve the mode of action of this compound. So far, metabolic interactions between berberine and *Blautia* have only been detailed in the context of metabolic disorders. Further investigation is needed to clarify whether these interactions could play a role in gut-health-promoting effects of berberine. While berberine has already attracted considerable interest over the past decades for the management of inflammatory disorders of the gastrointestinal tract, the potential of *Blautia* for this application has only been investigated recently (Mao et al., 2023).

**FIGURE 13:**
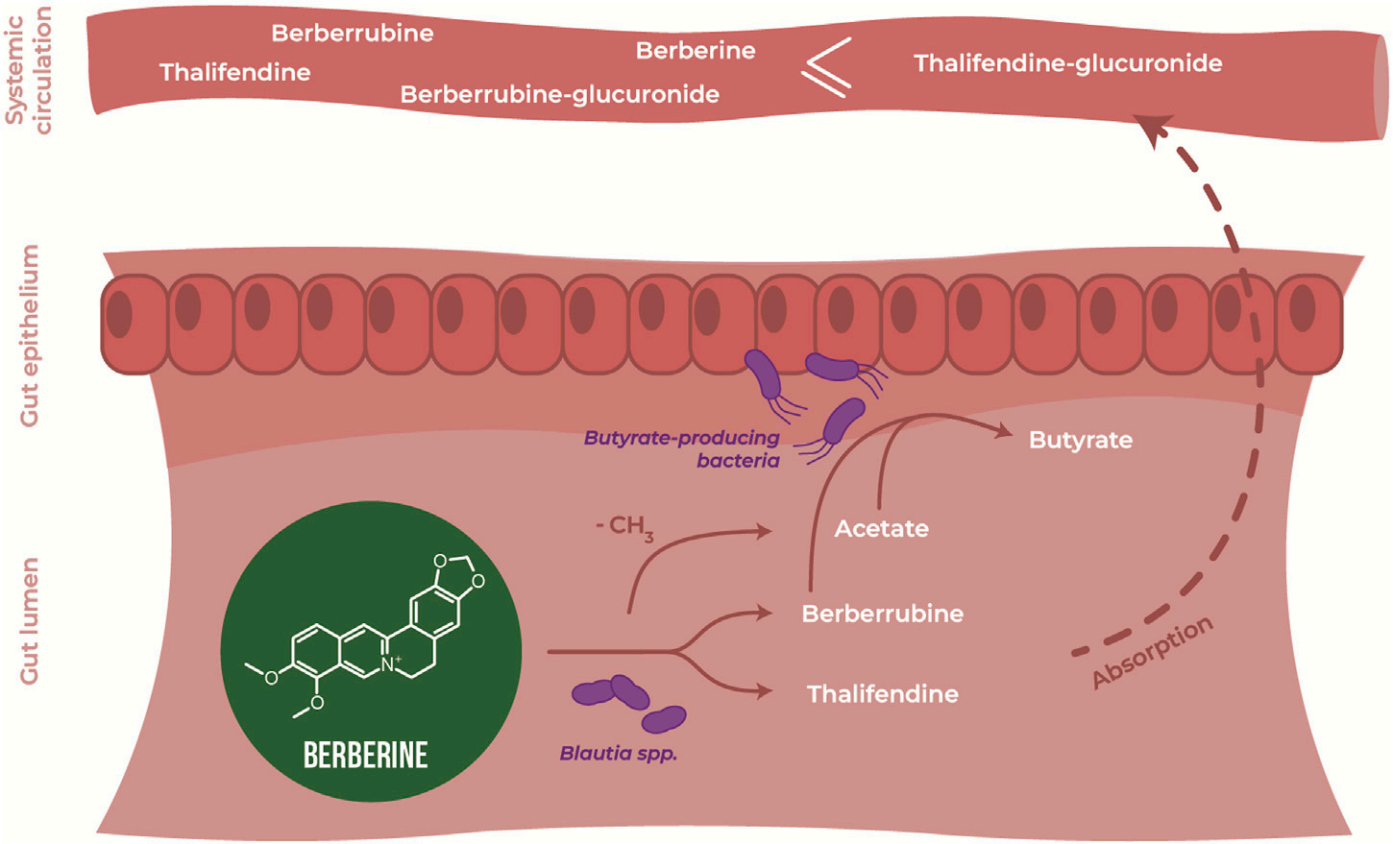
Demethylation of berberine by key gut microbes, a key step to understand biological activities of berberine. Berberine is demethylated by the gut microbiota into thalifendine and berberrubine, including bacteria of the genus *Blautia*. This conversion might drive the production of acetate, and further of butyrate by cross-feeding acetate-converting, butyrate-producing bacteria. Demethylated metabolites of berberine may also directly influence SCFA metabolism, or be further absorbed and conjugated to reach the systemic circulation and exert global effects. Gut microbiota-derived metabolites including SCFAs, thalifendine and berberrubine may be part of the mode of action of berberine *in vivo*.

In conclusion, after oral administration, the majority of berberine reaches the large intestine where the microbiota, mainly acetogenic bacteria such as *Blautia* spp., convert it to demethylated metabolites, thalifendine and berberrubine, likely to participate in its health effects, particularly by modulating SCFAs in the gut (Figure 13). In addition, these metabolites are generally better absorbed than berberine and further converted to their glucuronide forms in the liver and transported to the general circulation, where they reach high concentrations and may exert significant pharmacological actions. The gut microbiota composition therefore influences the concentration of berberine metabolites and overall pharmacological effects of berberine. The optimisation of the gut microbiota towards the increase of berberine-metabolizing species and the production of bioactive metabolites, rather than the increase of the dose of berberine used which may induce dysbiosis, appears as an interesting approach to overcome berberine’s low bioavailability and optimise its effects.

## Data Availability

The datasets presented in this study can be found in online repositories. The names of the repository/repositories and accession number(s) can be found below: NCBI BioProject (https://www.ncbi.nlm.nih.gov/bioproject/), PRJNA937446.
