# Supplementary material for: Berberine-microbiota interplay: orchestrating gut health through modulation of the gut microbiota and metabolic transformation into bioactive metabolites
Source: Front Pharmacol. 2023 Dec 7;14:1281090. doi: 10.3389/fphar.2023.1281090 (PMC10733463; doi:10.3389/fphar.2023.1281090)
Supplement: Supplementary file 1 [file DataSheet1.DOCX]

# Supplementary Methods

**Supplementary Methods S1** – Validation results of the quantification of berberine and berberine-derived metabolites in plasma and intestinal contents (ileum, caecum) by UPLC-MS/MS. The analytical method was evaluated in terms of the validation criteria described in the CVMP (EMEA/CVMP/VICH/463202/2009) and in the CHMP (EMEA/CHMP/EWP/192217/20) guidelines.

**PLASMA SAMPLES**

Quantification of all analytes in plasma was based on the internal standard berberine-d6, with thalifendine being quantified on the berberrubine calibration curve and phase II metabolites being quantified relative to the berberine calibration curve, since for these compounds no analytical reference standards were available.

*Linearity of berberine and derived metabolites in chicken plasma*

| **Compound** | **Evaluation occasion** | **a** | **b** | **r** | **GoF (%)** |
| --- | --- | --- | --- | --- | --- |
| **Berberine** | Day 1 | 0.1199 | -0.009461 | 0.9950 | 8.6 |
|  | Day 2 | 0.1192 | 0.006067 | 0.9979 | 5.9 |
|  | Day 3 | 0.1152 | -0.02617 | 0.9952 | 9.1 |
| **Demethyleneberberine** | Day 1 | 0.06053 | -0.03304 | 0.9923 | 10.6 |
|  | Day 2 | 0.06464 | -0.04756 | 0.9997 | 2.2 |
|  | Day 3 | 0.03961 | -0.03596 | 0.9959 | 8.2 |
| **Columbamine** | Day 1 | 0.08222 | -0.002341 | 0.9907 | 8.3 |
|  | Day 2 | 0.08273 | -0.0005642 | 0.9992 | 3.6 |
|  | Day 3 | 0.07026 | -0.01213 | 0.9969 | 7.3 |
| **Jatrorrhizine** | Day 1 | 0.1109 | 0.01691 | 0.9942 | 9.2 |
|  | Day 2 | 0.1096 | 0.002091 | 0.9996 | 2.5 |
|  | Day 3 | 0.09425 | -0.009720 | 0.9969 | 7.3 |
| **Berberrubine** | Day 1 | 0.2765 | -0.03025 | 0.9955 | 8.1 |
|  | Day 2 | 0.2639 | 0.01340 | 0.9989 | 4.4 |
|  | Day 3 | 0.2346 | -0.04800 | 0.9994 | 3.1 |
| **Dihydroberberine** | Day 1 | 0.1078 | 0.01401 | 0.9949 | 8.9 |
|  | Day 2 | 0.09008 | 0.005762 | 0.9956 | 8.7 |
|  | Day 3 | 0.07714 | -0.01574 | 0.9965 | 7.8 |
| **Palmatine** | Day 1 | 0.08584 | -0.01047 | 0.9944 | 9.1 |
|  | Day 2 | 0.08312 | -0.006986 | 0.9982 | 5.6 |
|  | Day 3 | 0.08060 | -0.01519 | 0.9950 | 9.2 |

Matrix-matched calibration curves were prepared on three different analysis days. Calibration curves were best described with a linear calibration model y= ax + b, using a 1/x2 fit weighting, resulting as such in the lowest goodness-of-fit coefficient (GoF) %. As can be seen, the correlation coefficient (r) was above the 0.99 criterion, while the GoF (%) was below the 20 % criterion for all components.

*Within-day and between-day accuracy and precision evaluation of berberine and phase I metabolites in plasma at 0.1, 1, 10, and 100 ng/ml levels.*

| Compound | Time | Level 1: 0.1 ng/ml | Level 2: 1 ng/ml | Level 3: 10 ng/ml | Level 4: 100 ng/ml |
| --- | --- | --- | --- | --- | --- |
| Berberine | **Day 1** | 0.075 ± 0.010 (acc -25.0%)  RSD 13.7% | 0.98 ± 0.04 (acc -2.3%)  RSD 4.3% | 8.9 ± 0.3 (acc -10.9%)  RSD 3.8% | 102.8 ± 1.7 (acc +2.8%)  RSD 1.6% |
|  | **Day 2** | 0.090 ± 0.026 (acc -10.0%)^1^  RSD 29.4% | 0.90 ± 0.05 (acc -9.7%)  RSD 5.8% | 9.6 ± 0.4 (acc -3.6%)  RSD 4.6% | 108.3 ± 3.4 (acc +8.3%)  RSD 3.2% |
|  | **Day 3** | 0.074 ± 0.012 (acc -26.0%)  RSD 16.5% | 1.08 ± 0.05 (acc +8.3%)  RSD 5.0% | 8.4 ± 0.2 (acc -16.5%)  RSD 2.3% | 107.3 ± 1.4 (acc +7.3%)  RSD 1.3% |
|  | **Between-day** | 0.079 ± 0.018 (acc -19.0%)  RSD 22.2% | 0.99 ± 0.09 (acc -1.2%)  RSD 9.0% | 9.0 ± 0.6 (acc -10.3%)  RSD 7.0% | 106.1 ± 3.3 (acc +6.1%)  RSD 3.1% |
| Demethylene-berberine | **Day 1** | 0.10 ± 0.01 (acc -2.6%)^2^  RSD 12.3% | 0.99 ± 0.06 (acc -1.5%)  RSD 6.2% | 8.8 ± 0.5 (acc -12.1%)  RSD 5.4% | 93.5± 2.3 (acc -6.6%)  RSD 2.5% |
|  | **Day 2** | 0.068 ± 0.006 (acc -31.8%)^2^  RSD 8.3% | 1.04 ± 0.05 (acc +3.5%)  RSD 5.1% | 10.0 ± 0.9 (acc -0.3%)  RSD 8.9% | 88.1 ± 2.0 (acc -11.9%)  RSD 2.3% |
|  | **Day 3** | 0.090 ± 0.005 (acc -9.8%)  RSD 5.0% | 0.99 ± 0.03 (acc -0.9%)  RSD 2.6% | 9.0 ± 0.1 (acc -10.1%)  RSD 1.4% | 108.4 ± 4.2 (acc +8.4%)  RSD 3.8% |
|  | **Between-day** | 0.086 ± 0.014 (acc -13.1%)  RSD 16.9% | 1.00 ± 0.05 (acc +0.4%)  RSD 5.1% | 9.2 ± 0.8 (acc -7.5%)  RSD 8.3% | 96.6 ± 9.3 (acc -3.4%)  RSD 9.6% |
| Columbamine | **Day 1** | 0.092 ± 0.013 (acc -8.2%)^3^  RSD 13.8% | 0.91 ± 0.05 (acc -9.3%)  RSD 5.6% | 8.7 ± 0.2 (acc -12.9%)  RSD 2.8% | 99.8 ± 1.8 (acc -0.2%)  RSD 1.9% |
|  | **Day 2** | 0.088 ± 0.009 (acc -12.0%)^3^  RSD 9.8% | 0.91 ± 0.02 (acc -9.5%)  RSD 2.6% | 9.2 ± 0.7 (acc -8.0%)  RSD 7.1% | 101.5 ± 3.0 (acc +1.5%)  RSD 3.0% |
|  | **Day 3** | 0.11 ± 0.009 (acc +10.8%)  RSD 7.7% | 1.00 ± 0.05 (acc +0.1%)  RSD 4.7% | 8.1 ± 0.1 (acc -19.5%)  RSD 1.8% | 103.8 ± 3.1 (acc +3.8%)  RSD 3.0% |
|  | **Between-day** | 0.098 ± 0.014 (acc -2.0%)  RSD 14.4% | 0.94 ± 0.06 (acc -6.3%)  RSD 6.5% | 8.7 ± 0.6 (acc -13.5%)  RSD 7.1% | 101.7 ± 3.1 (acc +1.7%)  RSD 3.0% |
| Jatrorrhizine | **Day 1** | 0.10 ± 0.04 (acc +0.8%)^4^  RSD 35.2% | 0.91 ± 0.17 (acc -9.0%)^4^  RSD 18.5% | 8.7 ± 0.4 (acc -12.5%)  RSD 4.5% | 98.4 ± 1.9 (acc -1.6%)  RSD 1.9 % |
|  | **Day 2** | 0.075 ± 0.009 (acc -25.2%)^4^  RSD 12.2% | 0.96 ± 0.09 (acc -4.3%)  RSD 9.7% | 9.6 ± 0.8 (acc -4.3%)  RSD 8.2% | 103.2 ± 0.9 (acc +3.2%)  RSD 0.9% |
|  | **Day 3** | 0.091 ± 0.010 (acc -8.8%)  RSD 11.1% | 0.96 ± 0.02 (acc -4.5%)  RSD 2.5% | 8.2 ± 0.2 (acc -17.6%)  RSD 3.0% | 103.5 ± 3.1 (acc +3.5%)  RSD 3.0% |
|  | **Between-day** | 0.089 ± 0.023 (acc -9.9%)  RSD 25.3% | 0.94 ± 0.10 (acc -5.6%)  RSD 10.1% | 8.9 ± 0.7 (acc -11.5%)  RSD 8.4% | 101.7 ± 3.1 (acc +1.7%)  RSD 3.1% |
| Berberrubine | **Day 1** | 0.072 ± 0.010 (acc -27.8%)  RSD 14.0% | 0.98 ± 0.01 (acc -1.8%)  RSD 1.4% | 9.1 ± 0.3 (acc -9.0%)  RSD 3.4% | 95.4 ± 2.2 (acc -4.6%)  RSD 2.3% |
|  | **Day 2** | 0.079 ± 0.008 (acc -20.8%)  RSD 10.6% | 0.84 ± 0.04 (acc -15.8%)  RSD 5.1% | 9.0 ± 0.4 (acc -10.5%)  RSD 4.0% | 96.2 ± 6.8 (acc -3.8%)  RSD 7.1% |
|  | **Day 3** | 0.12 ± 0.01 (acc +16.0%)  RSD 10.3% | 0.96 ± 0.02 (acc -3.7%)  RSD 2.3% | 8.9 ± 0.2 (acc -11.0%)  RSD 2.6% | 98.1 ± 2.7 (acc -1.9%)  RSD 2.8% |
|  | **Between-day** | 0.089 ± 0.022 (acc -9.9%)  RSD 24.7% | 0.93 ± 0.07 (acc -7.1%)  RSD 7.5% | 9.0 ± 0.3 (acc -10.2%)  RSD 3.3% | 96.6 ± 4.3 (acc -3.4%)  RSD 4.5% |
| Dihydro-  berberine | **Day 1** | 0.091 ± 0.013 (acc -9.3%)  RSD 14.5% | 0.86 ± 0.08 (acc -14.2%)  RSD 9.6% | 9.5 ± 0.6 (acc -4.8%)  RSD 5.9% | 99.5 ± 2.3 (acc -0.5%)  RSD 2.3% |
|  | **Day 2** | 0.085 ± 0.012 (acc -14.8%)  RSD 13.6% | 0.93 ± 0.17 (acc -7.5%)  RSD 18.9% | 9.7 ± 1.6 (acc -3.1%)  RSD 16.5% | 101.0 ± 11.8 (acc +1.0%)  RSD 11.7% |
|  | **Day 3** | 0.10 ± 0.01 (acc +3.5%)  RSD 8.1% | 1.00 ± 0.04 (acc 0.0%)  RSD 4.0% | 8.7 ± 0.4 (acc -12.7%)  RSD 4.0% | 98.7 ± 3.6 (acc -1.3%)  RSD 3.6% |
|  | **Between-day** | 0.093 ± 0.013 (acc -6.3%)  RSD 14.1% | 0.93 ± 0.12 (acc -7.2%)  RSD 13.2 % | 9.3 ± 1.0 (acc -6.8%)  RSD 11.1% | 99.7 ± 6.9 (acc -0.3%)  RSD 6.9% |
| Palmatine | **Day 1** | 0.077 ± 0.013 (acc -22.7%)  RSD 17.2% | 0.99 ± 0.03 (acc -0.8%)  RSD 2.8% | 8.5 ± 0.3 (acc -15.5%)  RSD 3.9% | 102.4 ± 1.9 (acc +2.4%)  RSD 1.8% |
|  | **Day 2** | 0.095 ± 0.009 (acc -5.5%)  RSD 10.0% | 0.94 ± 0.03 (acc -6.3%)  RSD 2.8% | 9.3 ± 0.3 (acc -6.6%)  RSD 3.6% | 106.7 ± 1.4 (acc +6.7%)  RSD 1.3% |
|  | **Day 3** | 0.10 ± 0.01 (acc -2.5%)  RSD 12.6% | 0.96 ± 0.06 (acc -3.5%)  RSD 5.8% | 8.0 ± 0.2 (acc -20.2%)  RSD 2.9 % | 108.9 ± 1.5 (acc +8.9%)  RSD 1.4% |
|  | **Between-day** | 0.090 ± 0.014 (acc -9.3%)  RSD 16.0% | 0.96 ± 0.04 (acc -3.6%)  RSD 4.5% | 8.6 ± 0.6 (acc -14.1%)  RSD 7.5% | 106.0 ± 3.2 (acc +6.0 %)  RSD 3.0% |

At each level evaluated, 0.1, 1, 10, and 100 ng/ml, n = 6 independent measurements were performed at each of three analysis days. Mean values ± SD are given, as well as accuracy (% deviation from nominal value), and precision (as % RSD). A limited number of outlier values was excluded.^1^ for berberine: 0.1 ng/ml: repl-03 value validation day 2 (0.17 ng/ml); ^2^ for demethyleneberberine: 0.1 ng/ml: repl-01 value validation day 1 (0.15 ng/ml), repl-05 value day 2 (0.13 ng/ml); ^3^ for columbamine: 0.1 ng/ml level: repl-02 value (0.30 ng/ml) and repl-01 value (0.13 ng/ml) at validation day 1 and 2 resp.; ^4^ for jatrorrhizine: validation day 1: at 0.1 ng/ml, repl-01 value excluded at 0.46 ng/ml, at 1 ng/ml, repl-04 and repl-05 values excluded at 4.8 and 2.9 ng/ml resp., validation day 2: at 0.1 ng/ml, repl-01 value excluded at 0.12 ng/ml.

Tolerances were as follows for within-day accuracy: -20% to +10% at 10 and 100 ng/ml levels, and -50 to +20 % at 0.1 and 1 ng/ml levels (Heitzman, 1994; EU legislation, 1991, 2003, 2004). Maximum tolerance for within-day precision was concentration dependent: 10%, 15%, 25%, and 30% at the 100, 10, 1, and 0.1 ng/ml respectively (VICH topic GL49, 2016). Criteria were met for within-day accuracy and precision the compounds berberine, demethyleneberberine, columbamine, and berberrubine. Borderline failing results were observed for jatrorrhizine (validation day 1, RSD 35.2% at 0.1 ng/ml level), dihydroberberine (validation day 1, RSD 11.7% at 100 ng/ml level; validation day 2, RSD 16.5 % at 10 ng/ml level, and palmatine (validation day 3, -20.2 % accuracy at 10 ng/ml level).

Related to between-day accuracy and precision, the above-mentioned criteria were met for all components at each level tested, while between-day criteria for precision are acutally even larger, as calculated according to the Horwitz equation: 22.6%, 32.0%, 45.3%, and 64.0% at the 100, 10, 1, and 0.1 ng/ml respectively (Heitzman, 1994; EU legislation, 2003, 2004).

*Stability of berberine and phase I metabolites in processed plasma sample extract at the 1, 10, and 100 ng/ml levels, stored at 10°C in autosampler for 24h.*

| Compound | Level 1: 1 ng/ml | Level 2: 10 ng/ml | Level 3: 100 ng/ml |
| --- | --- | --- | --- |
| Berberine | 0.89 ± 0.09 (acc -11.3%)  RSD 9.7% | 9.1 ± 0.3 (acc -9.2%)  RSD 3.7% | 105.1 ± 2.4 (acc +5.1%)  RSD 2.3% |
| Demethyleneberberine | 1.3 ± 0.1 (acc +26.7%)  RSD 10.6% | 12.2 ± 0.8 (acc +12.2%)  RSD 6.7% | 102.8 ± 9.7 (acc +2.8%)  RSD 9.4% |
| Columbamine | 0.91 ± 0.03 (acc -9.3%)  RSD 3.5% | 8.7 ± 0.4 (acc -13.0%)  RSD 4.3% | 101.4 ± 5.1 (acc +1.4%)  RSD 5.1% |
| Jatrorrhizine | 1.01 ± 0.4 (acc +1.0%)  RSD 4.2% | 9.5 ± 0.4 (acc -4.7%)  RSD 4.2% | 105.8 ± 8.1 (acc +5.8%)  RSD 7.7% |
| Berberrubine | 0.78 ±0.06 (acc -22.0%)  RSD 7.1% | 9.2 ± 0.4 (acc -8.4%)  RSD 4.4% | 98.6 ± 3.4 (acc -1.4%)  RSD 3.4% |
| Dihydroberberine | 1.01 ± 0.04 (acc +1.0%)  RSD 4.2% | 11.4 ± 0.6 (acc +14.2%)  RSD 5.2% | 113.5 ± 5.1 (acc +13.5%)  RSD 4.5% |
| Palmatine | 0.95 ± 0.05 (acc -4.7%)  RSD 5.2% | 8.5 ± 0.6 (acc -14.5%)  RSD 6.4% | 105.6 ± 4.2 (acc +5.6%)  RSD 4.0% |

At each level evaluated, 1, 10 and 100 ng/mL, n = 3 replicates were compared to a freshly prepared matrix-matched calibration curve.

In all cases, the criteria for precision were met (see section on accuracy and precision for criteria). The same was true for accuracy, except for demethyleneberberine for which the concentration found was above the upper limit, resp. + 20 % and + 10 % at the lower 1 ng/ml and 10 ng/ml levels. Also, for dihydroberberine, for accuracy, borderline failing results should be noted: +14.2% and +13.5 % at the 10 and 100 ng/ml levels respectively, indicating however that the addition of ascorbic acid during sample preparation effectively succeeded in stabilizing this unstable compound.

**INTESTINAL CONTENT SAMPLES – 1/5 diluted sample extracts**

Quantification of all analytes in intestinal contents was based on the internal standard tetrahydropalmatine for 1/5 diluted samples, which were injected for quantification of analytes present at lower levels. Thalifendine was quantified on the berberrubine calibration curve and phase II metabolites were quantified relative to the berberine calibration curve, since no analytical reference standards were available for these compounds. Validation experiments were performed in ileum and caecum, each 2 days, while cross-validation experiments were also performed.

*Linearity of berberine and derived metabolites in chicken intestinal content (day 1,2: ileum; day 3,4: caecum)*

| **Compound** | **Evaluation occasion** | **a** | **b** | **c** | **R^2^** | **GoF (%)** |
| --- | --- | --- | --- | --- | --- | --- |
| **Berberine** | Day 1 | -5.475e-8 | 0.003354 | -0.004580 | 0.9977 | 3.7 |
|  | Day 2 | -1.038e-7 | 0.003250 | -0.009341 | 0.9980 | 3.5 |
|  | Day 3 | -5.048e-8 | 0.003047 | 0.0001688 | 0.9968 | 4.5 |
|  | Day 4 | -1.052e-8 | 0.002974 | -0.01018 | 0.9996 | 1.7 |
| **Demethyleneberberine** | Day 1 | -7.496e-8 | 0.002167 | 0.002067 | 0.9994 | 1.9 |
|  | Day 2 | -1.100e-7 | 0.002104 | -0.009537 | 0.9986 | 3.0 |
|  | Day 3 | -8.805e-8 | 0.001993 | -0.008288 | 0.9966 | 4.9 |
|  | Day 4 | -6.560-8 | 0.001911 | 0.01072 | 0.9988 | 2.8 |
| **Columbamine** | Day 1 | -4.535e-8 | 0.002016 | 0.0009327 | 0.9987 | 2.8 |
|  | Day 2 | -7.315e-8 | 0.002002 | 0.008939 | 0.9982 | 3.4 |
|  | Day 3 | -5.964e-8 | 0.001862 | -0.004771 | 0.9947 | 5.8 |
|  | Day 4 | -2.151e-8 | 0.001789 | -0.008370 | 0.9987 | 2.9 |
| **Jatrorrhizine** | Day 1 | -3.868e-8 | 0.001991 | 0.1003776 | 0.9993 | 2.0 |
|  | Day 2 | -6.923e-8 | 0.001989 | -0.003432 | 0.9981 | 3.4 |
|  | Day 3 | -5.474e-8 | 0.001853 | -0.0004837 | 0.9949 | 5.6 |
|  | Day 4 | -1.317e-8 | 0.001793 | -0.008549 | 0.9996 | 1.7 |
| **Berberrubine** | Day 1 | -1.643e-7 | 0.006676 | -0.007480 | 0.9933 | 6.2 |
|  | Day 2 | -2.548e-7 | 0.006459 | -0.05641 | 0.9981 | 3.5 |
|  | Day 3 | -1.318e-7 | 0.005594 | -0.01213 | 0.9925 | 6.9 |
|  | Day 4 | -7.511e-8 | 0.005469 | -0.03530 | 0.9981 | 3.5 |
| **Dihydroberberine** | Day 1 | 1.284e-8 | 0.001456 | 0.008398 | 0.9980 | 3.5 |
|  | Day 2 | -5.046e-9 | 0.001482 | -0.008722 | 0.9991 | 2.4 |
|  | Day 3 | -1.943e-8 | 0.001357 | -0.005187 | 0.9981 | 3.5 |
|  | Day 4 | 1.393e-9 | 0.001342 | -0.01435 | 0.9989 | 2.7 |
| **Palmatine** | Day 1 | -3.484e-8 | 0.002515 | -0.001354 | 0.9989 | 2.6 |
|  | Day 2 | -8.454e-8 | 0.002507 | -0.01374 | 0.9980 | 3.6 |
|  | Day 3 | -6.513e-8 | 0.002329 | -0.008129 | 0.9965 | 4.7 |
|  | Day 4 | -1.628e-8 | 0.002210 | -0.01051 | 0.9991 | 2.4 |
| **Oxyberberine** | Day 1 | -9.223e-8 | 0.005822 | 0.03513 | 0.9541 | 16.2 |
|  | Day 2 | -2.334e-7 | 0.007016 | -0.1667 | 0.9993 | 2.0 |
|  | Day 3 | 9.244e-8 | 0.005157 | -0.1316 | 0.9832 | 10.4 |
|  | Day 4 | -2.559e-8 | 0.007317 | -0.3489 | 0.9469 | 19.5 |

Matrix-matched calibration curves were prepared on two different analysis days for both ileum (day 1,2) and caecum (day 3,4). Results concern quantification based on the internal standard tetrahydropalmatine. Calibration curves were best described with a kwadratic calibration model y= ax2 + bx + c, using a 1/x2 fit weighting, resulting as such in the lowest goodness-of-fit coefficient (GoF) %. As can be seen, the coefficient of determination (R^2^) was above the 0.99 criterion, while the GoF (%) was below the 10 % criterion for all components, except for oxyberberine.

*Within-day and between-day accuracy and precision evaluation of berberine and phase I metabolites in ileum (day 1, 2) and caecum (day 3, 4) contents at 62.5, 500, and 5000 ng/g levels*

| Compound | Time | Level 1: 62.5 ng/g | Level 2: 500 ng/g | Level 3: 5000 ng/g |
| --- | --- | --- | --- | --- |
| Berberine | **Day 1** | 63.8 ± 2.9 (acc +2.1%)  RSD 4.5% | 514.0 ± 18.4 (acc +2.8%)  RSD 3.6% | 4934.1 ± 57.5 (acc -1.3%)  RSD 1.2% |
|  | **Day 2** | 60.1 ± 2.0 (acc -3.8%)  RSD 3.3% | 500.6 ± 18.3 (acc +0.1%)  RSD 3.7% | 5225.1 ± 119.8 (acc +4.5%)  RSD 2.3% |
|  | **Day 3** | 62.8 ± 1.6 (acc +0.6%)  RSD 2.5% | 501.8 ± 6.5 (acc +0.4%)  RSD 1.3% | 5147.7 ± 92.7 (acc +3.0%)  RSD 1.8% |
|  | **Day 4** | 62.6 ± 1.2 (acc +0.2%)  RSD 1.9% | 497.8 ± 13.6 (acc -0.4%)  RSD 2.7% | 4914.2 ± 79.7 (acc -1.7%)  RSD 1.6% |
| Demethylene-  berberine | **Day 1** | 60.2 ± 1.7 (acc -3.7%)  RSD 2.9% | 510.2 ± 5.7 (acc +2.0%)  RSD 1.1% | 4956.9 ± 124.6 (acc -0.9%)  RSD 2.5% |
|  | **Day 2** | 63.6 ± 1.9 (acc +1.8%)  RSD 3.0% | 502.2 ± 21.6 (acc +0.4%)  RSD 4.3% | 5245.2 ± 159.6 (acc +4.9%)  RSD 3.0% |
|  | **Day 3** | 64.1 ± 1.2 (acc +2.5%)  RSD 1.9% | 488.4 ± 5.8 (acc -2.3%)  RSD 1.2% | 5178.1 ± 134.1 (acc +3.6%)  RSD 2.6% |
|  | **Day 4** | 63.8 ± 1.9 (acc +2.0%)  RSD 2.9% | 491.4 ± 15.4 (acc -1.7%)  RSD 3.1% | 4881.1 ± 150.6 (acc -2.4%)  RSD 3.1% |
| Columbamine | **Day 1** | 62.1 ± 3.1 (acc -0.7%)  RSD 4.9% | 509.1 ± 7.0 (acc +1.8%)  RSD 1.4% | 4762.4 ± 152.7 (acc -4.8%)  RSD 3.2% |
|  | **Day 2** | 62.4 ± 1.1 (acc -0.2%)  RSD 1.8% | 497.7 ± 18.0 (acc -0.5%)  RSD 3.6% | 5102.2 ± 176.2 (acc +2.0%)  RSD 3.5% |
|  | **Day 3** | 62.6 ± 1.7 (acc +0.2%)  RSD 2.7% | 505.2 ± 9.3 (acc +1.0%)  RSD 1.8% | 5273.2 ± 113.3 (acc +5.5%)  RSD 2.1% |
|  | **Day 4** | 61.9 ± 2.8 (acc -0.9%)  RSD 4.6% | 495.2 ± 17.2 (acc -1.0%)  RSD 3.5% | 4732.6 ± 117.7 (acc -5.3%)  RSD 2.5% |
| Jatrorrhizine | **Day 1** | 61.5 ± 1.8 (acc -1.5%)  RSD 2.9% | 513.3 ± 5.2 (acc +2.7%)  RSD 1.0% | 4768.0 ± 98.1 (acc -4.6%)  RSD 2.1% |
|  | **Day 2** | 60.6 ± 0.7 (acc -3.1%)  RSD 1.1% | 496.7 ± 18.8 (acc -0.7%)  RSD 3.8% | 5077.4 ± 138.2 (acc +1.5%)  RSD 2.7% |
|  | **Day 3** | 61.3 ± 1.0 (acc -1.9%)  RSD 1.7% | 495.3 ± 8.7 (acc -0.9%)  RSD 1.8% | 5241.1 ± 123.4 (acc +4.8%)  RSD 2.4% |
|  | **Day 4** | 63.7 ± 2.5 (acc +1.9%)  RSD 3.9% | 491.7 ± 19.5 (acc -1.7%)  RSD 4.0% | 4725.4 ± 116.0 (acc -5.5%)  RSD 2.5% |
| Berberrubine | **Day 1** | 61.4 ± 3.6 (acc -1.8%)  RSD 5.9% | 513.4 ± 25.4 (acc +2.7%)  RSD 4.9% | 4885.5 ± 80.7 (acc -2.3%)  RSD 1.7% |
|  | **Day 2** | 62.5 ± 2.2 (acc 0.0%)  RSD 3.6% | 500.9 ± 25.1 (acc +0.2%)  RSD 5.0% | 5031.8 ± 117.1 (acc +0.6%)  RSD 2.3% |
|  | **Day 3** | 64.7 ± 1.8 (acc +3.5%)  RSD 2.8% | 504.5 ± 8.5 (acc +0.9%)  RSD 1.7% | 5182.8 ± 56.5 (acc +3.7%)  RSD 1.1% |
|  | **Day 4** | 62.3 ± 2.9 (acc -0.4%)  RSD 4.6% | 499.5 ± 14.9 (acc -0.1%)  RSD 3.0% | 4897 ± 67.4 (acc -2.1%)  RSD 1.4% |
| Dihydro-  berberine | **Day 1** | 55.8 ± 3.7 (acc -10.7%)  RSD 6.7% | 500.8 ± 8.9 (acc +0.2%)  RSD 1.8% | 4765.4 ± 163.5 (acc -4.7%)  RSD 3.4% |
|  | **Day 2** | 64.9 ± 1.6 (acc +3.9%)  RSD 2.5% | 508.8 ± 12.6 (acc +1.8%)  RSD 2.5% | 4978.5 ± 103.7 (acc -0.4%)  RSD 2.1% |
|  | **Day 3** | 62.4 ± 1.5 (acc -0.2%)  RSD 2.5% | 503.1 ± 9.2 (acc +0.6%)  RSD 1.8% | 5240.3 ± 67.2 (acc +4.8%)  RSD 1.3% |
|  | **Day 4** | 66.6 ± 2.5 (acc +6.5%)  RSD 3.8% | 503.2 ± 18.0 (acc +0.6%)  RSD 3.6% | 4671.9 ± 135.1 (acc -6.6%)  RSD 2.9% |
| Palmatine | **Day 1** | 61.7 ± 3.0 (acc -1.3%)  RSD 4.8% | 506.6 ± 7.4 (acc +1.3%)  RSD 1.5% | 4913.5 ± 104.8 (acc -1.7%)  RSD 2.1% |
|  | **Day 2** | 62.5 ± 1.7 (acc 0.0%)  RSD 2.7% | 496.5 ± 13.5 (acc -0.7%)  RSD 2.7% | 5155.7 ± 144.5 (acc +3.1%)  RSD 2.8% |
|  | **Day 3** | 63.2 ± 1.1 (acc +1.1%)  RSD 1.8% | 481.8 ± 10.8 (acc -3.6%)  RSD 2.2% | 5140.6 ± 91.3 (acc +2.8%)  RSD 1.8% |
|  | **Day 4** | 62.7 ± 2.5 (acc +0.3%)  RSD 3.9% | 491.1 ± 15.1 (acc -1.8%)  RSD 3.1% | 4830.7 ± 82.4 (acc -3.4%)  RSD 1.7% |
| Oxyberberine | **Day 1** | 52.9 ± 3.1 (acc -15.3%)^1^  RSD 5.9% | 600.0 ± 51.9 (acc +20.0%)^1^  RSD 8.6% | 4978.6 ± 305.6 (acc -0.4%)  RSD 6.1% |
|  | **Day 2** | 66.3 ± 3.5 (acc +6.0%)  RSD 5.3% | 460.6 ± 30.2 (acc -7.9%)^2^  RSD 6.5% | 4387.6 ± 169.5 (acc -12.2%)  RSD 3.9% |
|  | **Day 3** | 66.4 ± 2.3 (acc +6.3%)  RSD 3.4% | 482.3 ± 64.8 (acc -3.5%)  RSD 13.4% | 4979.2 ± 165.4 (acc -0.4%)  RSD 3.3% |
|  | **Day 4** | 82.5 ± 6.4 (acc +32.0%)  RSD 7.7% | 576.1 ± 93.8 (acc +15.2%)  RSD 16.3% | 4354.4 ± 352.7 (acc -12.9%)  RSD 8.1% |

At each level evaluated, 62.5, 500, and 5000 ng/ng, n = 6 independent measurements were performed at each of 4 analysis days, in ileum for day 1 and day 2, and in caecum for day 3 and day 4. Mean values ± SD are given, as well as accuracy (% deviation from nominal value), and precision (as % RSD). Results concern quantification based on the internal standard tetrahydropalmatine. A limited number of outlier values had to be excluded for the component oxyberberine.^1^: validation day 1: 62.5 and 500 ng/g levels: repl-04 values validation day 1 (resp. 127.0 and 1340.5 ng/g); ^2^: validation day 2: repl-01 and repl-03 values for level 500 ng/g (814.7 and 806.7 ng/g resp.).

Tolerances for within-day accuracy was -20% to +10% at all levels tested (Heitzman, 1994; EU legislation, 1991, 2003, 2004). Maximum tolerance for within-day precision was concentration dependent: 10% at the 500, and 5000 ng/g levels, and 15 % at the 62.5 ng/g level (VICH topic GL49, 2016). Criteria were met for within-day accuracy and precision for all compounds, except oxyberberine. For the latter compound, some outlier values were observed, which were excluded at evaluation of accuracy and precision. Nevertheless, criteria for within-day accuracy and precision were not always fulfilled.

Between-day accuracy and precision evaluation require three validation days under the same circumstances, while in this particular context even four validation days were performed, but divided in two validation days related to different intestinal segments. On the other hand, without any doubt, the good within-day accuracy and precision observed over the four validation days suggest good in between-day performance of the method as well for all components involved, except oxyberberine where some more deviation and scatter of measured levels might be expected.

*Stability of berberine and phase I metabolites in processed intestinal content sample extract at the 62.5, 500, and 5000 ng/g levels, stored at 10°C in autosampler for 24h*

| ILEUM | | | |
| --- | --- | --- | --- |
| Compound | **Level 1: 62.5 ng/g** | **Level 2: 500 ng/g** | **Level 3: 5000 ng/g** |
| Berberine | 64.9 ± 1.1 (acc +3.8%)  RSD 1.7% | 503.3 ± 15.7 (acc +0.7%)  RSD 3.1% | 5598.9 ± 96.6 (acc +12.0%)  RSD 1.7% |
| Demethyleneberberine | 67.2 ± 2.4 (acc +7.6%)  RSD 3.6% | 517.5 ± 16.7 (acc +3.5%)  RSD 3.2% | 5791.0 ± 110.9 (acc +15.8%)  RSD 1.9% |
| Columbamine | 65.6 ± 1.2 (acc +5.0%)  RSD 1.8% | 509.6 ± 12.0 (acc +1.9%)  RSD 2.4% | 5239.5 ± 276.4 (acc +4.8%)  RSD 5.3% |
| Jatrorrhizine | 63.5 ± 1.1 (acc +1.7%)  RSD 1.8% | 505.8 ± 13.1 (acc +1.2%)  RSD 2.6% | 5158.5 ± 354.6 (acc +3.2%)  RSD 6.9% |
| Berberrubine | 69.0 ± 2.3 (acc +10.4%)  RSD 3.3% | 507.3 ± 17.7 (acc +1.5%)  RSD 3.5% | 5480.7 ± 112.7 (acc +9.6%)  RSD 2.1% |
| Dihydroberberine | 68.0 ± 3.0 (acc +8.8%)  RSD 4.3% | 494.2 ± 17.2 (acc -1.2%)  RSD 3.5% | 4797.1 ± 138.0 (acc -4.1%)  RSD 2.9% |
| Palmatine | 65.7 ± 2.5 (acc +5.1%)  RSD 3.9% | 497.5 ± 14.4 (acc -0.5%)  RSD 2.9% | 5458.7 ± 127.5 (acc +9.2%)  RSD 2.3% |
| Oxyberberine | 70.5 ± 1.2 (acc +12.9%)  RSD 1.7% | 472.8 ± 15.3 (acc -5.4%)  RSD 3.2% | 4393.4 ± 136.6 (acc -12.1%)  RSD 3.1% |
| CAECUM | | | |
| Compound | **Level 1: 62.5 ng/g** | **Level 2: 500 ng/g** | **Level 3: 5000 ng/g** |
| Berberine | 61.8 ± 0.7 (acc -1.1%)  RSD 1.1% | 473.1 ± 5.5 (acc -5.4%)  RSD 1.2% | 4615.5 ± 72.6 (acc -7.7%)  RSD 1.6% |
| Demethyleneberberine | 63.1 ± 1.8 (acc +1.0%)  RSD 2.8% | 468.1 ± 7.8 (acc -6.4%)  RSD 1.7% | 4562.9 ± 110.6 (acc -8.7%)  RSD 2.4% |
| Columbamine | 63.4 ± 3.2 (acc +1.4%)  RSD 5.0% | 469.2 ± 4.1 (acc -6.2%)  RSD 0.9% | 4455.6 ± 98.7 (acc -10.9%)  RSD 2.2% |
| Jatrorrhizine | 61.8 ± 1.6 (acc -1.1%)  RSD 2.5% | 464.8 ± 3.5 (acc -7.0%)  RSD 0.7% | 4431.1 ± 92.5 (acc -11.4%)  RSD 2.1% |
| Berberrubine | 64.8 ± 2.3 (acc +3.7%)  RSD 3.6% | 470.0 ± 8.0 (acc -6.0%)  RSD 1.7% | 4582.5 ± 77.0 (acc -8.3%)  RSD 1.7% |
| Dihydroberberine | 69.2 ± 2.8 (acc +10.7%)  RSD 4.0% | 480.3 ± 11.1 (acc -3.9%)  RSD 2.3% | 4628.9 ± 107.8 (acc -7.4%)  RSD 2.3% |
| Palmatine | 63.2 ± 2.7 (acc +1.2%)  RSD 4.3% | 463.3 ± 13.4 (acc -7.3%)  RSD 2.9% | 4513.7 ± 95.8 (acc -9.7%)  RSD 2.1% |
| Oxyberberine | 79.3 ±3.0 (acc +26.9%)  RSD 3.8% | 433.2 ± 55.2 (acc -13.4%)  RSD 12.7% | 3997.4 ± 236.8 (acc -20.1%)  RSD 5.9% |

At each level, n = 3 replicates were compared to a freshly prepared matrix-matched calibration curve. Results concern quantification based on the internal standard tetrahydropalmatine.

For all components, except oxyberberine, the criteria for accuracy and precision were met (see section on accuracy and precision for criteria) both in ileum and in caecum, except for some borderline failing results: at 62.5 ng/g level, for dihydroberberine (+10.7% in caecum) and berberrubine (+10.4% in ileum), and for demethyleneberberine at the 5000 ng/g level (+15.8%). For oxyberberine on the other hand, results were found to be less consistent, which is not surprisingly, since within-day accuracy and precision evaluation was found before to be less favourable compared to the other compounds.

*Cross-validation at the 500 ng/g level between ileal and caecal content*

| Compound | Level: 500 ng/g, caecum |
| --- | --- |
| Berberine | 486.7 ± 13.0 (acc -2.7%), RSD 2.7% |
| Demethyleneberberine | 468.9 ± 15.7 (acc -6.2 %), RSD 3.4% |
| Columbamine | 448.5 ± 12.6 (acc -10.3%), RSD 2.8% |
| Jatrorrhizine | 444.2 ± 15.3 (acc -11.2%), RSD 3.5% |
| Berberrubine | 470.4 ± 13.5 (acc -5.9%), RSD 2.9% |
| Dihydroberberine | 456.8 ± 11.5 (acc -8.6%), RSD 2.5% |
| Palmatine | 460.3 ± 14.0 (acc -7.9%), RSD 3.0% |
| Oxyberberine | 436.7 ± 21.2 (acc -12.7%), RSD 4.8% |

n = 6 replicates were prepared at the 500 ng/g level in caecal contents and quantified on a calibration curve issued from ileal contents. Mean values ± SD, as well as accuracy (% deviation from nominal value), and precision (as % RSD). Results concern quantification based on the internal standard tetrahydropalmatine.

Results are within the above given validation acceptance criteria for all components, suggesting the robustness of the analysis method, with a consistent response in between different intestinal segments, despite the quite different character in between ileum and caecum matrix. Found levels in caecum all have a negative accuracy towards the nominal level, suggested by the higher b values observed for calibration curves in ileum versus caecum. Nevertheless, accuracy remained well within the -20 % acceptance limit involved for the 500 ng/g level.

**INTESTINAL CONTENT SAMPLES – 1/2000 diluted sample extracts**

For quantification of analytes present at higher levels, the 1/2000 diluted samples were injected. Quantification was then based on the internal standard berberine-d6, which was added at a high level as well prior to sample extraction. Again, thalifendine was quantified on the berberrubine calibration curve and phase II metabolites were quantified relative to the berberine calibration curve, since no analytical reference standards were available for these compounds. Validation experiments of the dilution procedure were performed for berberine, demethyleneberberine, and berberrubine, being the main components present in intestinal contents at higher levels, and for which analytical standards were available.

*Evaluation of dilution integrity of demethyleneberberine, berberrubine, and berberine spiked at 100 µg/g in chicken intestinal contents.*

6 replicate samples were spiked with the 100 µg/g level, including the internal standard berberine-d6 at the 250 µg/mL level. Since this internal standard level was 400x the level included in the construction of the calibration curve (250 µg/mL vs. 0.625 µg/mL), and these sample extracts were diluted 1/400 compared to the dilution involved in the preparation of the calibration sample extracts (1/2000 vs. 1/5), the nominal concentration measured in reality for these samples is 250 ng/g, i.e. 100 µg/g/400, on the calibration curve established in the 62.5 - 5000 ng/g range.

| **Component** | **Matrix** | **Mean conc. (µg/g)** | **SD (µg/g)** | **RSD (%)** | **Accuracy (%)** |
| --- | --- | --- | --- | --- | --- |
| **Berberine** | Ileum | 84.9 (83.9) | 1.9 (1.2) | 2.3 (1.4) | -15.1 (-16.1) |
|  | Caecum | 84.1 (84.6) | 0.7 (0.2) | 0.8 (0.3) | -15.9 (-15.4) |
| **Demethyleneberberine** | Ileum | 110.0 (116.1) | 6.3 (27.1) | 5.7 (9.3) | + 10.0 (+16.1) |
|  | Caecum | 95.2 (103.7) | 1.2 (4.2) | 1.2 (4.0) | -4.8 (+3.7) |
| **Berberrubine** | Ileum | 95.6 (97.8) | 5.9 (3.3) | 6.2 (3.3) | - 4.4 (-2.2) |
|  | Caecum | 101.3 (105.2) | 1.6 (4.5) | 1.6 (4.3) | +1.3 (+5.2) |

Results from n = 6 replicates spiked at 100 µg/g were analyzed as described in the text. Results in brackets concern n = 3 processed sample extracts stored in the autosampler at 10 °C for 24h re-injected and quantified on a freshly prepared matrix-matched calibration curve.

Accuracy is within acceptance limits (-20 to + 10 %), as well as precision (< RSDmax = 10 %), despite the difference in composition of the samples and the calibration curve used for quantification, involving a dilution factor of 2000 and 5 respectively. Stability in extract was evaluated as well and no significant differences were observed as compared to initial measurement shortly after preparation.

Following results are comparable as the one presented before for 1/5 diluted intestinal content sample extract, and actually obtained on the same sample extracts, i.e. those of calibration curves in the 62.5 – 5000 ng/g range, as well as samples prepared at the 62.5, 500, and 5000 ng/g levels for evaluation of accuracy and precision, but now with the internal standard berberined-d6 being used for data processing and quantification, which was added at the low 0.625 µg/mL level. The data concern the components demethyleneberberine, berberrubine, and berberine only, since mainly the internal standard berberine-d6 was involved in quantification of these components at the high levels observed.

*Linearity of berberine, demethyleneberberine and berberrubine in chicken intestinal content (day 1, 2: ileum; day 3, 4: caecum)*

| **Compound** | **Evaluation occasion** | **a** | **b** | **c** | **R^2^** | **GoF (%)** |
| --- | --- | --- | --- | --- | --- | --- |
| **Berberine** | Day 1 | -3.031e-7 | 0.01649 | 0.01500 | 0.9989 | 2.6 |
|  | Day 2 | -3.817e-7 | 0.01608 | 0.02848 | 0.9978 | 3.7 |
|  | Day 3 | -1.800e-7 | 0.01645 | -0.01479 | 0.9991 | 2.3 |
|  | Day 4 | -2.882e-7 | 0.01587 | 0.01129 | 0.9997 | 1.4 |
| **Demethyleneberberine** | Day 1 | -4.051e-7 | 0.01072 | 0.01806 | 0.9991 | 2.4 |
|  | Day 2 | -4.758e-7 | 0.01044 | -0.008579 | 0.9991 | 2.3 |
|  | Day 3 | -4.035e-7 | 0.01065 | -0.05632 | 0.9987 | 2.9 |
|  | Day 4 | -4.746e-7 | 0.01018 | -0.01549 | 0.9990 | 2.6 |
| **Berberrubine** | Day 1 | -9.016e-7 | 0.03295 | -0.009391 | 0.9975 | 3.8 |
|  | Day 2 | -1.038e-6 | 0.03208 | -0.1703 | 0.9985 | 3.2 |
|  | Day 3 | -4.817e-7 | 0.02984 | -0.09881 | 0.9978 | 3.7 |
|  | Day 4 | -8.064e-7 | 0.02918 | -0.07233 | 0.9982 | 3.4 |

Matrix-matched calibration curves were prepared on two different analysis days for both ileum (day 1,2) and caecum (day 3,4). Results concern quantification based on the internal standard berberine-d6.

*Within-day and between-day accuracy and precision evaluation of berberine, demethyleneberberine and berberrubine in ileum (day 1, 2) and caecum (day 3, 4) contents at 62.5, 500, and 5000 ng/g levels*

| Compound | Time | Level 1: 62.5 ng/g | Level 2: 500 ng/g | Level 3: 5000 ng/g |
| --- | --- | --- | --- | --- |
| Berberine | **Day 1** | 61.8 ± 1.6 (acc -1.1%)  RSD 2.5% | 508.5 ± 5.3 (acc +1.7%)  RSD 1.0% | 5292.1 ± 355.4 (acc +5.8%)  RSD 6.7% |
|  | **Day 2** | 59.0 ± 1.2 (acc -5.7%)  RSD 2.0% | 507.1 ± 14.6 (acc +1.4%)  RSD 2.9% | 5144.5 ± 53.7 (acc +2.9%)  RSD 1.0% |
|  | **Day 3** | 64.0 ± 1.5 (acc +2.4%)  RSD 2.4% | 499.7 ± 9.4 (acc -0.1%)  RSD 1.9% | 4997.9 ± 84.0 (acc 0.0%)  RSD 1.7% |
|  | **Day 4** | 62.0 ± 1.8 (acc -0.8%)  RSD 2.9% | 510.5 ± 9.5 (acc +2..1%)  RSD 1.9% | 5449.0 ± 161.4 (acc +9.0%)  RSD 3.0% |
| Demethylene-berberine | **Day 1** | 59.4 ± 1.4 (acc -5.0%)  RSD 2.4% | 503.7 ± 16.9 (acc +0.7%)  RSD 3.4% | 5401.5 ± 386.3 (acc +8.0%)  RSD 7.2% |
|  | **Day 2** | 63.3 ± 0.9 (acc +1.2%)  RSD 1.3% | 508.4 ± 23.0 (acc +1.7%)  RSD 4.5% | 5140.0 ± 121.0 (acc +2.8%)  RSD 2.4% |
|  | **Day 3** | 65.7 ± 1.0 (acc +5.1%)  RSD 1.5% | 491.4 ± 9.0 (acc -1.7%)  RSD 1.8% | 4991.9 ± 93.9 (acc -0.2%)  RSD 1.9% |
|  | **Day 4** | 63.2 ± 2.4 (acc +1.1%)  RSD 3.8% | 504.2 ± 7.7 (acc +0.8%)  RSD 1.5% | 5602.6 ± 283.9 (acc +12.1%)^1^  RSD 5.1% |
| Berberrubine | **Day 1** | 60.5 ± 1.1 (acc -3.2%)  RSD 1.9% | 507.4 ± 12.6 (acc +1.5%)  RSD 2.5% | 5273.1 ± 396.1 (acc +5.5%)  RSD 7.5% |
|  | **Day 2** | 62.1 ± 2.0 (acc -0.6%)  RSD 3.3% | 506.5 ± 14.0 (acc +1.3%)  RSD 2.8% | 4999.1 ± 63.5 (acc -0.7%)  RSD 1.3% |
|  | **Day 3** | 66.4 ± 2.2 (acc +6.3%)  RSD 3.3% | 508.2 ± 12.3 (acc +1.6%)  RSD 2.4% | 5029.3 ± 102.0 (acc +0.6%)  RSD 2.0% |
|  | **Day 4** | 61.9 ± 1.2 (acc -1.0%)  RSD 2.0% | 512.4 ± 6.1 (acc +2.5%)  RSD 1.2% | 5475.1 ± 208.0 (acc +9.5%)  RSD 3.8% |

At each level evaluated, 62.5, 500, and 5000 ng/ng, n = 6 independent measurements were performed at each of 4 analysis days, in ileum for day 1 and day 2, and in caecum for day 3 and day 4. Mean values ± SD are given, as well as accuracy (% deviation from nominal value), and precision (as % RSD). Results concern quantification based on the internal standard berberine-d6. ^1^ borderline failing results, with accuracy > 10% acceptance criterion for accuracy.

*Stability of berberine, demethyleneberberine and berberrubine in processed intestinal content sample extracts at the 62.5, 500, and 5000 ng/g levels, stored at 10°C in autosampler for 24h*

| ILEUM | | | |
| --- | --- | --- | --- |
| Compound | **Level 1: 62.5 ng/g** | **Level 2: 500 ng/g** | **Level 3: 5000 ng/g** |
| Berberine | 60.5 ± 2.5 (acc -3.2%)  RSD 4.1% | 516.5 ± 13.5 (acc +3.3%)  RSD 2.6% | 5633.0 ± 274.4 (acc +12.7%)^1^  RSD 4.9% |
| Demethyleneberberine | 63.3 ± 2.9 (acc +1.3%)  RSD 4.5% | 530.8 ± 18.6 (acc +6.2%)  RSD 3.5% | 5822.2 ± 468.6 (acc +16.4%)^1^  RSD 8.0 |
| Berberrubine | 64.8 ± 1.7 (acc +3.7%)  RSD 2.7% | 519.8 ± 13.0 (acc +4.0%)  RSD 2.5% | 5534.8 ± 333.2 (acc +10.7%)^1^  RSD 6.0% |
| CAECUM | | | |
| Compound | **Level 1: 62.5 ng/g** | **Level 2: 500 ng/g** | **Level 3: 5000 ng/g** |
| Berberine | 63.6 ± 1.7 (acc +1.8%)  RSD 2.6% | 525.0 ± 18.5 (acc +5.0%)  RSD 3.5% | 5517.0 ± 47.7 (acc +10.3%)^1^  RSD 0.9% |
| Demethyleneberberine | 64.4 ± 2.0 (acc +3.1%)  RSD 3.1% | 520.0 ± 16.5 (acc +4.0%)  RSD 3.2% | 5760.1 ± 115.8 (acc +15.2%)^1^  RSD 2.0% |
| Berberrubine | 66.3 ± 3.2 (acc +6.0%)  RSD 4.8% | 521.3 ± 15.8 (acc +4.3%)  RSD 3.0% | 5546.0 ± 53.4 (acc +10.9%)^1^  RSD 1.0% |

At each level, n = 3 replicates were compared to a freshly prepared matrix-matched calibration curve. Results concern quantification based on the internal standard berberine-d6.^1^ accuracy for all components > 10% acceptance criterion.

*Cross-validation at the 500 ng/g level between ileal and caecal content*

| Compound | Level: 500 ng/g, caecum |
| --- | --- |
| Berberine | 538.2 ±8.0 (acc +7.6%), RSD 1.5% |
| Demethyleneberberine | 518.2 ± 11.6 (acc +3.6 %), RSD 2.2% |
| Berberrubine | 519.0 ± 7.4 (acc +3.8%), RSD 1.4% |

n = 6 replicates were prepared at the 500 ng/g level in caecal contents and quantified on a calibration curve issued from ileal contents. Mean values ± SD, as well as accuracy (% deviation from nominal value), and precision (as % RSD). Results concern quantification based on the internal standard berberine-d6.

The data suggest a good linear behavior, as well as a good accuracy and precision, if berberine-d6 is used as an internal standard as well. Also, stability in extract evaluation of processed sample extract during 24 h in the autosampler at 10 °C, as well a cross validation experiment caecum vs. ileum, showed comparable results whether tetrahydropalmatine or berberine-d6 were involved as an internal standard compound. Data for the other components (columbamine, jatrorrhizine, dihydroberberine, and oxyberberine) are not shown, but were favourable as well, with the exception of oxyberberine again. This indicates the flexibility of the analysis method to quantify on the same calibration curve and in the same sample extract both analytes at a low level and analytes at a high level, by including two internal standard compounds, one at a high level (berberine-d6) and one at a low level (tetrahydropalmatine) used respectively for a high (1/2000) and low (1/5) dilution of the sample extract.

# Supplementary Figures

**Supplementary Figure S1** – Growth of bacteria in presence of increasing concentrations of berberine in M2GSC medium (μg/mL) under anaerobic conditions. Strains were further tested for their ability to metabolize berberine (Figure 14). Strains were selected according to OTUs which relative abundance was positively correlated to berberrubine or thalifendine levels, including *Blautia spp.* and *A. butyraticus*. *E. limosum* was included as it was expected to demethylate berberine due to previous literature (Possemiers et al., 2008; Rich et al., 2022). Overnight cultures were 1:1000 diluted in M2GSC medium supplemented with 0, 1, 10 or 100 µg/mL berberine and grown for 24 hours under anaerobic conditions. Absorbance data at 600 nm are expressed as the mean ± standard deviation (n = 3). *: p < 0.05; ***: p < 0.001.


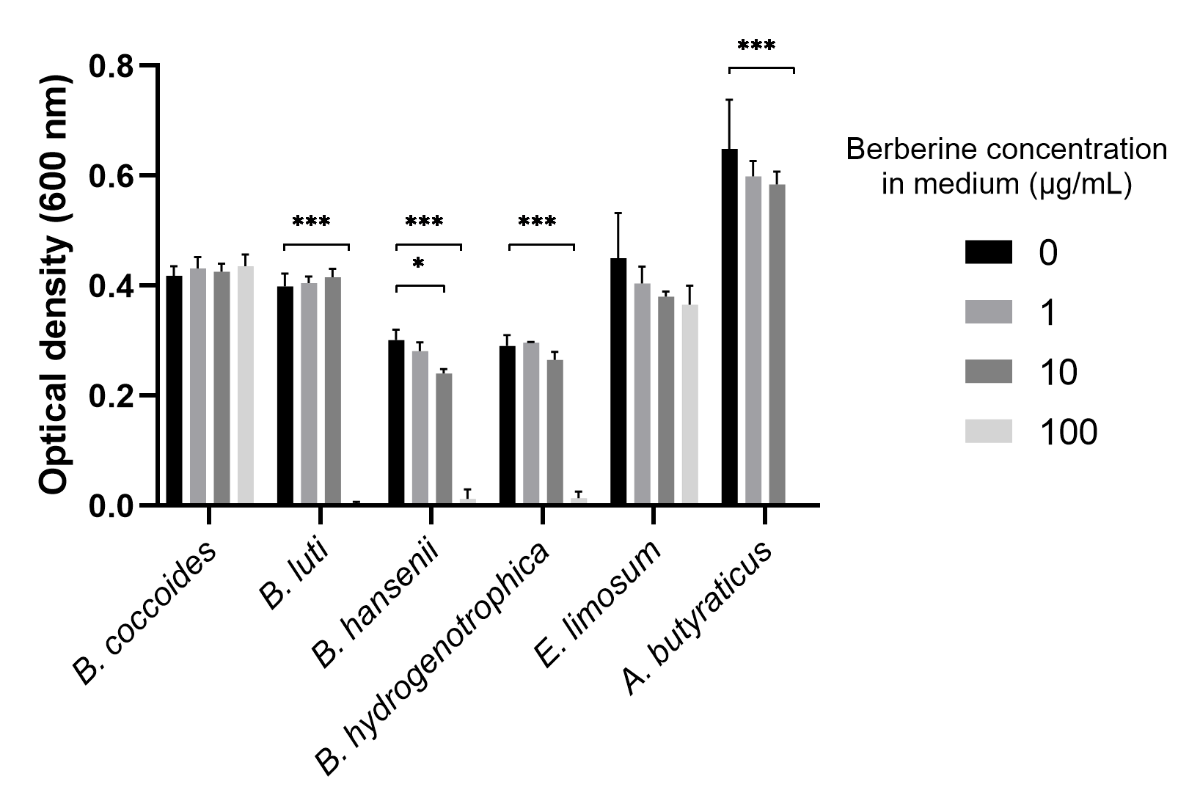


**Supplementary Figure S2** – Log_10_ concentration profiles of berberine, phase I and total phase II berberine-derived metabolites in the plasma from chickens fed a berberine-supplemented diet (0.1, 0.5 or 1 g/kg feed) for 21 days post-hatch. Total glucuronides and sulfates represent cumulated phase II berberine metabolites and include berberrubine-glucuronide/-sulfate, columbamine-glucuronide, jatrorrhizine-glucuronide/-sulfate, thalifendine-glucuronide, demethyleneberberine-glucuronide_01/_02/_03/_04/_05/_05/_06, demethyleneberberine-sulfate_03/_04/_plasma. See **Supplementary Figure S3** for details. Blank graphs represent undetected metabolites. Oxyberberine and columbamine were not present in plasma.


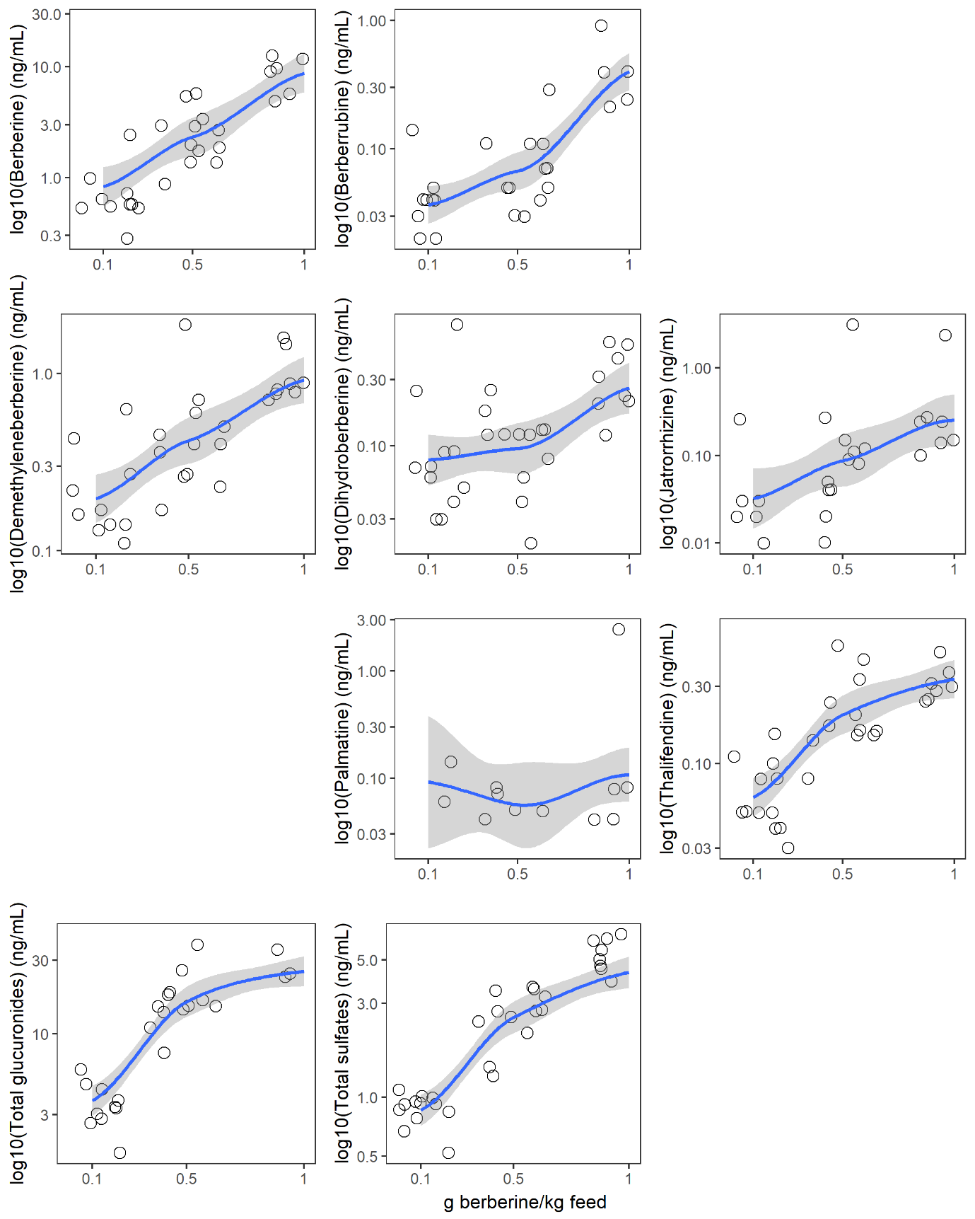


**Supplementary Figure S3** – Concentration profiles of phase II **(A)** glucuronide and **(B)** sulfate berberine metabolites in the plasma from chickens fed a berberine-supplemented diet (0.1, 0.5 or 1 g /kg feed) for 21 days post-hatch. Columbamine-sulfate, thalifendine-sulfate, demethyleneberberine-sulfate_01/_02/_05/_06 were not detected in the plasma, only in the intestine. Demethyleneberberine-sulfate_plasma was not detected in the intestine, only in the plasma.


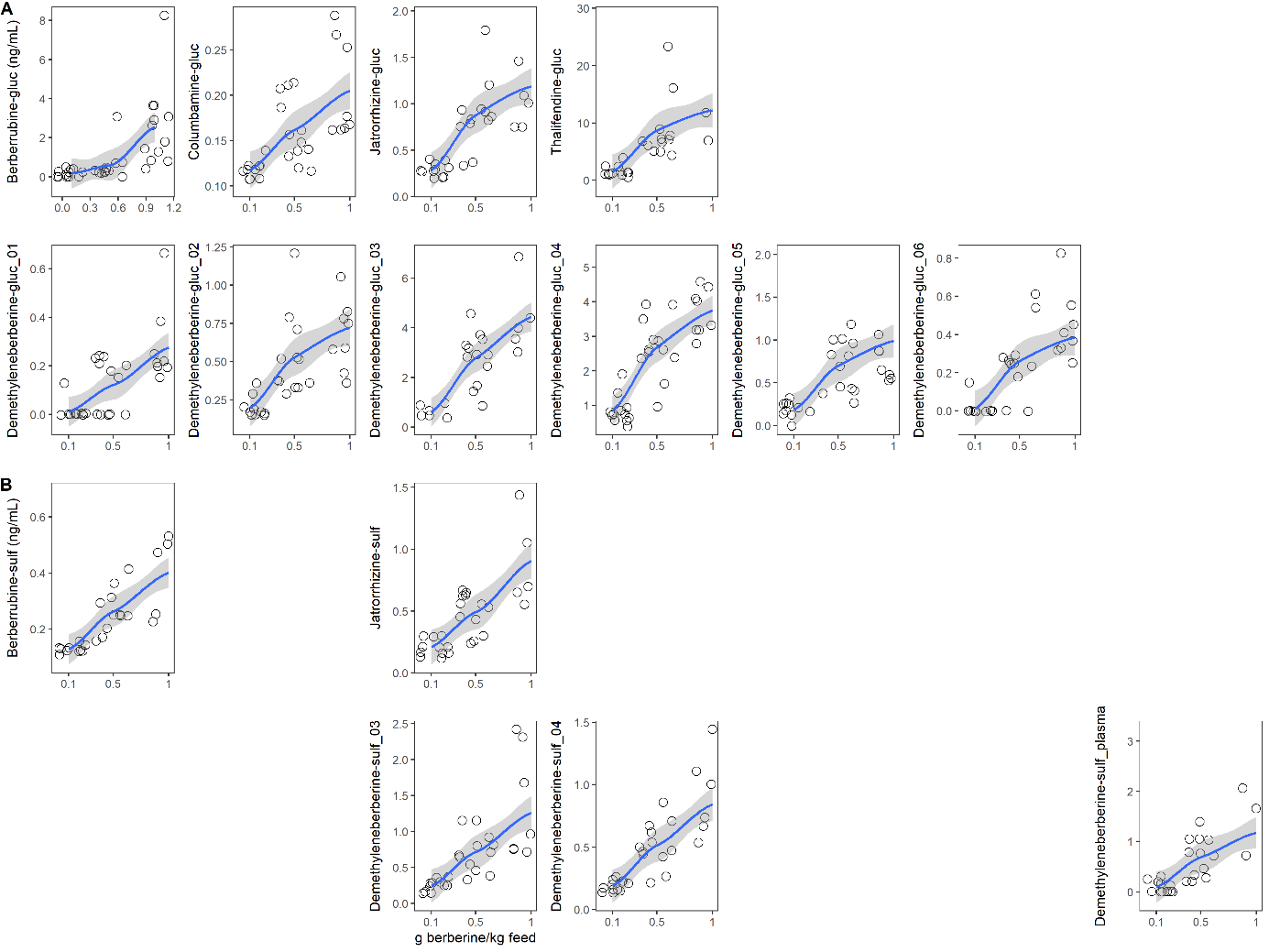


**Supplementary Figure S4** – Dihydroberberine stability in solution over 24 hours with (grey) or without (black) addition of vitamin C. A working solution of dihydroberberine at 10 ng/mL was prepared in Milli-Q water and supplemented or not with 17 mg/mL L-ascorbic acid (vitamin C) solution and injected during 24 hours of storage time in the autosampler at 10°C. Without stabilization with vitamin C, dihydroberberine converts back to berberine up to 66% within 24 hours.


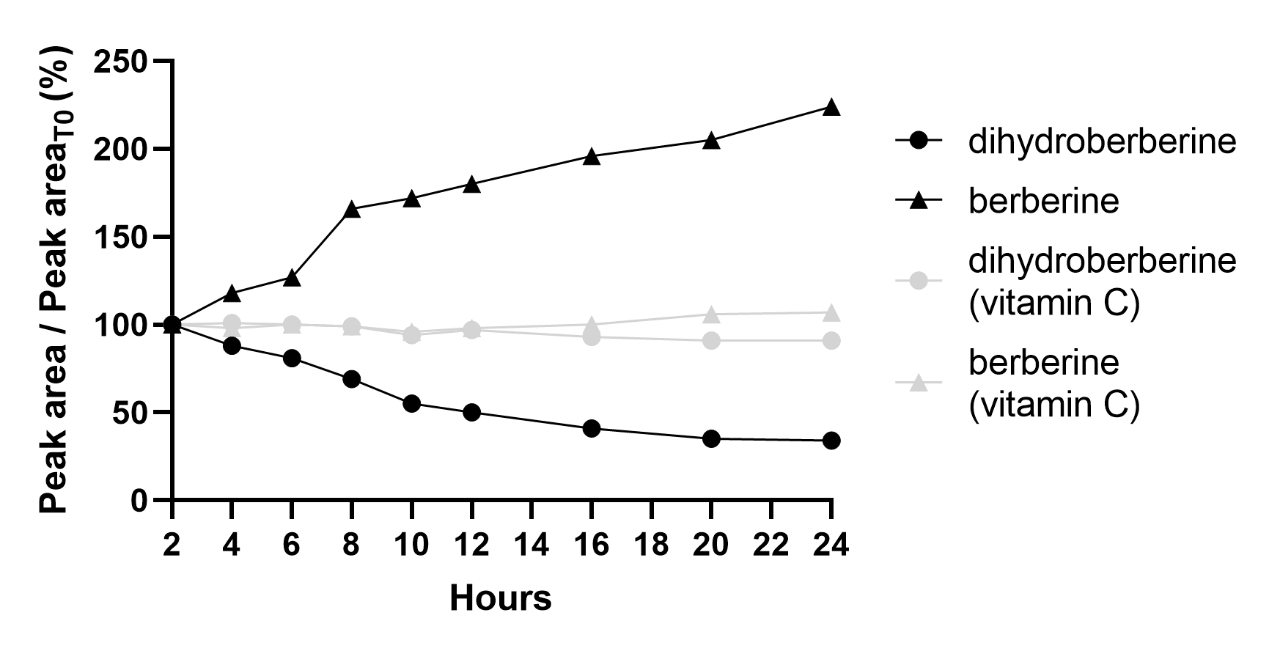


**Supplementary Figure S5** – Comparison of concentration profiles of phase II **(A)** glucuronide and **(B)** sulfate berberine metabolites between ileum (yellow) or caecum (purple) from chickens fed a berberine-supplemented diet (0.1, 0.5 or 1 g /kg feed) for 21 days post-hatch (n = 12). Supplementary Figure S2 reports concentration profiles in plasma.


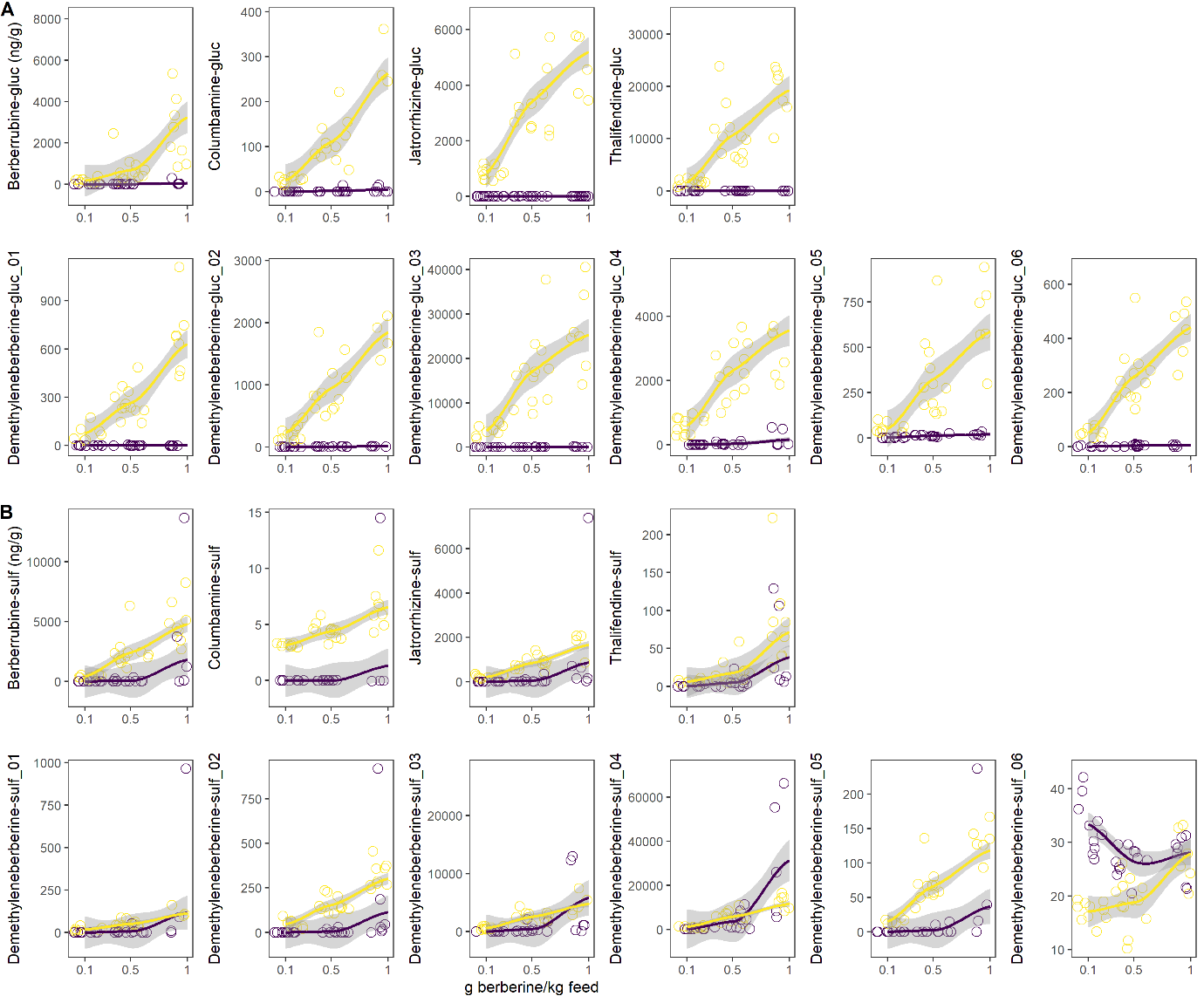


**Supplementary Figure S6** - Relative abundance of KEGG orthologs related to glucuronidase (K01195) or sulfatase (K01130, K01132, K01133, K01134, K01135, K01136, K01137, K01138) activity in the ileum or caecum of chickens fed either a normal diet (0) or a diet supplemented with 0.1, 0.5 or 1 g berberine/kg feed for 21 days post-hatch.

**
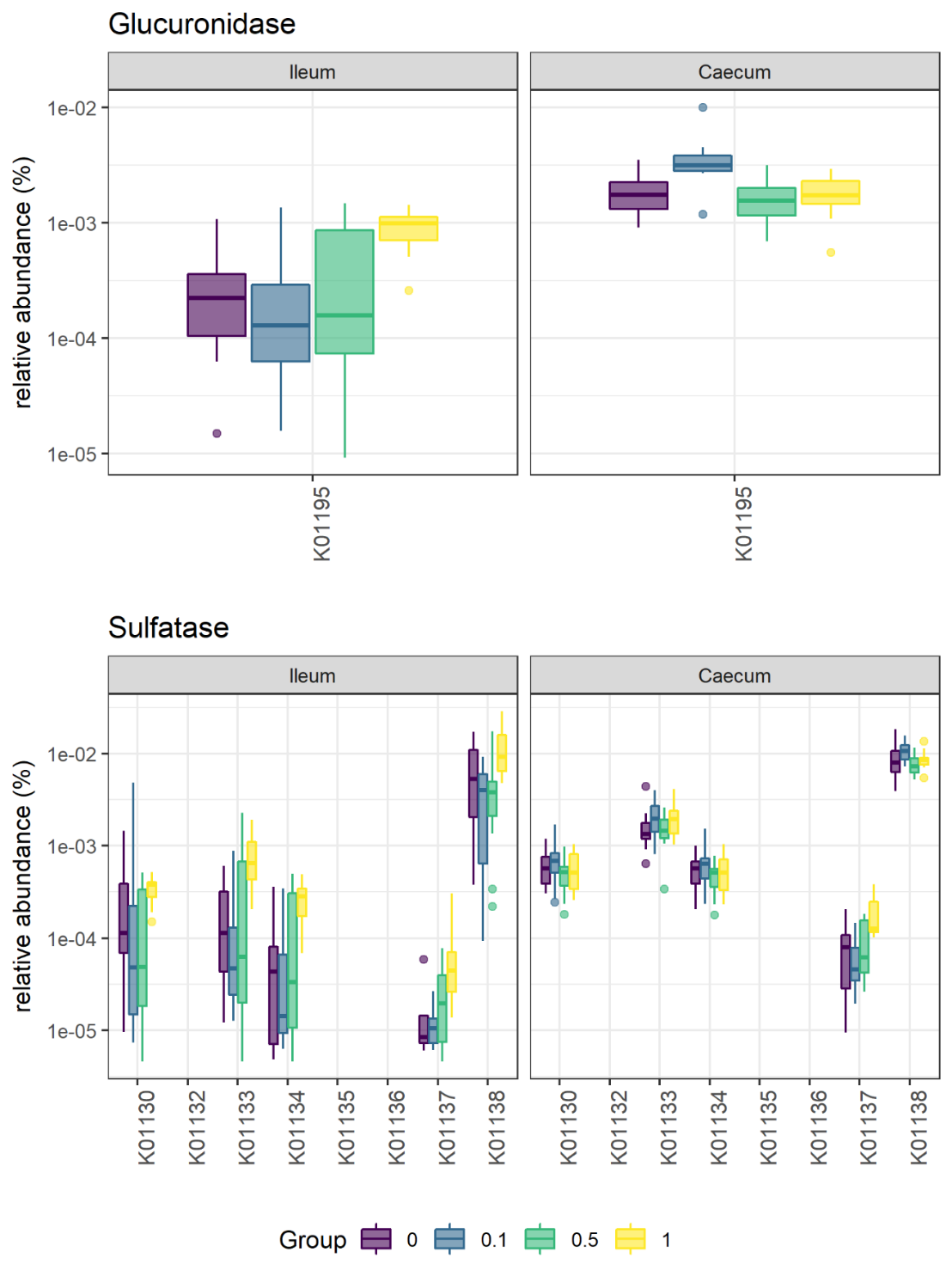
**

**Supplementary Figure S7** - Metabolite response **(A)** and metabolite production **(B)** in 10^-3^ diluted caecal content cultures after 48 hours incubation with 10 µg berberine/mL in anaerobic conditions. Jatrorrhizine and palmatine gave a similar response in the control medium when comparing with the microbiota culture, confirming that those two metabolites were impurities in the 98%-pure berberine powder and therefore do not result from the biotransformation of berberine by the gut microbiota. Metabolite production was expressed as log2 fold change, calculated as the ratio of the metabolite response in the microbiota culture at t = 48 h over the metabolite response in the control medium at t = 48 h. Data are expressed as the mean ± standard deviation (n = 3). An absolute fold change > 4 (|log2(fold change)| > 2) was considered as biologically relevant.

**
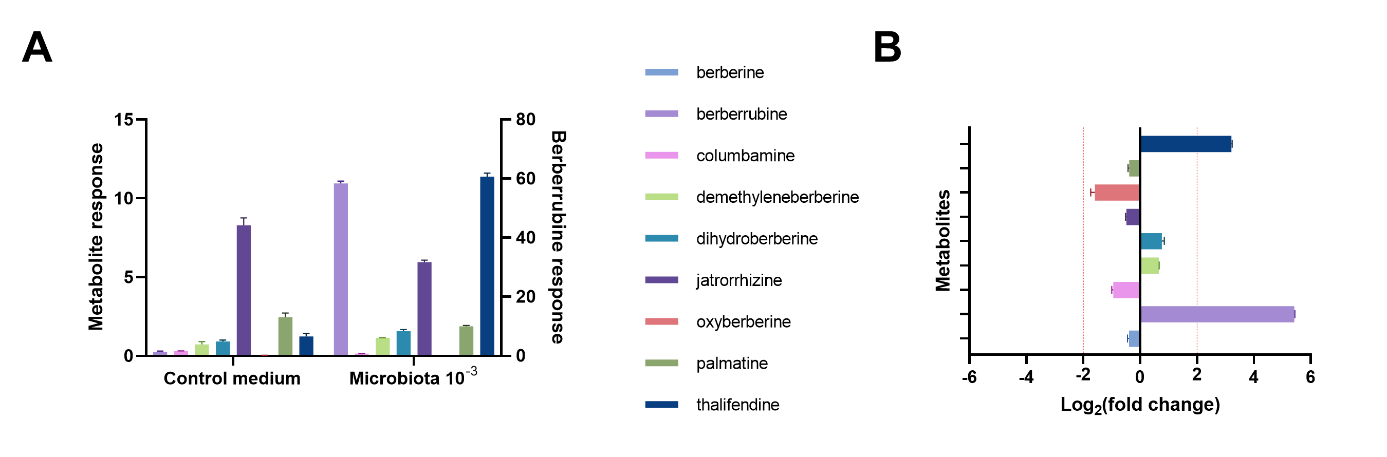
**

**Supplementary Figure S8** – Cytotoxicity assessment of berberine by Neutral Red Uptake assay in the human epithelial intestinal cell lines Caco-2 and T-84. **(A)** Cell viability. **(B)** IC50. Cells were exposed to a range of concentrations of berberine for 48 h. Neutral red medium was added, and after 3 h incubation at 37°C, the plate was washed and the dye was extracted with acidified ethanol solution. The plate was quantified at 540 nm (OD540 nm). The mean OD540 nm value from the wells without cells was subtracted from the rest of wells, and the values of treated cultures were referred to control non exposed cultures, to obtain the viability percentage of cells treated with berberine. IC50 was calculated using the Hill function analysis of the software GraphPad PRISM. Data are expressed as the mean ± standard deviation (n = 6).


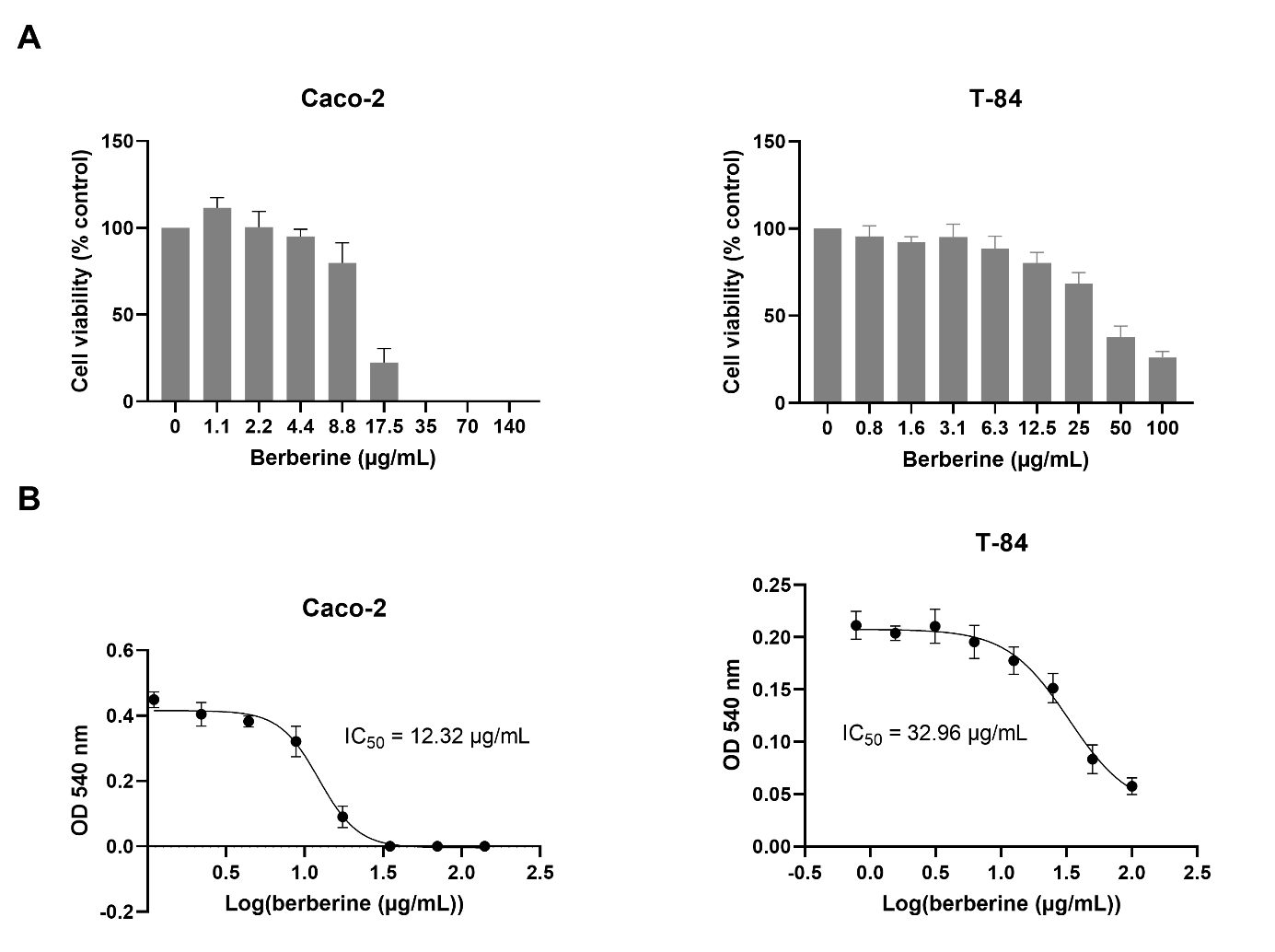


# Supplementary Tables

**Supplementary Table S1** – UPLC–MS/MS MRM (multiple reaction transitioning) transitions of berberine and its phase I and phase II metabolites in broiler chickens.

| Compound |  | Collision Energy (eV) | |
| --- | --- | --- | --- |
|  | MS/MS transitions (m/z) | Quantifier  Ion (first transition) | Qualifier  Ion (second transition) |
| berberine-d6 (internal standard) | 342.16 > 294.19 | 30 | - |
| tetrahydropalmatine (internal standard) | 356.20 > 191.99 | 25 | - |
| berberine | 336.14 > 320.16, 336.14 > 292.12 | 30 | 30 |
| berberrubine | 322.16 > 307.09, 322.16 > 279.11 | 25 | 25 |
| columbamine | 338.18 > 322.12, 338.18 > 279.10 | 30 | 40 |
| demethyleneberberine | 324.22 > 308.10, 324.22 > 280.06 | 30 | 40 |
| dihydroberberine | 338.18 > 322.12, 338.19 > 304.15 | 25 | 35 |
| jatrorrhizine | 338.18 > 322.12, 338.18 > 279.10 | 25 | 35 |
| oxyberberine | 352.19 > 337.12, 352.19 > 322.12 | 30 | 30 |
| palmatine | 352.19 > 336.16, 352.19 > 308.12 | 30 | 30 |
| thalifendine | 322.16 > 307.09, 322.16 > 279.11 | 25 | 20 |
| thalifendine-glucuronide | 498.20 > 322.20 | 25 | - |
| berberrubine-glucuronide | 498.20 > 322.20 | 25 | - |
| columbamine-glucuronide | 514.20 > 338.20 | 25 | - |
| jatrorrhizine-glucuronide | 514.20 > 338.20 | 25 | - |
| demethyleneberberine-glucuronide_01/_02/_03/_04/_05/_06 | 500.20 > 324.20 | 25 | - |
| columbamine-sulfate | 418.20 > 338.20 | 20 | - |
| jatrorrhizine-sulfate | 418.20 > 338.20 | 20 | - |
| thalifendine-sulfate | 402.20 > 322.20 | 20 | - |
| berberrubine-sulfate | 402.20 > 322.20 | 20 | - |
| demethyleneberberine-sulfate_plasma | 404.20 > 324.20 | 20 | - |
| demethyleneberberine-sulfate_01/_02/_03/_04/_05/_06* | 404.20 > 324.20 | 20 | - |

*since both demethyleneberberine and demethylated jatrorrhizine and columbamine have the same molecular mass (m/z at 324) and each compound has 2 OH-groups available for glucuronidation or sulfation, in total 6 possible glucuronide or 6 sulfate metabolites were included in the UPLC-MS/MS analysis. For practical reasons, the name was mentioned as demethyleneberberine-glucuronide_01/_02/_03/_04/_05/_06 or demethyleneberberine-sulfate_01/_02/_03/_04/_05/_06.

**Supplementary Table S2** – Effect of the berberine supplementation in-feed on the community structure in the jejunum, ileum, caecum or colon of 21-day old chickens. Beta diversity was assessed using Bray–Curtis or unweighted UniFrac dissimilarities. Differences in the community structure were tested using the commonly used PERMANOVA test. Post hoc analysis results on the significant berberine supplementation effects are listed in Table 2.

|  | Bray-Curtis | | Unweighted UniFrac | |
| --- | --- | --- | --- | --- |
|  | **R^2^ (%)** | **p-value** | **R^2^ (%)** | **p-value** |
| Jejunum | 9.1 | 0.119 | 10.7 | **0.006** |
| Ileum | 24.9 | **0.001** | 16.3 | **0.001** |
| Caecum | 17.0 | **0.001** | 19.7 | **0.001** |
| Colon | 18.5 | **0.001** | 15.4 | **0.001** |

R^2^ represents the percentage of variation accountable for the supplementation of berberine in the feed.

**Supplementary Table S3** – Statistical results of the post hoc analysis on the berberine supplementation dose-effect (0.1, 0.5 or 1 g/kg feed) on the microbial community structure in the jejunum, ileum, caecum or colon after 21 days. Beta diversity was assessed using the Bray–Curtis or unweighted UniFrac dissimilarities (n = 12). Differences in the community structure were tested using PERMANOVA (Supplementary Table S2). There was no significant difference between groups in the community structure in the jejunum according to Bray-Curtis. Post hoc analysis was performed using the pairwise PERMANOVA, resulting in the Bonferroni corrected adjusted p-values.

|  | Bray-Curtis | Unweighted UniFrac |
| --- | --- | --- |
|  | **PERMANOVA adjusted p-values** | |
| Jejunum | | |
| 0 vs 0.1 | - | **0.063** |
| 0 vs 0.5 | - | 0.214 |
| 0 vs 1 | **-** | 1.000 |
| Ileum | | |
| 0 vs 0.1 | 0.280 | **0.040** |
| 0 vs 0.5 | 1.000 | 0.230 |
| 0 vs 1 | **0.001** | **0.002** |
| Caecum | | |
| 0 vs 0.1 | **0.004** | **0.006** |
| 0 vs 0.5 | **0.001** | **0.007** |
| 0 vs 1 | **0.001** | **0.007** |
| Colon | | |
| 0 vs 0.1 | 0.239 | **0.035** |
| 0 vs 0.5 | 1.000 | 0.416 |
| 0 vs 1 | **0.002** | 0.081 |

**Supplementary Table S4** – Differentially abundant families in the jejunum, ileum, caecum or colon between chickens fed a diet supplemented with 0.1, 0.5 or 1 g berberine/kg feed and chickens fed a normal diet for 21 days post-hatch (n = 12).

| Phylum | Class | Order | Family | Mean relative abundance (%) | | | | 0.1 vs 0 | | 0.5 vs 0 | | 1 vs 0 | |
| --- | --- | --- | --- | --- | --- | --- | --- | --- | --- | --- | --- | --- | --- |
|  |  |  |  | **0** | **0.1** | **0.5** | **1** | **L2FC** | **p_adj_** | **L2FC** | **p_adj_** | **L2FC** | **p_adj_** |
| Jejunum | | | | | | | | | | | | | |
| *Firmicutes* | *Bacilli* | *Bacillales* | *Bacillaceae* | 0.01 | 0.03 | 0.04 | 0.04 | 3.45 | **0.000** | 1.43 | 0.434 | 0.80 | 0.607 |
| *Proteobacteria* | *Gammaproteobacteria* | *Betaproteobacteriales* | *Burkholderiaceae* | 0.93 | 6.47 | 5.60 | 3.55 | 3.82 | **0.000** | 0.67 | 0.681 | 1.37 | 0.522 |
| *Firmicutes* | *Clostridia* | *Clostridiales* | *Peptostreptococcaceae* | 1.94 | 6.37 | 0.52 | 0.26 | 3.90 | **0.017** | -1.12 | 0.614 | -3.21 | 0.280 |
| *Proteobacteria* | *Gammaproteobacteria* | *Pseudomonadales* | *Pseudomonadaceae* | 0.12 | 1.71 | 0.35 | 0.35 | 3.67 | **0.000** | 0.14 | 0.857 | 1.01 | 0.527 |
| *Firmicutes* | *Clostridia* | *Clostridiales* | *Ruminococcaceae* | 4.33 | 3.73 | 4.52 | 10.54 | 2.19 | **0.017** | -0.14 | 0.857 | 1.51 | 0.492 |
| *Tenericutes* | *Mollicutes* | *Mollicutes RF39* | *uncultured bacterium* | 0.03 | 0.01 | 0.02 | 0.00 | 2.35 | **0.017** | 0.60 | 0.686 | 0.00 | 0.999 |
| Ileum | | | | | | | | | | | | | |
| *Firmicutes* | *Clostridia* | *Clostridiales* | *Lachnospiraceae* | 4.36 | 2.54 | 14.77 | 26.87 | -0.49 | 0.922 | -0.17 | 0.954 | 2.99 | **0.009** |
| *Firmicutes* | *Clostridia* | *Clostridiales* | *Peptostreptococcaceae* | 29.27 | 39.1 | 24.91 | 0.73 | 1.55 | 0.903 | -0.50 | 0.905 | -6.91 | **0.004** |
| *Firmicutes* | *Clostridia* | *Clostridiales* | *Ruminococcaceae* | 0.85 | 1.35 | 6.11 | 8.08 | 0.41 | 0.922 | 0.24 | 0.954 | 3.41 | **0.009** |
| Caecum | | | | | | | | | | | | | |
| *Firmicutes* | *Clostridia* | *Clostridiales* | *Peptostreptococcaceae* | 0.76 | 0.66 | 0.56 | 0.00 | 0.02 | 0.988 | -0.55 | 0.807 | -7.85 | **0.001** |
| Colon | | | | | | | | | | | | | |
| *Firmicutes* | *Clostridia* | *Clostridiales* | *Peptostreptococcaceae* | 28.05 | 14.07 | 15.73 | 0.51 | -0.79 | 0.869 | -0.94 | 0.929 | -8.67 | **0.000** |

The taxonomic classification and the log_2_ fold change (L2FC) of the LinDA-normalized abundance of each family are shown.

**Supplementary Table S5** – Differentially abundant genera in the jejunum, ileum, caecum or colon between chickens fed a diet supplemented with 0.1, 0.5 or 1 g berberine/kg feed and chickens fed a normal diet for 21 days post-hatch (n = 12).

| Phylum | Class | Order | Family | Genus | Mean relative abundance (%) | | | | 0.1 vs 0 | | 0.5 vs 0 | | 1 vs 0 | |
| --- | --- | --- | --- | --- | --- | --- | --- | --- | --- | --- | --- | --- | --- | --- |
|  |  |  |  |  | **0** | **0.1** | **0.5** | **1** | **L2**  **FC** | **p_adj_** | **L2**  **FC** | **p_adj_** | **L2**  **FC** | **p_adj_** |
| Jejunum | | | | | | | | | | | | | | |
| *Actinobacteria* | *Coriobacteriia* | *Coriobacteriales* | *Eggerthellaceae* | *Gordonibacter* | 0.05 | 0.01 | 0.00 | 0.02 | -2.71 | **0.050** | -2.33 | 0.351 | -1.79 | 0.197 |
| *Actinobacteria* | *Actinobacteria* | *Micrococcales* | *Microbacteriaceae* | *Curtobacterium* | 0.11 | 0.01 | 0.03 | 0.06 | -2.73 | **0.045** | -1.13 | 0.658 | -2.09 | 0.197 |
| *Firmicutes* | *Clostridia* | *Clostridiales* | *Ruminococcaceae* | *Butyricicoccus* | 0.77 | 0.22 | 0.34 | 0.84 | -2.02 | **0.045** | -0.78 | 0.664 | -0.38 | 0.931 |
| Ileum | | | | | | | | | | | | | | |
| *Proteobacteria* | *Gamma-proteobacteria* | *Beta-proteobacteriales* | *Burkholderiaceae* | *Ralstonia* | 0.10 | 0.04 | 0.05 | 0.37 | -0.96 | 0.989 | -1.95 | 0.922 | -5.90 | **0.027** |
| *Proteobacteria* | *Gamma-proteobacteria* | *Enterobacteriales* | *Enterobacteriaceae* | *Cronobacter* | 0.00 | 0.00 | 0.00 | 0.00 | 0.38 | 0.989 | -0.95 | 0.712 | -3.64 | **0.000** |
| *Proteobacteria* | *Gamma-proteobacteria* | *Enterobacteriales* | *Enterobacteriaceae* | *Serratia* | 0.00 | 0.00 | 0.00 | 0.00 | 0.03 | 0.989 | -0.80 | 0.917 | -3.49 | **0.000** |
| *Firmicutes* | *Clostridia* | *Clostridiales* | *Lachnospiraceae* | *Lachnoclostridium 5* | 0.00 | 0.00 | 0.02 | 0.00 | 0.40 | 0.989 | -0.09 | 0.962 | -3.40 | **0.000** |
| *Firmicutes* | *Clostridia* | *Clostridiales* | *Lachnospiraceae* | *Tyzzerella* | 0.00 | 0.00 | 0.00 | 0.01 | 0.06 | 0.989 | -0.72 | 0.922 | -2.59 | **0.001** |
| *Actinobacteria* | *Actinobacteria* | *Micrococcales* | *Microbacteriaceae* | *Curtobacterium* | 0.04 | 0.01 | 0.02 | 0.03 | -0.49 | 0.989 | -1.50 | 0.712 | -2.66 | **0.027** |
| *Actinobacteria* | *Actinobacteria* | *Micrococcales* | *Micrococcaceae* | *Glutamicibacter* | 0.01 | 0.01 | 0.01 | 0.01 | 0.33 | 0.989 | -0.49 | 0.922 | -2.76 | **0.005** |
| *Firmicutes* | *Clostridia* | *Clostridiales* | *Peptostreptococcaceae* | *Family_*  *Peptostreptococcaceae* | 0.20 | 0.37 | 0.15 | 0.01 | 2.07 | 0.832 | -0.52 | 0.922 | -7.51 | **0.001** |
| *Firmicutes* | *Clostridia* | *Clostridiales* | *Peptostreptococcaceae* | *uncultured* | 28.96 | 38.50 | 24.63 | 0.72 | 1.99 | 0.832 | -0.24 | 0.962 | -9.51 | **0.001** |
| *Firmicutes* | *Clostridia* | *Clostridiales* | *Peptostreptococcaceae* | *Romboutsia* | 0.11 | 0.24 | 0.13 | 0.01 | 2.23 | 0.832 | -0.07 | 0.986 | -6.95 | **0.001** |
| *Firmicutes* | *Clostridia* | *Clostridiales* | *Ruminococcaceae* | *Faecalibacterium* | 0.00 | 0.02 | 0.02 | 0.01 | 1.89 | 0.452 | 1.01 | 0.896 | -2.86 | **0.001** |
| *Tenericutes* | *Mollicutes* | *Mollicutes RF39* | *uncultured* | *uncultured* | 0.00 | 0.00 | 0.02 | 0.00 | 0.63 | 0.989 | 0.65 | 0.922 | -3.09 | **0.000** |
| Caecum | | | | | | | | | | | | | | |
| *Firmicutes* | *Clostridia* | *Clostridiales* | *Lachnospiraceae* | *Fusicatenibacter* | 0.04 | 0.06 | 0.32 | 0.12 | 1.41 | 0.439 | 3.47 | **0.020** | 2.41 | 0.212 |
| *Firmicutes* | *Clostridia* | *Clostridiales* | *Lachnospiraceae* | *Family_*  *Lachnospiraceae* | 2.67 | 2.08 | 5.61 | 5.61 | -0.10 | 0.875 | 1.25 | **0.007** | 0.72 | 0.212 |
| *Firmicutes* | *Clostridia* | *Clostridiales* | *Lachnospiraceae* | *ASF356* | 0.04 | 0.40 | 0.38 | 0.32 | 3.88 | 0.087 | 4.47 | **0.039** | 3.78 | 0.198 |
| *Firmicutes* | *Clostridia* | *Clostridiales* | *Lachnospiraceae* | *CHKCI001* | 0.01 | 0.02 | 0.03 | 0.02 | 1.33 | 0.087 | 2.34 | **0.000** | 1.13 | 0.212 |
| *Firmicutes* | *Clostridia* | *Clostridiales* | *Peptostreptococcaceae* | *uncultured* | 0.73 | 0.63 | 0.54 | 0.00 | 0.26 | 0.875 | -0.52 | 0.941 | -8.12 | **0.002** |
| Colon | | | | | | | | | | | | | | |
| *Firmicutes* | *Clostridia* | *Clostridiales* | *Lachnospiraceae* | *GCA-900066575* | 0.14 | 0.59 | 0.53 | 0.65 | 3.13 | **0.039** | 1.10 | 0.615 | 2.34 | 0.168 |
| *Firmicutes* | *Clostridia* | *Clostridiales* | *Lachnospiraceae* | *Lachnospiraceae UCG-010* | 0.01 | 0.06 | 0.03 | 0.06 | 2.34 | **0.029** | 0.64 | 0.730 | 0.99 | 0.558 |
| *Firmicutes* | *Clostridia* | *Clostridiales* | *Peptostreptococcaceae* | *Romboutsia* | 0.16 | 0.14 | 0.11 | 0.00 | -0.31 | 0.891 | -1.42 | 0.572 | -6.45 | **0.000** |
| *Firmicutes* | *Clostridia* | *Clostridiales* | *Peptostreptococcaceae* | *Family_*  *Peptostreptococcaceae* | 0.19 | 0.11 | 0.13 | 0.01 | -1.21 | 0.598 | -2.31 | 0.572 | -6.62 | **0.010** |
| *Firmicutes* | *Clostridia* | *Clostridiales* | *Peptostreptococcaceae* | *uncultured* | 27.70 | 13.82 | 15.49 | 0.50 | -1.31 | 0.576 | -1.92 | 0.595 | -9.04 | **0.001** |
| *Firmicutes* | *Clostridia* | *Clostridiales* | *Ruminococcaceae* | *Ruminococcaceae UCG-014* | 0.06 | 3.36 | 1.19 | 1.02 | 5.39 | **0.001** | 2.59 | 0.414 | 3.32 | 0.072 |

The taxonomic classification and the log_2_ fold change (L2FC) of the LinDA-normalized abundance of each genus are shown.

**Supplementary Table S6** – Effect of the compartment on berberine intestinal metabolism (linear model).

|  | 0 | 0.1 | 0.5 | 1 |
| --- | --- | --- | --- | --- |
| **Berberine** |  |  |  |  |
| Ratio caecum / ileum | 0.31 | 0.07 | 0.14 | 0.22 |
| P-value | **< 0.0001** | **< 0.0001** | **< 0.0001** | **< 0.0001** |
| **Berberrubine** |  |  |  |  |
| Ratio caecum / ileum | 2.41 | 21.43 | 29.98 | 63.80 |
| P-value | **0.0163** | **< 0.0001** | **< 0.0001** | **< 0.0001** |
| **Columbamine** |  |  |  |  |
| Ratio caecum / ileum | 1 | 0.27 | 0.32 | 0.44 |
| P-value | 1 | **< 0.0001** | **< 0.0001** | **< 0.0001** |
| **Demethyleneberberine** |  |  |  |  |
| Ratio caecum / ileum | 3.62 | 15.66 | 30.48 | 32.10 |
| P-value | **< 0.0001** | **< 0.0001** | **< 0.0001** | **< 0.0001** |
| **Dihydroberberine** |  |  |  |  |
| Ratio caecum / ileum | 1 | 0.75 | 0.27 | 0.44 |
| P-value | 1 | 0.0631 | **< 0.0001** | **< 0.0001** |
| **Jatrorrhizine** |  |  |  |  |
| Ratio caecum / ileum | 1.26 | 0.31 | 0.49 | 0.72 |
| P-value | 0.3371 | **< 0.0001** | **0.0050** | 0.1891 |
| **Oxyberberine** |  |  |  |  |
| Ratio caecum / ileum | 5.04 | 1.94 | 1.48 | 0.95 |
| P-value | **< 0.0001** | **0.0282** | 0.1995 | 0.8697 |
| **Palmatine** |  |  |  |  |
| Ratio caecum / ileum | 0.29 | 0.13 | 0.11 | 0.26 |
| P-value | **< 0.0001** | **< 0.0001** | **< 0.0001** | **< 0.0001** |
| **Thalifendine** |  |  |  |  |
| Ratio caecum / ileum | 1.21 | 6.68 | 29.03 | 21.17 |
| P-value | 0.3462 | **< 0.0001** | **< 0.0001** | **< 0.0001** |
| **Total glucuronides** |  |  |  |  |
| Ratio caecum / ileum | 1 | 0.002 | 0.001 | 0.003 |
| P-value | 1 | **< 0.0001** | **< 0.0001** | **< 0.0001** |
| **Total sulfates** |  |  |  |  |
| Ratio caecum / ileum | 1 | 0.06 | 0.10 | 1.01 |
| P-value | 1 | **< 0.0001** | **< 0.0001** | 0.9868 |
| **Deme_sulf_04** |  |  |  |  |
| Ratio caecum / ileum | 1 | 0.07 | 0.34 | 1.7625 |
| P-value | 1 | **< 0.0001** | **0.0020** | 0.1000 |
| **Deme_sulf_06** |  |  |  |  |
| Ratio caecum / ileum | 1 | 1.89 | 1.42 | 1.02 |
| P-value | 1 | **< 0.0001** | **< 0.0001** | 0.6996 |

**Supplementary Table S7** – Differentially abundant OTUs between the BBR and the Control group in the total (DNA) or active (cDNA) bacterial community across the eight dilutions of caecal inoculum, after 48 h-anaerobic growth. Only the OTUs satisfying an absolute log_2_ fold change > 1.5 are displayed.

|  |  |  |  | BBRRNA vs ControlRNA | BBRDNA vs ControlDNA | BBRRNA vs BBRDNA | ControlRNA vs ControlDNA |  |
| --- | --- | --- | --- | --- | --- | --- | --- | --- |
| **Phylum** | **Family** | **Genus** | **OTU** | **L2FC** | | | | **p_adj_** |
| *Proteo-bacteria* | *Burkholderiaceae* | *Ralstonia* | New.ReferenceOTU230 | 1.34 | 0.44 | **3.40** | **2.49** | < 0.001 |
| *Proteo-bacteria* | *Enterobacteriaceae* | *Escherichia-Shigella* | New.ReferenceOTU476 | -0.42 | -0.75 | **1.67** | 1.34 | < 0.001 |
| *Firmicutes* | *Erysipelotrichaceae* | *Erysipelatoclostridium* | New.ReferenceOTU462 | -0.55 | -0.78 | **1.51** | 1.27 | 0.001 |
| *Firmicutes* | *Erysipelotrichaceae* | *Erysipelatoclostridium* | New.ReferenceOTU144 | 0.42 | 0.55 | **1.64** | **1.77** | < 0.001 |
| *Firmicutes* | *Erysipelotrichaceae* | *Erysipelatoclostridium* | New.ReferenceOTU429 | 0.23 | 0.65 | **1.73** | **2.14** | < 0.001 |
| *Firmicutes* | *Erysipelotrichaceae* | *Erysipelatoclostridium* | 592616 | 0.37 | 0.67 | **1.49** | **1.79** | 0.043 |
| *Firmicutes* | *Erysipelotrichaceae* | *Erysipelatoclostridium* | New.ReferenceOTU118 | **-1.90** | -0.82 | -0.75 | 0.33 | 0.012 |
| *Firmicutes* | *Erysipelotrichaceae* | *Erysipelatoclostridium* | 233953 | **-1.85** | -1.14 | -0.21 | 0.50 | 0.019 |
| *Firmicutes* | *Erysipelotrichaceae* | *Erysipelatoclostridium* | New.CleanUp.ReferenceOTU4902 | **-2.19** | -1.03 | -0.05 | 1.12 | 0.009 |
| *Firmicutes* | *Eubacteriaceae* | *Eubacterium* | 109633 | **1.92** | **1.75** | -0.64 | -0.81 | 0.015 |
| *Firmicutes* | *Lachnospiraceae* | *Family_Lachnospiraceae* | 843454 | 0.55 | -0.19 | -1.17 | **-1.91** | 0.017 |
| *Firmicutes* | *Lachnospiraceae* | *uncultured* | 293975 | **-2.01** | **-1.62** | 0.18 | 0.57 | < 0.001 |
| *Firmicutes* | *Lachnospiraceae* | *[Eubacterium] xylanophilum group* | 356073 | **-1.98** | **-1.79** | -1.05 | -0.86 | 0.008 |
| *Firmicutes* | *Lachnospiraceae* | *Anaerostipes* | 1107057 | -0.90 | -1.14 | **-2.55** | **-2.79** | < 0.001 |
| *Firmicutes* | *Lachnospiraceae* | *uncultured* | 180099 | -0.96 | **-1.55** | -0.59 | -1.18 | < 0.001 |
| *Firmicutes* | *Lachnospiraceae* | *uncultured* | 2265722 | **2.76** | 1.22 | -0.46 | **-1.99** | 0.026 |
| *Firmicutes* | *Lachnospiraceae* | *uncultured* | 368615 | -0.96 | -0.96 | **-1.57** | **-1.57** | < 0.001 |
| *Firmicutes* | *Lachnospiraceae* | *Lachnospiraceae UCG-010* | New.ReferenceOTU111 | -1.27 | -0.42 | **-1.92** | -1.07 | < 0.001 |
| *Firmicutes* | *Lachnospiraceae* | *uncultured* | 574038 | **-1.65** | **-2.16** | -0.24 | -0.74 | < 0.001 |
| *Firmicutes* | *Lachnospiraceae* | *[Ruminococcus] torques group* | 563086 | -0.24 | -0.39 | **-1.93** | **-2.08** | < 0.001 |
| *Firmicutes* | *Lachnospiraceae* | *[Ruminococcus] torques group* | 249142 | **-3.17** | **-2.54** | -0.87 | -0.25 | < 0.001 |
| *Firmicutes* | *Lachnospiraceae* | *[Ruminococcus] torques group* | 199555 | -1.39 | **-1.72** | -0.15 | -0.48 | 0.008 |
| *Firmicutes* | *Lactobacillaceae* | *Lactobacillus* | 813944 | **1.85** | **1.53** | 1.33 | 1.02 | < 0.001 |
| *Bacteroi-detes* | *Rikenellaceae* | *Alistipes* | 157573 | -0.29 | 0.19 | **1.91** | **2.39** | < 0.001 |
| *Firmicutes* | *Ruminococcaceae* | *Anaerofilum* | 186881 | -1.11 | **-1.53** | -0.59 | -1.01 | < 0.001 |
| *Firmicutes* | *Ruminococcaceae* | *Anaerofilum* | New.ReferenceOTU85 | -1.45 | **-1.62** | -0.84 | -1.01 | < 0.001 |
| *Firmicutes* | *Ruminococcaceae* | *uncultured* | 4404181 | 0.08 | 0.40 | **-1.72** | -1.40 | < 0.001 |
| *Firmicutes* | *Ruminococcaceae* | *Ruminococcaceae UCG-013* | 234912 | **-2.52** | **-3.19** | -0.46 | -1.13 | < 0.001 |
| *Firmicutes* | *Ruminococcaceae* | *Ruminococcaceae UCG-014* | 461487 | -0.55 | -0.15 | **-3.30** | **-2.90** | 0.012 |
| *Firmicutes* | *Ruminococcaceae* | *Family_Ruminococcaceae* | 129401 | 1.00 | 0.84 | **1.99** | **1.84** | < 0.001 |
| *Firmicutes* | *Ruminococcaceae* | *Intestinimonas* | 350256 | -0.58 | -0.23 | **1.65** | **2.00** | 0.001 |
| *Firmicutes* | *Ruminococcaceae* | *Anaerotruncus* | 315223 | 0.81 | 0.02 | **2.28** | **1.49** | < 0.001 |
| *Firmicutes* | *Ruminococcaceae* | *Butyricicoccus* | 308157 | 0.37 | 0.47 | **-1.55** | -1.45 | < 0.001 |
| *Firmicutes* | *Ruminococcaceae* | *Butyricicoccus* | 193240 | -1.08 | -1.34 | -1.42 | **-1.68** | < 0.001 |
| *Firmicutes* | *Ruminococcaceae* | *Butyricicoccus* | 40798 | 0.45 | 0.83 | **-4.40** | **-4.01** | < 0.001 |
| *Firmicutes* | *Ruminococcaceae* | *Butyricicoccus* | 1132942 | 0.64 | 0.37 | **-3.15** | **-3.43** | < 0.001 |
| *Firmicutes* | *Ruminococcaceae* | *Ruminococcus 2* | 847728 | -0.72 | -0.88 | **1.54** | 1.38 | 0.008 |
| *Firmicutes* | *Ruminococcaceae* | *Ruminococcaceae UCG-005* | 1094991 | -1.26 | **-1.67** | 1.01 | 0.59 | < 0.001 |
| *Firmicutes* | *Ruminococcaceae* | *Ruminiclostridium 9* | New.ReferenceOTU395 | -1.01 | -1.00 | **2.37** | **2.39** | < 0.001 |
| *Firmicutes* | *Ruminococcaceae* | *Ruminiclostridium 9* | 579244 | -1.11 | -1.32 | **2.49** | **2.28** | < 0.001 |
| *Firmicutes* | *Ruminococcaceae* | *Intestinimonas* | New.ReferenceOTU392 | -0.28 | -0.31 | **1.69** | **1.66** | 0.004 |
| *Firmicutes* | *Ruminococcaceae* | *uncultured* | 240271 | 0.20 | 0.33 | **1.89** | **2.02** | < 0.001 |
| *Firmicutes* | *Ruminococcaceae* | *uncultured* | 157772 | -0.27 | -0.36 | **1.68** | **1.59** | 0.001 |
| *Firmicutes* | *Ruminococcaceae* | *uncultured* | 443620 | -0.74 | 0.83 | 0.61 | **2.17** | 0.001 |
| *Firmicutes* | *Ruminococcaceae* | *Intestinimonas* | 519763 | 1.22 | 1.10 | **2.52** | **2.40** | < 0.001 |
| *Firmicutes* | *Ruminococcaceae* | *Family_Ruminococcaceae* | 364341 | 0.25 | 0.20 | **1.56** | **1.51** | < 0.001 |
| *Firmicutes* | *Ruminococcaceae* | *uncultured* | 647215 | 0.58 | 0.49 | **2.24** | **2.15** | < 0.001 |
| *Firmicutes* | *Ruminococcaceae* | *Oscillibacter* | 564334 | -0.38 | -0.21 | **2.35** | **2.52** | < 0.001 |
| *Firmicutes* | *Ruminococcaceae* | *Oscillibacter* | 362793 | 0.13 | 0.33 | **2.11** | **2.32** | < 0.001 |
| *Firmicutes* | *Ruminococcaceae* | *Oscillibacter* | 196831 | 0.14 | 0.24 | **2.09** | **2.19** | < 0.001 |
| *Firmicutes* | *Ruminococcaceae* | *Oscillibacter* | 585227 | 0.12 | 0.21 | **2.44** | **2.53** | < 0.001 |
| *Firmicutes* | *Ruminococcaceae* | *Ruminiclostridium* | 294545 | -0.23 | 0.21 | **1.73** | **2.16** | < 0.001 |
| *Firmicutes* | *Ruminococcaceae* | *Intestinimonas* | 842786 | -0.59 | -0.09 | **2.24** | **2.74** | < 0.001 |
| *Firmicutes* | *Ruminococcaceae* | *Ruminiclostridium 9* | 349246 | -1.09 | -0.39 | 1.04 | **1.74** | 0.001 |
| *Firmicutes* | *Ruminococcaceae* | *Ruminiclostridium 9* | 408513 | **-3.12** | **-2.29** | 1.43 | **2.26** | < 0.001 |
| *Firmicutes* | *Ruminococcaceae* | *uncultured* | New.ReferenceOTU377 | -0.22 | -0.81 | **1.91** | 1.32 | < 0.001 |
| *Firmicutes* | *Ruminococcaceae* | *uncultured* | New.ReferenceOTU551 | 0.00 | 0.58 | **2.70** | **3.27** | < 0.001 |
| *Firmicutes* | *Ruminococcaceae* | *Fournierella* | 510295 | 0.35 | 0.86 | **-2.43** | **-1.93** | < 0.001 |
| *Firmicutes* | *Streptococcaceae* | *Streptococcus* | New.ReferenceOTU486 | -0.10 | **-2.01** | -0.33 | **-2.24** | 0.029 |
| *Firmicutes* | *Streptococcaceae* | *Streptococcus* | New.ReferenceOTU364 | -0.92 | **-1.70** | -0.91 | **-1.68** | 0.017 |

The taxonomic composition and the log_2_ fold change (L2FC) of the DESEq2-normalized abundance of each OTU are shown

**Supplementary Table S8** – BLAST results and identity comparison of OTUs associated with microbiota-derived berberine metabolites *in vitro* and *in vivo*. OTUs significantly associated with berberrubine or thalifendine concentration either *in vitro* or *in vivo* revealed 3 shared genera. For each genus, OTU sequences derived from *in vitro* samples were aligned against OTUs sequences derived from *in vivo* samples of the same genus. Each OTU was blasted against 16S rRNA sequence NCBI database and results > 97% ID, or best hits (in grey), were reported.

|  | ***IN VITRO*** | | |  | ***IN VIVO*** | | |
| --- | --- | --- | --- | --- | --- | --- | --- |
| **Genus to which the OTU belongs** | **OTU number** | **Associated metabolite** | **BLAST result (% ID)** | **Percent identity between sequences (%)** | **OTU number** | **Associated metabolite** | **BLAST result (% ID)** |
| ***Blautia*** | 1. 549635 | (+) Berb, Thal | *Blautia glucerasea* (99.75) | 1 vs 1 96.52  1 vs 2 96.52  2 vs 1 96.52  2 vs 2 96.52 | 1. 180610 | (+) Thal | *Blautia stercoris* (97.01) |
|  | 2. New.ReferenceOTU313 | (-) Berb | *Blautia luti* (97.77) / *argi* (97.76), *Gluceribacter canis* (97.01) |  | 2. New.ReferenceOTU405 | (+) Berb | *Blautia faecicola* (97.01), *Murimonas intestini* (97.03) |
| ***Clostridiales vadin***  ***BB60 group*** | 1. 592913 | (+) Berb, Thal | *Christensenella timonensis* (85.75) | 1 vs 1 91.58  1 vs 2 91.34  2 vs 1 90.12  2 vs 2 89.88  3 vs 1 87.16  3 vs 2 86.81 | 1. 158302 | (+) Berb | *Christensenella massiliensis* (86.24) |
|  | 2. New.ReferenceOTU182 | (+) Thal, Berb | *Gracilibacter thermotolerans* (86.95) |  | 2. 228798 | (+) Berb | *Christensenella massiliensis* (86.00) |
|  | 3. New.ReferenceOTU474 | (+) Berb, Thal | *Alkalibaculum sporogenes* (84.03) |  |  |  |  |
| ***Undefined***  ***Lachnospiraceae*** | 1. 761968 | (+) Thal, Berb | *Ruminococcus faecis* (96.25) | 1 vs 1 95.00 | 1. 548503 | (+) Thal | *Ruminococcus lactaris* (97.26), *Fusicatenibacter* *saccharivorans* (97.01) |

Berb: Berberrubine; Thal: Thalifendine.
